# TXNIP mediates the differential responses of A549 cells to sodium butyrate and sodium 4‐phenylbutyrate treatment

**DOI:** 10.1002/cam4.977

**Published:** 2016-12-29

**Authors:** Xuefang Jin, Nana Wu, Juji Dai, Qiuxia Li, XiaoQiang Xiao

**Affiliations:** ^1^ School of Basic Medical SciencesWenzhou Medical UniversityWenzhouChina; ^2^ The Institute of Genomic MedicineWenzhou Medical UniversityWenzhouChina; ^3^ Department of General Surgerythe First Affiliated Hospital of Wenzhou Medical UniversityWenzhou Medical UniversityWenzhouChina; ^4^ Joint Shantou International Eye CenterShantou University & the Chinese University of Hong KongShantouChina

**Keywords:** A549 cells, butyrate, histone modification, mitochondrial superoxide, TXNIP

## Abstract

Sodium butyrate (NaBu) and sodium 4‐phenylbutyrate (4PBA) have promising futures in cancer treatment; however, their underlying molecular mechanisms are not clearly understood. Here, we show A549 cell death induced by NaBu and 4PBA are not the same. NaBu treatment induces a significantly higher level of A549 cell death than 4PBA. A gene expression microarray identified more than 5000 transcripts that were altered (>1.5‐fold) in NaBu‐treated A549 cells, but fewer than 2000 transcripts that were altered in 4PBA. Moreover, more than 100 cell cycle‐associated genes were greatly repressed by NaBu, but slightly repressed by 4PBA; few genes were significantly upregulated only in 4PBA‐treated cells. Gene expression was further validated by other experiments. Additionally, A549 cells that were treated with these showed changes in glucose consumption, caspase 3/7 activation and histone modifications, as well as enhanced mitochondrial superoxide production. TXNIP was strongly induced by NaBu (30‐ to 40‐fold mRNA) but was only slightly induced by 4PBA (two to fivefold) in A549 cells. TXNIP knockdown by shRNA in A549 cells significantly attenuated caspase 3/7 activation and restored cell viability, while TXNIP overexpression significantly increased caspase 3/7 activation and cell death only in NaBu‐treated cells. Moreover, TXNIP also regulated NaBu‐ but not 4PBA‐induced H4K5 acetylation and H3K4 trimethylation, possibly by increasing WDR5 expression. Finally, we demonstrated that 4PBA induced a mitochondrial superoxide‐associated cell death, while NaBu did so mainly through a TXNIP‐mediated pathway. The above data might benefit the future clinic application.

## Introduction

Histone post‐translational modification is one of the epigenetic mechanisms controlling longevity and the development of various diseases, including tumors, in diverse organisms 1, 2. Histone protein acetylation and methylation are dynamic processes that change DNA accessibility and thus regulate gene expression by influencing chromatin structure 1, 2. Several types of acetylated histones, such as histone H3 lysine 9 (acH3K9), histone H4 lysine 5 (acH4K5) and histone H3 lysine 18 (acH3K18), have been identified, and their roles in different cellular processes have been gradually determined 3. Histone acetyltransferases (HATs) and histone deacetylases (HDACs) are responsible for the balance of histone acetylation modification with physiological status. Three lysine methylation statuses have been identified, namely, mono, di, and trimethyl groups (me1/2/3) 4. Different lysine sites undergoing methylation correlate with either transcriptional activation, such as H3K4 4, or transcriptional repression, such as H3K9 and H3K27 5. Histone methylation might also promote DNA methylation, causing the further repression of the affected genes 1, 2. Lysine 4 of histone H3 is primarily methylated by the lysine (K)‐specific methyltransferase 2 (KMT2) family of enzymes 1, 2. H3K4 trimethylation (H3K4me3) is catalyzed by KMT2A complexes, also called COMPASS (complex of proteins associated with Set1)‐like complexes, which include catalytic subunits, such as MLL1 (mixed‐lineage leukemia 1), and multiple regulatory subunits for WDR5 (WD40 repeat‐containing protein 5), RBBP5 (retinoblastoma‐binding protein 5), ASH2 (absent, small or homoeotic discs 2) and DPY30 [containing the WRAD subcomplex (WDR5‐ASH2‐RBBP5‐DPY30 complex)] 6.

Butyrate and phenylbutyrate, inhibitors of the enzyme activities of HDACs, prevent the deacetylation of histone and therefore control gene expression. Being a short‐chain fatty acid, butyrate is metabolized by intestinal bacterial from soluble fibers that are produced by butyrate‐produced bacteria, by mammal cells through fatty acid oxidation and glucose metabolism although in much lower concentration and is also abundant in milk fat, plant oil, and butter 7. Accumulating evidence indicates that NaBu inhibits cell proliferation and induces the apoptosis of a variety of tumor cells 8, 9. Given the growth‐inhibiting and apoptosis‐inducing activities of NaBu in many tumor cells in vitro and in vivo, the clinical application of NaBu, alone or in combination with other anti‐cancer reagents, is restricted because of the absence of a detailed molecular mechanism 10. Therefore, a detailed examination of its molecular characterization and network of targets will benefit its clinical application. Here, 4PBA has potentially favorable effects on many diseases, including cancers, genetic metabolic syndromes, neuropathies, diabetes, and hemoglobinopathies 11, 12. Currently, 4PBA has been approved for the treatment of urea cycle disorders and is under investigation in cancers, hemoglobinopathies, motor neuron diseases, and cystic fibrosis clinical trials 13. Also, 4PBA is also used to treat ornithine transcarbamylase deficiency (OTCD) and a potential reagent with therapeutic effect in hyperbilirubinemia 13. These diverse functions that 4PBA exerts are due to its three important characteristics: ammonia scavenging, enabling the excretion of ammonia; chaperoning, rescuing conformational abnormalities of proteins; and histone deacetylase, inhibiting the regulation of gene expression. Interest in 4PBA is growing worldwide 1, 2, 13.

Thioredoxin‐interacting protein (TXNIP), which belongs to the arrestin family and is induced by various stimuli, interacts with and inhibits thioredoxin activity 14. By negatively regulating thioredoxin activity, TXNIP prevents the scavenging of the reactive oxygen species in the cell and provokes cellular oxidative stress, causing a variety of physiological responses, including cell proliferation and death 15. TXNIP suppresses tumor development, regulates glucose metabolism and the inflammatory signaling pathway, and controls gene expression as a transcriptional negative regulator by binding to the HDAC1 and HDAC3 proteins 16, 17, 18. However, the detailed molecular mechanism by which TXNIP mediates a variety of biological processes induced by HDAC inhibitors is not clearly understood.

Lung cancer is the leading cause of cancer death worldwide; in particular, non‐small‐cell lung cancer (NSCLC) accounts for approximately 85% of all lung cancer cases. Histone deacetylase inhibitors constitute a new and promising drug family with potential anticancer activity. To better understand their molecular mechanisms and to efficaciously prevent their side effects, we compared the response of A549 cells to NaBu and 4PBA at the cellular and molecular levels and found that TXNIP partially mediates the differential response of A549 cells to NaBu and 4PBA.

## Materials and Methods

### Cell line, culture conditions and total RNA preparation

A549, a human non‐small‐cell lung carcinoma cell line, was grown in RPMI 1640 medium (GIBCO) with 10% fetal bovine serum (FBS) (GIBCO). HEK293T cells were grown in DMEM with 10% FBS. A549 and HEK293T cells were incubated with various concentrations of NaBu (Sigma, USA) or 4PBA (Sigma, USA) for the indicated times and concentrations. Total RNA was extracted from cultured cells, using the TRIzol method (Life‐Technologies, USA) according to the manufacturer's instructions. The RNA samples were quantified, using a spectrophotometer and visualized on an agarose gel for quality assurance.

### Antibodies and reagents

The primary antibodies that were used were rabbit anti‐histone H4 lysine 5 acetylation (#9627, CST), rabbit anti‐lysine acetylation (ab190479; Abcam), rabbit anti‐histone H3 lysine 18 acetylation (AJ1357a; Abgent, USA), anti‐CyclinB1 (ab151269; Abcam), rabbit anti‐histone H4 lysine 9 acetylation (07‐352; Millipore), anti‐GAPDH (92590; Millipore, USA), anti‐CTGF (ab6992; Abcam, USA), mouse anti‐p21 (SC‐817; Santa Cruz), rabbit anti‐TXNIP (ab188865, Abcam,USA), goat anti‐gamma H2AX (Santa Cruz), mouse anti‐BST2 (ab88523; Abcam), rabbit anti‐Histone H3 lysine 4 trimethylation (07‐437; Millipore), rabbit anti‐Histone H3 trimethylation lysine 27 (07‐449; Millipore), rabbit anti‐Histone H3 trimethylation lysine 9 (07‐442; Millipore), and goat anti‐GFP (ab6673; Abcam). The secondary antibodies that were used were anti‐rabbit IgG‐HRP (ab191866 Abcam), anti‐mouse IgG‐HRP (ab193651 and ab193652; Abcam), donkey anti‐goat‐HRP (31400, Pierce), and DyLight 488 Goat Anti‐Rabbit (111‐055‐003, Jackson).

### Establishment of stable TXNIP‐knockdown A549 cell line

HEK293T cells (1 × 10^5 ^cells/mL) were seeded on 60 mm cell culture dishes 1 day before transfection. Transfection was performed using psi‐LVRH1GP‐shTXNIP (5 *μ*g) and an appropriate amount of the lentiviral package plasmids system with lipofectamine 3000 (Invitrogen, USA). After 72 h of transfection, the lentiviral particles were harvested by collecting and centrifuging the supernatant. A549 cells were infected with the above supernatant and screened for stable TXNIP‐knockdown cell clones by puromycin.

### Microarray assays

A549 cells that had been treated with 5 mmol/L NaBu or 5 mmol/L 4PBA or vehicle for 24 h were immediately lysed with Trizol reagent (Invitrogen). The total RNA was extracted according to the manufacturer's instructions and digested with RNAse‐Free DNAse. The quality of the total RNA was tested and confirmed using an RD2000 (Genetech, Inc., Shanghai, China), and cDNA was synthesized using the GeneChip^®^ 3′ IVT Express Kit (Affymetrix, Inc., CA, USA). The microarray assay was performed by Genetech, Inc., using human Affymetrix GeneChip arrays (Affymetrix, Inc., USA). This chip covers the whole human genome with >19,100 unique genes targeting a total of 45,281 transcripts. The results were analyzed using the cubic spline normalization method without background subtraction. Briefly, the biotinylated RNA was purified, fragmented, and used for hybridization. The microarray was prehybridized and hybridized, using the GeneChip hybridization, wash and staining kit (Affymetrix, Inc.) in a GeneChip hybridization oven. The hybridized microarray was washed in a fluidics station 450 system, and the step‐by‐step operations were controlled, using inbuilt AGCC control software (Affymetrix, Inc). The GeneChip Scanner 3000 (Affymetrix, Inc) was used for microarray scanning, and the resulting primary data in DAT format were transformed into CEL format, using the AGCC software. The Expression Console software was used for the Quality control of the primary data from the scanning the microarray, and Partek software was used for RMA processing and data analysis. Statistical significance cutoff levels were set for individual transcripts at *P *≤* *0.05 based on an ANOVA assay and fold change (upregulated or downregulated) ≥2 in the mRNA level; any transcript that met these thresholds was deemed differentially expressed. The results were submitted to the National Center for Biotechnology Information (NCBI) Gene Expression Omnibus (GEO) database (GSE58571).

### Vector construction

The promoter sequences of DTL, TXNIP, and CTGF were cloned using the genomic DNA extracted from A549 cells as a template with the primers pairs in Table 1 and then inserted into pGL‐3Basic. The resulting plasmids were designated pDTL, pTXNIP, and pCTGF. The cloned sequences were confirmed by sequence. Regarding the GFP‐TXNIP protein expression vector construction, the sequence was cloned from cDNA that had been reverse‐transcribed from NaBu‐treated A549 cells using the primer pairs in Table 1 and inserted into the pEGFP‐C3 vector that had been digested with the EcoRI and Xhol endonucleases. The resulting plasmid was designated GFP‐TXNIP. The TXNIP small interfering RNA expression vector was constructed by inserting the synthesized DNA sequence (Gaggtgtgtgaagttactc‐targeted ORF) into psi‐LVRH1GP.

**Table 1:** Primer sequences and usage

Gene and primer name	Usages and sequences
Thymidylate synthetase (TS)	RT‐PCR Primers
Forward primer	5′‐ACCAACCCTGACGACAGAAG‐3′
Reverse primer	5′‐ATGCGGATTGTACCCTTCAA‐3′
Histone cluster 1, H1c (HC1)	RT‐PCR Primers
Forward primer	5′‐TCCAGTTCCCAGTGGGCCGTG‐3′
Reverse primer	5′‐CGTTGCGGATGGCCAGCTGCA‐3′
ISG15	RT‐PCR Primers
Forward	5′‐TGTCGGTGTCAGAGCTGAAG‐3′
Reverse	5′‐GCCCTTGTTATTCCTCACCA‐3′
Cbp/p300‐interacting transactivator, with Glu/Asp‐rich carboxy‐terminal domain, 2 (CITED2)	RT‐PCR/qPCR
Forward	5′‐CAGGAAGGTCCCCTCTATGTG‐3′
Reverse	5′‐GCGCCGTAGTGTATGTGCTC‐3′
Connective tissue growth factor (CTGF)	RT‐PCR/qPCR
Forward	5′‐TGACGAGCCCAAGGACCAAAC‐3′
Reverse	5′‐GGCTTGGAGATTTTGGGAGTAC‐3′
Bone marrow stromal cell antigen 2 (BST2)	RT‐PCR/qPCR
Forward	5′‐AAGGGCTTTCAGGATGTGGAG‐3′
Reverse	5′‐AGACGCGTCCTGAAGCTTATG‐3′
Thioredoxin‐interacting protein (TXNIP)	RT‐PCR/qPCR
Forward	5′‐TGGATCTGGTGGATGTCAATAC‐3′
Reverse	5′‐TCTGAGTCAGCACCTTGGTCTG‐3′
Insulin‐Like Growth Factor Binding Protein 1 (ILGFBP‐1)	RT‐PCR
Forward	5′‐CGTGCAGGAGTCTGACGCCTC‐3′
Reverse	5′‐ACTCTCTACGACTCTGTAGAG‐3′
Cell division cycle associated 5 (CDCA5)	RT‐PCR
Forward	5′‐GCAGTCAGAAAGCCCATCGTC‐3′
Reverse	5′‐TCCAGCTCTCCTTCCTTGGAG‐3′
Aurora kinase A (AKA)	RT‐PCR
Forward	5′‐AATATGCACCACTTGGAACAG‐3′
Reverse	5′‐TCCACCTTCTCATCATGCATC‐3′
Cell division cycle 45 homolog (*S. cerevisiae*) (CDC45H)	RT‐PCR/qPCR
Forward	5′‐AGTTCCCGCCTATGAAGACATC‐3′
Reverse	5′‐GCCAGCTCAAACATCACCATG‐3′
Cell division cycle 25 homolog C (*S. pombe*) (CDC25H)	RT‐PCR/qPCR
Forward	5′‐TGTAGCACTCCGAATGGTTTG‐3′
Reverse	5′‐CGATATAGGCCACTTCTGCTC‐3′
Aurora kinase B (AKB)	RT‐PCR
Forward	5′‐ATCAGCTGCGCAGAGAGATCGAAA‐3′
Reverse	5′‐CTGCTCGTCAAATGTGCAGCTCTT‐3′
Chemokine (C‐X‐C Motif) Ligand 5(CL5)	RT‐PCR
Forward	5′‐TGCTGCTGCTGACGCAGCCAG‐3′
Reverse	5′‐TTCCTTCCCGTTCTTCAGGGA‐3′
Denticleless homolog (Drosophila) (DTL)	RT‐PCR
Forward	5′‐AGACGAGAATACCTTAGTCTC‐3′
Reverse	5′‐ TAGAGTTCTGGTGTCCATTG‐3′
Ubiquitin‐conjugating enzyme E2C (UCEE2C)	RT‐PCR
Forward	5′‐GGACCATCCATGGAGCAGCTG‐3′
Reverse	5′‐TGGAGAGCAGAATGGTCCTGA‐3′
Mitogen‐activated protein kinase 4 (MAPK4)	RT‐PCR
Forward	5′‐GATCGTTGATCAGCATTACTC‐3′
Reverse	5′‐CAGCTCGTCCTTGTCTTCCTC‐3′
Cyclin‐dependent kinase 1 (CDK1)	RT‐PCR
Forward	5′‐TGGTCAGTACATGGATTCTTC‐3′
Reverse	5′‐TGTCAACTGGAGTTGAGTAAC‐3′
Annexin A13 (AXA13)	RT‐PCR
Forward	5′‐AGGTCCTGTGCACGAGGACCA‐3′
Reverse	5′‐CCAACGCAAGCTCATCAGTGC‐3′
Cell Division Cycle Associated 8 (CDCA8)	RT‐PCR
Forward	5′‐TCTGAAAGACTTCGACCGTGA‐3′
Reverse	5′‐ATTTCATCTACCTGTATTACC‐3′
SPC25, NDC80 kinetochore complex component, homolog (*S. cerevisiae*) (SPC25)	RT‐PCR
Forward	5′‐TGACTGCAAATATCCAGGATC‐3′
Reverse	5′‐TTCTTAGGGTCAATATTAGTG‐3′
E2F transcription factor 7 (E2FIT7)	RT‐PCR
Forward	5′‐TGTCACTCTGGATGTGGCTGC‐3′
Reverse	5′‐GCACAGACTTGAATCTGGCCA‐3′
RAS p21 protein activator 4 (RASp21PA4)	RT‐PCR
Forward	5′‐GGAATGAGACGTTTGAATTTG‐3′
Reverse	5′‐AGCTGCAAGGAGCCCAGGTTG‐3′
Cyclin B1 (CCNB1)	RT‐PCR/qPCR
Forward	AACCTCCAAGCCCGGACTGAGG
Reverse	GCTCAGGTTCTGGCTCAGGTTC
TXNIP promoter assay	Luciferase reporter gene vector construction
pTXNIP‐FP1	5′‐CGGCCGCTCGAGATGGCCCGGGCTGGTATTGGGGT‐3′
pTXNIP‐RP1	5′‐CCCAAGCTTTAAGGTATTCTTAAGCAGTTTGAGC‐3′
DTL promoter assay	Luciferase reporter gene vector construction
pDTL‐FP1	5′‐CGGGGTACCGCTATGGAAATCAAATGAATCGC‐3′
pDTL‐RP1	5′‐CGGCCGCTCGAGGAAAAGTCGTAGAAATGCCTCC‐3
CTGF promoter assay	Luciferase reporter gene vector construction
CTGF‐p‐FP‐Xhol1	5′‐CGGCCGCTCGAGACGCGTCTTTGTTCTCTTTCTTGTCCC‐3′
CTGF‐p‐RP‐HindIII	5′‐GCAAGCTTCTGTCGTCTCGGGGCTGTCGGCCG‐3′
GFP‐TXNIP expression vector	GFP‐TXNIP overexpression vector construction
GFP‐TXNIP‐Xhol‐FP	5′‐GCCTCGAGATGGTGATGTTCAAGAAGATCAAGT‐3′
GFP‐TXNIP‐EcoRI‐RP	5′‐GCGAATTCGCTCACTGCACATTGTTGTTGAGGAT‐3′

### RT‐PCR and qRT‐PCR

RT‐PCR was carried out using a two‐step strategy: cDNA was generated using a Reverse Transcription kit (Thermo Scientific, USA) in the first step. Then, using gene‐specific primer sets, RT‐PCRs were carried out using the designated cDNA as a template. Glyceraldehyde‐3‐phosphate dehydrogenase (GAPDH) was used for internal normalization. The sequences of the forward and reverse primers used for RT‐PCR are given in Table 1. Real‐time qPCR was performed in an ABI single‐color real‐time PCR detection system, using SYBR Green supermix (Takara, Daliang China). The primer sequences that were used for qPCR are listed in Table 1. These primers were synthesized by Sangong Biotech Co., Ltd. (Shanghai, China). The cycling conditions were as follows: step 1: 95°C for 10 min; step 2: 40 cycles of 94°C for 10 sec and 60°C for 30 sec; and step 3: 72°C for 10 min. The annealing temperature was modified based on the characterization of the specific gene primers. The relative expression levels of the genes were calculated, using the 2^−ΔΔC^T method using *β*‐actin mRNA as an internal control.

### Glucose concentration measurement

To investigate the glucose consumption of cells undergoing NaBu or 4PBA treatment, we measured the glucose concentration in the medium, using a blood glucose monitoring system containing a blood glucose meter and glucose test strips (Wetrust, China). The protocol was strictly based on the manufacturer's instructions. Briefly, the blood glucose test strips were inserted into the meter before measurement. Then, 5 *μ*L of medium was placed onto the strips for 6 sec, and the result was then obtained from the meter.

### Western blotting

For the protein expression assay, the target cells were harvested and washed twice with cold PBS. The cell pellets were then resuspended in ice cold 1× NP‐40 lysis buffer containing complete protease inhibitors (Roche, USA) for three liquid nitrogen/37°C cycles to lyse the cells. The supernatants were obtained by centrifuging the above lysates at 13,000*g* for 15 min at 4°C, and their total protein concentrations were determined by a Bio‐Rad protein assay, using Dye Reagent (BioRad, USA). Then, the samples were subjected to SDS‐PAGE under reducing conditions and then transferred onto PVDF membranes (BioRad, USA). The blotted membranes were then blocked with specific buffers or 5% nonfatty milk and probed with the designated primary antibodies (4°C, Overnight) depending on the experiment. The secondary HRP‐conjugated antibodies were incubated at room temperature (RT) for 1–2 h, and the membranes were washed at least 4 times with TBST buffer. Finally, the immunoreactive proteins were visualized using enhanced chemiluminescence (ECL, BioRad).

### Flow cytometric apoptosis assay

To measure the annexin V binding and propidium iodide (PI) staining of A549 cells, cells (10^6^ cells) that had been treated with NaBu or 4PBA, the cells were harvested and stained with FITC‐labeled annexin V and PI (Molecular Probes, Eugene, OR) as specified by the supplier. Briefly, A549 cells (1 × 10^6^) in 6‐well cell culture plates were cultured overnight as indicated and then treated with 5 mmol/L NaBu or 4PBA or a negative control, washed, and stained with PI and annexin V‐FITC in the annexin‐binding buffer. Thereafter, the cells were analyzed within 1 h using CellQuest software (BD Biosciences, San Jose, CA) by FACSCalibur. Data from 10^6^ cells were analyzed for each sample.

### Detection of caspase‐3/7 activity

The enzymatic activity of caspase‐3/7 was measured, using the Caspase‐Glo 3/7 Assay kit (Promega, Shanghai, China) according to the manufacturer's instruction. Briefly, cells were seeded on 96‐well plates and treated with or without 5 mmol/L 4PBA or NaBu for 48 h. Then, the cells were lysed and incubated with 100 *μ*L of Apo‐ONE Caspase‐3/7 reagent. After 1 h of incubation in the dark at RT, the fluorescence of each well was measured at 485–520 nm by reading in an Epoch microplate reader (BioTek Instruments; Winooski, VT).

### Detection of the mitochondrial superoxide levels

To measure the mitochondrial superoxide generation in living cells, we used MitoSOX^™^ Red mitochondrial superoxide indicator (M36008; Life Technology), a fluorogenic dye that is live‐cell permeant and is rapidly and selectively targeted to the mitochondria. Once in the mitochondria, MitoSOX Red reagent is oxidized by superoxide and exhibits bright red fluorescence upon binding to nucleic acids. Briefly, the cells were pretreated with 4PBA or NaBu at the designated concentration and duration. The cells were then washed with fresh medium and then incubated in medium‐containing MitoSOX Red (5 *μ*mol/L) and 4PBA or NaBu for a further 30 min at 37°C in the dark. The cells were washed with fresh serum‐free medium, and following washing, the fluorescence intensity of the cells was measured by a Flow cytometric assay.

### Statistical analysis

All of the data were analyzed, using GraphPad prism 5 software and are represented as the mean ± SD. Data from multiple groups were quantified, using a one‐way ANOVA or Student's *t*‐test, and comparisons of the two groups were quantified by Student's *t*‐test.

## Results

### Comparative analysis of A549 cells in response to NaBu or 4PBA

**Figure 1:**
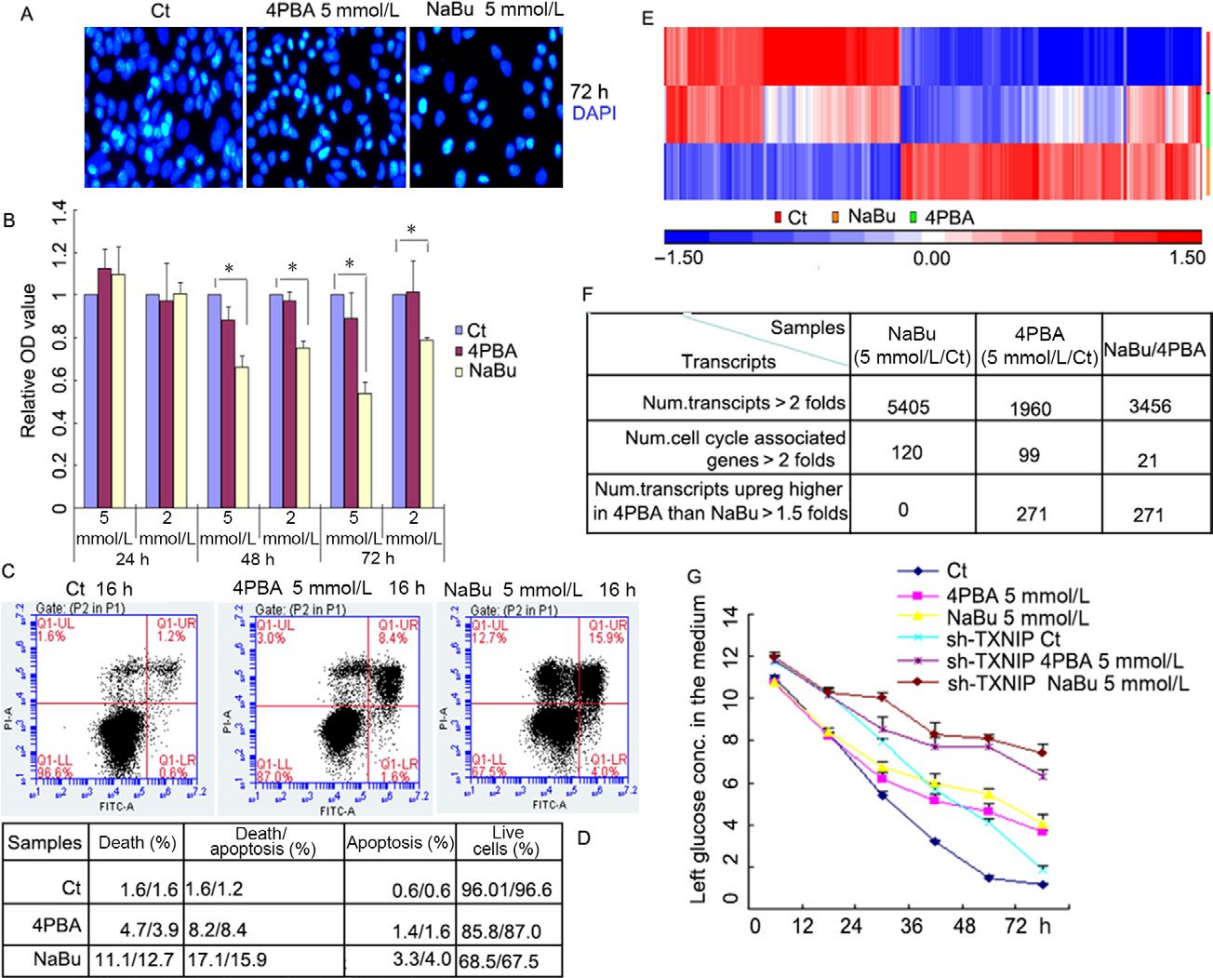
Comparative analysis of the response of A549 cells to NaBu or 4PBA treatment. (A) A549 cells were seeded on 6‐well cell culture plates and exposed to 5 mmol/L NaBu or 4PBA or vehicle (C_*t*_) for 72 h; the cell nucleus was stained with DAPI (blue). (B) A549 cells were seeded on 96‐well cell culture plates and incubated with NaBu (5 mmol/L or 2 mmol/L) or 4PBA (5 mmol/L or 2 mmol/L) or vehicle (C_*t*_) for the designated durations; then, the cell viability was analyzed using an MTT assay. (C) A549 cells were seeded on 6‐well cell culture plates, treated with 5 mmol/L NaBu or 5 mmol/L 4PBA for 16 h and harvested for Annexin V‐FITC and propidium iodide analysis via Flow cytometry. The results show the annexin V (*x*‐axis) and propidium iodide (*y*‐axis) levels. (D) The results of three replications of Flow cytometry are shown in the table assayed from (C, E), A549 cells were treated with 5 mol/L NaBu, 5 mol/L 4PBA or negative control for 24 h. Then, the cells were harvested for total RNA extraction with TRIzol reagent and used for a gene microarray assay, using the Affymetrix GeneChip 3′ IVT Express Kit. The heatmap shows all of the differentially expressed transcripts with a 1.5‐fold change (*P* < 0.005). The microarray Gene expression data were log2 transformed and then quantile normalized prior to generating the Heatmap for a direct comparison of the data. The differentially expressed transcripts (red or green indicate upregulated or downregulated, respectively) in each sample were mapped by lane for A549 cells under NaBu, 4PBA or negative control treatments. (F) Table shows the number of transcripts that changed by more than twofold under the NaBu/Ct, 4PBA/Ct or NaBu/4PBA treatment; the number of transcripts associated with the cell cycle that changed by twofold; and the number of transcripts that changed fold only in 4PBA. (G) Glucose consumption was measured using a blood detection system by placing the medium onto the strips for 6 sec after the designated treatment. The results in all of the histograms are the means ± S.D. for three independent experiments. C_*t*_, control; 4PBA, sodium phenylbutyrate; NaBu, sodium butyrate.

The inhibitory effect of butyrate on cancer cell growth is mainly attributed to its function as a histone deacetylase inhibitor that alters the expression of many genes with diverse functions, including cell proliferation, apoptosis, and differentiation 19, 20. As a derivative of butyrate, 4‐phenylbutyrate also plays multifaceted roles in many cellular processes. Therefore, we designed experiments to examine the ability of these two inhibitors to induce the death A549 cells, a non‐small‐cell lung cancer cell line. We treated A549 cells with 5 mmol/L NaBu, or 5 mmol/L 4PBA for 72 h and then stained them with DAPI, a nuclear, specific fluorescence dye. We found that both inhibitors can significantly prevent A549 cell proliferation and promote cell death; moreover, NaBu‐treated A549 cells showed a lower proliferation and cell viability compared to those of 4PBA‐treated cells (Fig. 1A). Furthermore, we detected cell viability after treatment with 2 mmol/L or 5 mmol/L NaBu or 4PBA for 24, 48 or 72 h via MTT and found that 2 mmol/L 4PBA caused no significant change in cell viability at the designated time points, while 5 mmol/L decreased cell viability at 72 h. There was a significant decrease in cell viability after treatment with 2 mmol/L or 5 mmol/L NaBu for 48 or 72 h (Fig. 1B). An apoptosis assay on 4PBA‐ and NaBu‐treated A549 cells was performed, using an Annexin V‐FITC and PI‐double‐staining kit and FACS. The results showed that both inhibitors can prevent A549 cell proliferation and induce cell death; the average values were approximately 4% for necrosis, 8.2% for both necrosis and apoptosis, and 1.5% for apoptosis in 4PBA‐treated cells and approximately 12% for necrosis, 16% for both necrosis and apoptosis, and 4% for apoptosis in NaBu‐treated cells (Fig. 1C and D). To further explore the underlying molecular mechanisms, we characterized the gene expression patterns after NaBu or 4PBA treatment, using a gene expression microarray. We used approximately 45,281 transcript sequences as probes, and of these, 5406 were identified as being significantly differentially expressed (*P* < 0.05), with a greater than 1.5‐fold change in expression between the NaBu‐treated group and the control group, while only 1961 such transcripts were identified for the 4PBA‐treated group (Fig. 1E and F). The detailed results were submitted to the National Center for Biotechnology Information (NCBI) Gene Expression Omnibus (GEO) database (GSE86928). Interestingly, NaBu greatly repressed the expression of genes involved in the cell cycle, but 4PBA had no or a slightly repressed effect (Fig. S1 and S2). We found that the transcriptional levels of transcripts of the *arrestin domain containing* family were upregulated, particularly those of *arrestin domain containing 4* and *TXNIP*, which were highly induced by NaBu (20‐ to 50‐fold), but slightly induced by 4PBA (approximately twofold). Of the 1961 transcripts, more than 120 transcripts were significantly changed (approximately fourfold) in the 4PBA‐treated group than in the control group, and more than 400 transcripts were significantly changed in the NaBu‐treated group than in the control group (Fig. S1 and S2). The *ankyrin repeat domain 22*,* four and a half LIM domains 1*,* perilipin 2*,* interleukin 8*,* peroxidasin homolog (Drosophila)*,* protein phosphatase 1*,* regulatory (inhibitor) subunit 1C*,* doublecortin‐like kinase 1*,* brain expressed, associated with NEDD4 and 1*,* stanniocalcin 1*,* S100 calcium‐binding protein A9*,* cellular retinoic acid‐binding protein 1, nephroblastoma overexpressed gene,* and *thymosin beta 15A* transcripts were all upregulated in 4PBA‐treated A549 cells. Because TXNIP is a negative regulator of glucose uptake 17, we compared the glucose consumption in A549 cells stably expressing shTXNIP and shScramble undergoing NaBu, 4PBA or vehicle treatment. The results showed that in wild type, both NaBu and 4PBA can decrease the glucose consumption compared to the vehicle control. In TXNIP‐knocked down A549 cells, glucose consumption under both NaBu and 4PBA stimulation also decreased compared to that under vehicle control. Interestingly, at 72 h, the glucose consumption in both NaBu‐ and 4PBA‐treated cells was the same as that in the wild type, but in TXNIP‐knockdown cells, the glucose consumption was significantly different (Fig. 1G). These results suggest that in A549 cells, NaBu and 4PBA cause different cellular and molecular responses.

### RT‐PCR, qPCR, western blot and reporter gene assays were consistent with the microarray data

**Figure 2:**
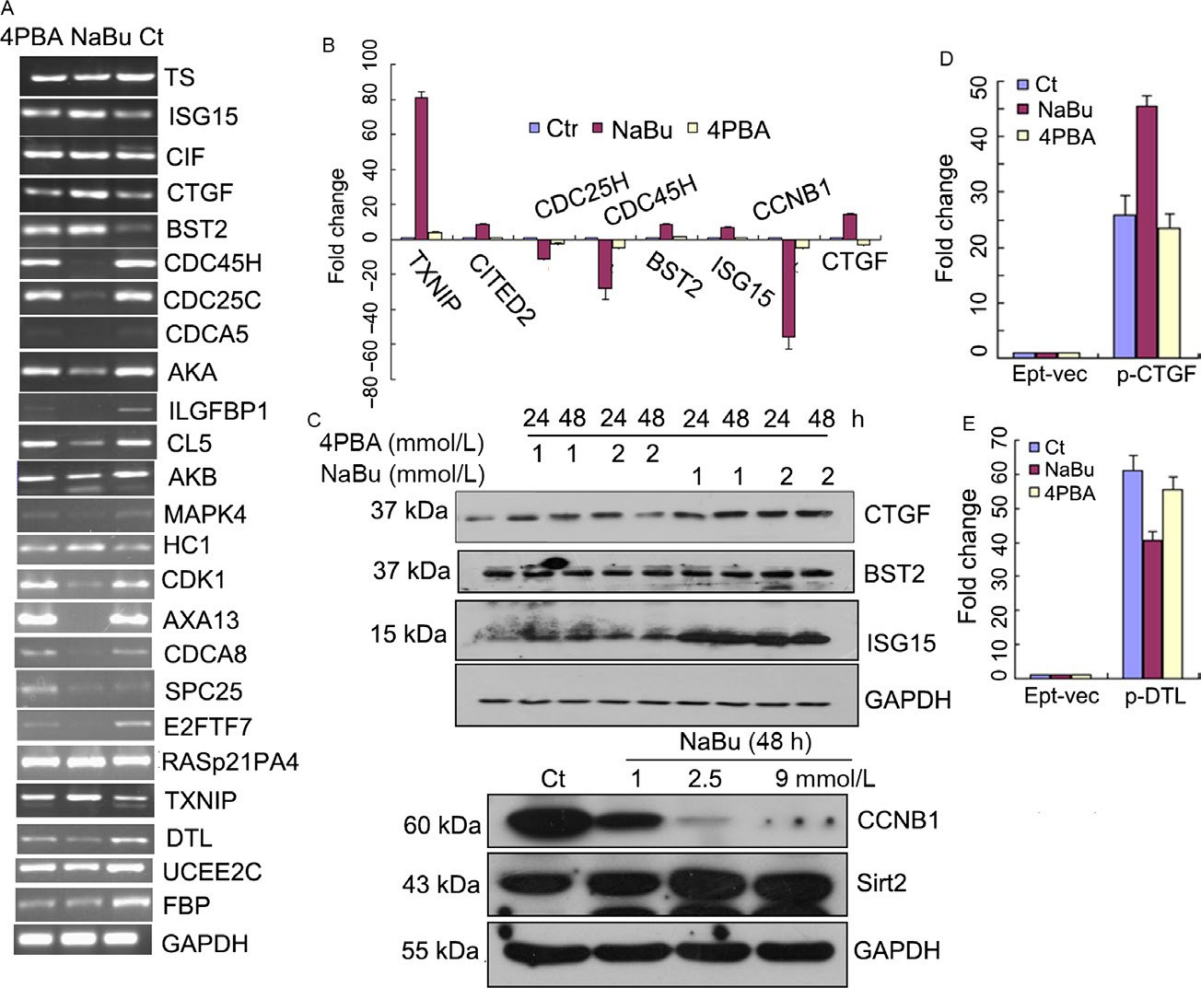
Results of RT‐PCR, qPCR, western blot and reporter gene assays were consistent results with the microarray data. (A) A549 cells were treated with NaBu and 4PBA via the same process as for the gene expression microarray, and the total RNAs were extracted with TRIzol for cDNA synthesis. RT‐PCR was performed with the designated primers listed in Table 1 and with an equal amount of cDNA as a template. (B) qRT‐PCR was performed with the same cDNA as in B and with the primers provided in the SYBR green kit. (C) Western blotting was performed after the same treatment as in A with the designated antibodies for CTGF, BST2, ISG15, CCNB1, Sirt1, and GAPDH. (D) Promoter activity assays were performed to analyze the transcriptional activities after treatment with NaBu or 4PBA. The promoters of DTL and CTGF were amplified from the A549 cell genomic DNA and cloned into pGL‐3Basic. The resulting vectors were cotransfected into 293T along with the reference vector Renilla luciferase for 12 h and then treated with NaBu, 4PBA or vehicle for another 24 h. The cells were incubated for 24 h and analyzed for luciferase activity with the Dual‐Luciferase Assay System (Promega). Firefly luminescence was normalized to Renilla luminescence and reported as relative luciferase activity. All of the experiments were performed in triplicate and repeated at least three times. The results in all of the histograms are the means ± S.D. for three independent experiments. C_*t*_, control; 4PBA, sodium phenylbutyrate; NaBu, sodium butyrate; Ept‐vec, p‐GL‐3Basic; pCTGF, p‐GL‐3Basic‐CTGF promoter; pDTL, p‐GL‐3Basic‐DTL promoter.

Because we did not have 2–3 biological replications for the microarray assay, we performed many other validation experiments to confirm the quality of the data that were obtained from the microarray assay. We randomly selected 33 genes for further validation by RT‐PCR. The RT‐PCR data were almost identical to the microarray data (Fig. 2A). Several genes were selected for a qRT‐PCR assay to further verify the quality of the microarray data. As expected, the results from the qRT‐PCR were also consistent with those from the microarray analysis (Fig. 2B). A western blot was used to validate the expression changes that were detected in the microarray, and consistent results were also obtained (Fig. 2C). We further selected two genes for a promoter activities assay by inserting the promoter sequences into the luciferase reporter system. The promoter activities of CTGF increased twofold under NaBu treatment and showed no obvious change under 4PBA treatment. The promoter activities of DTL decreased by 30% under NaBu treatment and showed no obvious change under 4PBA treatment compared to those under the vehicle control treatment (C_*t*_) (Fig. 2D and E). The luciferase assay results were consistent with the transcriptional data for both DTL and CTGF. The above experiments demonstrate that the microarray data are accurate and can be used for future experiments.

### TXNIP is involved in NaBu‐ but not 4PBA‐induced cell death or caspase 3/7 activation

**Figure 3:**
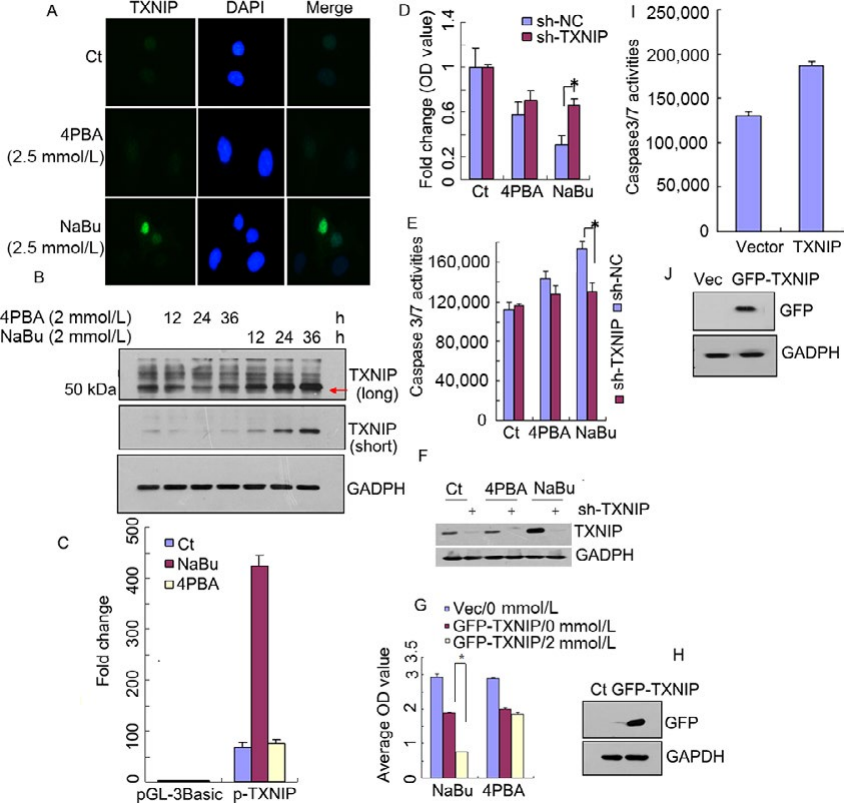
TXNIP is involved in NaBu‐induced cell death and caspase 3/7 activation. (A) A549 cells were treated with 2.5 mmol/L NaBu, 2.5 mmol/L 4PBA or vehicle (C_*t*_) for 12 h and then were used for immunofluorescence staining with anti‐TXNIP (Green); the cell nucleus was counterstained with DAPI (blue). (B) A549 cells were treated with 2 mmol/L NaBu or 4PBA for the designated durations and harvested for a western blot assay with anti‐TXNIP antibody. GAPDH was used as a loading control. Long, long exposure, short, short exposure. (C) TXNIP promoter was PCR‐amplified from A549 genomic DNA and inserted into pGL‐3Basic. The methods of analysis and data generation are the same as those in Figure 2D. (D, E, H) A549 cells stably expressing shTXNIP or shScramble (sh‐NC) were constructed. The above cells were treated with 5 mmol/L NaBu or 4PBA or vehicle (C_*t*_) for 24 h and then used to measure cell proliferation (MTT assay) (D) or caspase 3/7 activity (E). The shTXNIP knockdown efficiency was confirmed by a western blot (F). shTXNIP represents the TXNIP knockdown vector, shNC represents the scramble control; and *represents *P* < 0.05. (G–H) A549 cells were seeded on 6‐well cell culture plates 1 day before transfection and then transfected with empty vector or GFP‐tagged TXNIP vector. After 24 h of transfection, the cells were treated with 2 mmol/L NaBu or 4PBA or vehicle (0 mmol/L) for another 24 h then used for MTT assay (G), and TXNIP expression was confirmed by a western blot assay (H). (I–J) A549 cells were seeded on 6‐well cell culture plates 1 day before transfection and then transfected with empty vector or GFP‐tagged TXNIP vector via lipofectamine 3000 reagent. After 24 h of transfection, the cells were used for a caspase 3/7 activity assay (I) and a western blot assay (J). The results in all of the histograms are the means ± S.D. for three independent experiments. C_*t*_, control; 4PBA, sodium phenylbutyrate; NaBu, sodium butyrate; Vector, corresponding empty vector. The red arrow indicates TXNIP expression.

From the microarray data, we discovered that the transcription of the TXNIP gene in A549 cells was highly induced by NaBu treatment (>30‐fold) (Fig. S1). This finding prompted us to further investigate the role of TXNIP in NaBu‐induced A549 cell death. We first measured its protein level by cell immunostaining after 12 h of NaBu or 4PBA treatment. As shown Figure 3A, the TXNIP protein, which is localized to the nucleus, is obviously upregulated after NaBu treatment, but not 4PBA treatment (Fig. 3A). The western blot results further confirm the TXNIP expression trends under NaBu and 4PBA treatments (Fig. 3B). Then, we cloned the promoter fragment of the TXNIP gene into the pGL‐3Basic luciferase reporter vector and analyzed its promoter activities after NaBu or 4PBA treatment via the Dual‐Luciferase^®^ Reporter Assay System. The results show that the NaBu treatment caused a greater than 10‐fold increase in the relative luciferase activities; in contrast, 4PBA treatment only caused a slight increase (Fig. 3C). Because TXNIP is also involved in cell proliferation and apoptosis, we then measured the cell viability and caspase 3/7 activities under TXNIP overexpression or shRNA‐mediated TXNIP knockdown. The cell viability was determined by the MTT assay. Compared to negative control (C_*t*_) shNC cells, in TXNIP‐knockdown A549 cells, only the NaBu treatment induced significantly higher levels of cell viability (Fig. 3D) and lower levels of caspase 3/7 activity (Fig. 3E). The efficiency of TXNIP knockdown was assessed by western blot (Fig. 3H). As expected, TXNIP overexpression enhanced caspase 3/7 activity (Fig. 3G) and further decreased cell viability in response to NaBu (2 mmol/L), but not to 4PBA treatment (Fig. 3I). The TXNIP protein expression level was also confirmed by western blot (Fig. 3F and J). These results indicate that TXNIP might play a basal role in NaBu‐induced caspase 3/7 activation and cell death.

### Though both NaBu and 4PBA enhance the level of acH4K5, acH3K9, acH3K18, and H3K4me3 modification in A549 cells, TXNIP only modulates NaBu‐induced acH4K5 and H3K4me3 modifications

**Figure 4:**
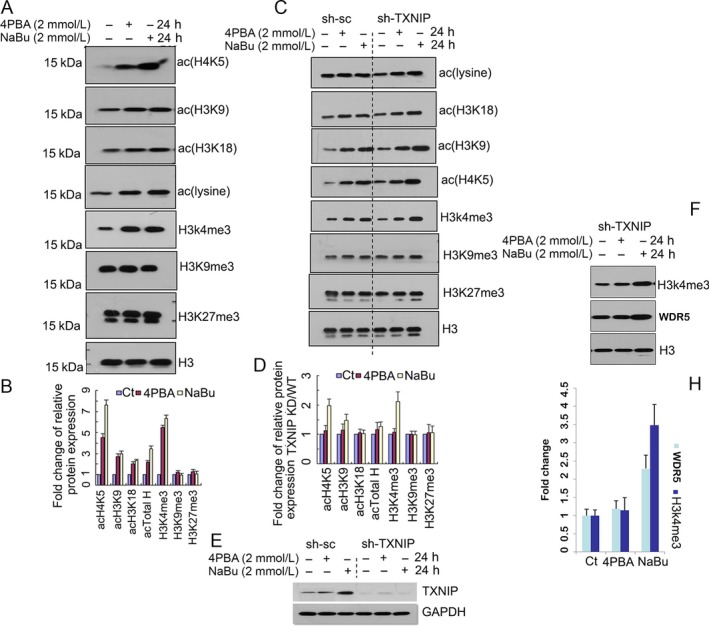
Though both NaBu and 4PBA enhance acH4K5, acH3K9, acH3K18 and H3K4me3 modification, TXNIP only modulates NaBu‐induced acH4K5 and H3K4me3 modification. (A, B) NaBu and 4PBA enhanced histone modification. A549 cells were treated with 2 mmol/L NaBu or 4PBA or vehicle for 24 h and then harvested for nuclear protein isolation. Western blotting was used to detect different types of histone modifications with the designated antibodies in the Figure. H3 was used as the loading control (A). Western blotting was performed three times, and the results are shown in gray. The relative intensity of each type of histone modification was normalized against the loading control H3 with an untreated control standardized to 1.0 (B). C, D: TXNIP knockdown promotes NaBu, but not 4PBA‐mediated acH4K5 and H3K4me3. A549 cells stably expressing shRNA‐mediated TXNIP knockdown and its shNC negative control (wild type) were treated with NaBu (2 mmol/L), 4PBA (2 mmol/L) or vehicle for 24 h. Then, the cells were used to detect histone modifications (C). Western blotting was performed three times, and the results are shown in gray. The relative intensity of each type of histone modification was normalized against the loading control H3 with an untreated control standardized to 1.0. The ratio of knockdown (KD)/negative control (WT) was used to determine the relative change of each type of histone modification (D). (E) The shTXNIP knockdown efficiency was confirmed by a western blot. (F–H) WDR5 and H3K4me3 expression was analyzed by a western blot assay in TXNIP‐knockdown cells (F), and the grey values were used to quantify the relative protein levels of WDR5 and H3K4me3 from three replicates of the western blot data (H).Histone H4 lysine 5 acetylation (acH4K5), H3 lysine 9 acetylation (acH3K9) and H3 lysine 18 acetylation(acH3K18), Histone H3 trimethylation lysine 4 (H3K4me3), Histone H3 trimethylation lysine 27 (H3K27me3) and Histone H3 trimethylation lysine 9 (H3K9me3). H3 was used for the reference control. The error bars represent the S.D. of three independent experiments. *represents *P* < 0.05.

Gene expression can be altered by remodeling the chromatin structure and by histone modification 3. As inhibitors of HDACs, NaBu and 4PBA are capable of changing histone modifications 7, 11. We therefore investigated the histone modifications in A549 cells under NaBu or 4PBA treatment. The results showed that both NaBu and 4PBA could enhance the total histone acetylation (upregulation more than threefold for NaBu and less than twofold for 4PBA), histone H4 lysine 5 acetylation (acH4K5) (upregulation close to ninefold for NaBu and fivefold for 4PBA), histone H3 lysine 9 acetylation (acH3K9) (upregulation close to threefold for both NaBu and 4PBA), histone H3 lysine 18 acetylation (acH3K18) (upregulation more than twofold for both NaBu and 4PBA) and histone H3 lysine 4 trimethylation (H3K4me3) (upregulation close to sevenfold for NaBu and sixfold for 4PBA), but histone H3 lysine 9 trimethylation (H3K9me3) and histone H3 lysine 27 trimethylation (H3K27me3) were not affected by treatment with either NaBu or 4PBA compared to the control (Fig. 4A and B). Because TXNIP expression is greatly upregulated in NaBu‐treated A549 cells and it also binds to HDAC1/3, we detected the histone modification in A549 cells stably expressing shTXNIP and scrambled shRNA (shNC) after treatment with NaBu or 4PBA for the designated time points and concentration. We found that acH4K5 (upregulation close to twofold) and H3K4me3 (upregulation more than twofold) were enhanced in TXNIP‐knockdown A549 cells when treated with NaBu, but not with 4PBA (Fig. 4C and D). Here, shRNA‐mediated TXNIP knockdown efficiency was confirmed by western blot (Fig. 4E). Because WDR5 is associated with H3K4me3 modification, we measured its expression after TXNIP knockdown. As shown in Figure 4F and H, the WDR5 protein expression increased more than twofold in TXNIP‐knockdown cells after NaBu treatment, but not after 4PBA treatment. These results indicate that TXNIP can mediate NaBu‐induced acH4K5 and H3K4me3.

### TXNIP mediates NaBu‐ but not 4PBA‐induced mitochondrial superoxide and cell death

**Figure 5:**
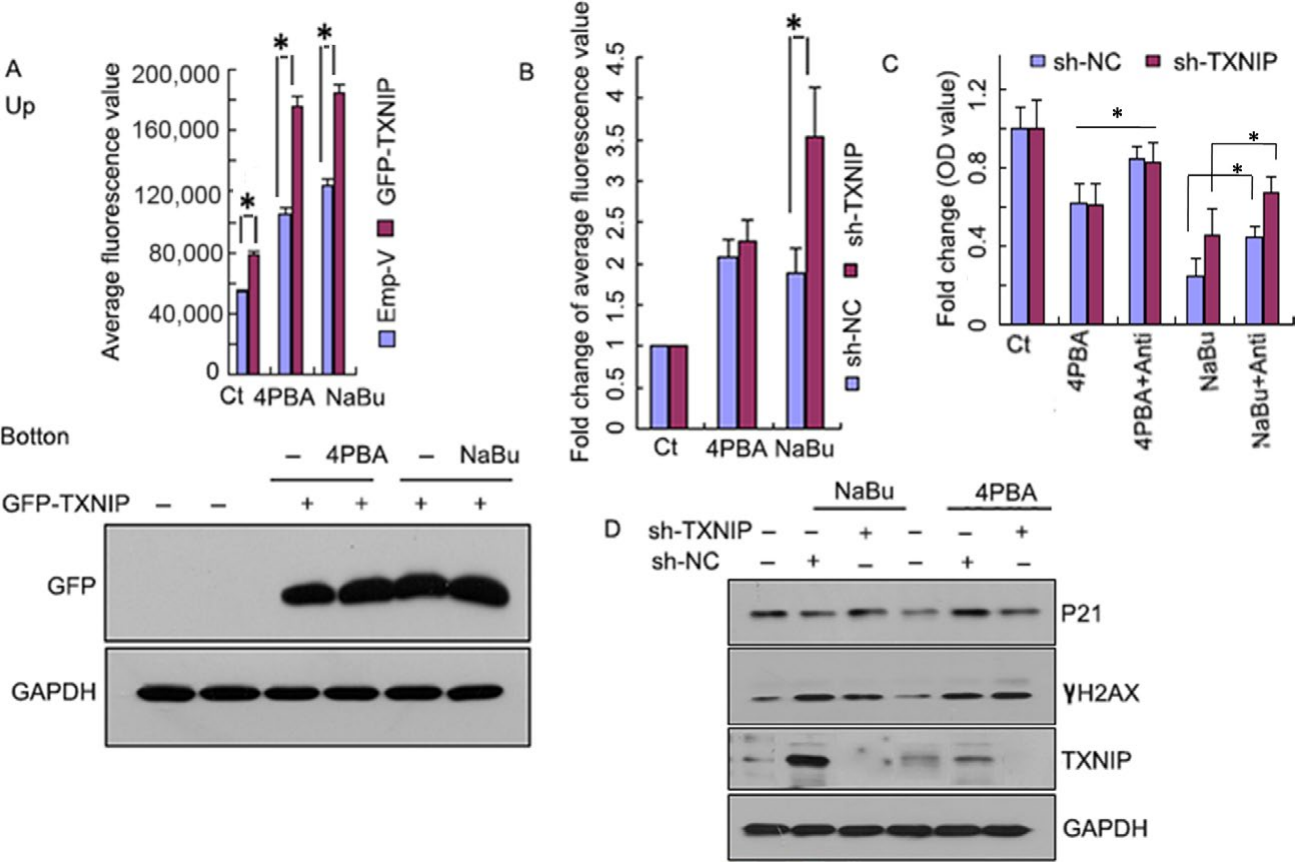
TXNIP mediates NaBu‐ but not 4PBA‐induced mitochondrial superoxide and cell death. (A) A549 cells were transfected with pEGFP‐C3 (Emp‐V) or pEGFP‐C3‐TXNIP (GFP‐TXNIP) with lipofectamine 3000. After 24 h of transfection, the above cells were pretreated with 5 mmol/L 4PBA or NaBu for another 24 h. The cells were then washed with fresh medium and incubated in medium containing MitoSOX Red (5 *μ*mol/L) and 4PBA or NaBu for a further 30 min at 37°C in the dark. The cells were washed with fresh serum‐free medium, and following washing, the fluorescence intensity of the cells was measured by flow cytometric analysis with wavelengths of Excitation/Emission of 510/580 nm. GFP‐TXNIP expression was detected by a western blot assay (bottom panel). (B) A549 cells stably expressing shTXNIP or shScramble (sh‐NC) were seeded on 6‐well culture plates before treatment with NaBu (5 mmol/L), 4PBA (5 mmol/L) or vehicle (C_*t*_). After 24 h of treatment, mitochondrial superoxide was measured as in A. (C) A549 cells stably expressing shTXNIP or shScramble (sh‐NC) were seeded on 6‐well culture plates before treatment with NaBu (5 mmol/L), 4PBA (5 mmol/L), vehicle (C_*t*_), NaBu+antimycin (Anti) (a complex inhibitor that inhibits mitochondrial superoxide generation) or 4PBA+Anti for 24 h. The cell viability was then analyzed by an MTT assay. (D) Western blotting was used to measure P21,*γ*H2AX and TXNIP expression in TXNIP‐knockdown (shTXNIP) or scramble (shNC) A549 cells after 24 h of treatment with 5 mmol/L NaBu or 4PBA. GAPDH was the loading control. The results in all of the histograms are the means ± S.D. for three independent experiments. C_*t*_, control; 4PBA, sodium phenylbutyrate; NaBu, sodium butyrate. *represents *P* < 0.05.

Acting as an intracellular second messenger, ROS mediates a variety of cellular events and causes numerous diseases, such as neurological disorders 7 and cancers 21, when it is disordered. Superoxide is the initial ROS and major byproduct of complex I and complex III of the electron transport chain during mitochondrial ATP synthesis 22. Here, we detected mitochondrial superoxide generation in A549 cells that had been transfected with the GFP‐TXNIP vector or its corresponding vector control under NaBu or 4PBA treatment, using the MitoSOX^™^ Red mitochondrial superoxide indicator for live‐cell imaging (MitoSOX Red) (M36008, Invitrogen) by FACS. The average fluorescence value increased to 80,000 after TXNIP overexpression compared to that of the vector control group, in which the average fluorescence value was <60,000 (Fig. 5A). NaBu treatment obviously increased the average fluorescence intensity for both cells expressing TXNIP (more than 180,000) or empty vector control cells (close to 120,000), and 4PBA treatment also increased the average fluorescence intensity by slightly more than 100,000 for the empty vector and close to 180,000 for TXNIP overexpression (Fig. 5A, top panel). The above results indicate that mitochondrial superoxide can be induced by NaBu, 4PBA, and TXNIP. TXNIP expression was confirmed by an immunoblot (Fig. 5A, bottom panel). On the other hand, a 3.5‐fold increase in the relative average fluorescence in A549 cells expressing shTXNIP was observed compared to that in those expressing shNC when treated with NaBu, but there was no observable change under 4PBA treatment (Fig. 5B). To determine whether mitochondrial superoxide mediates NaBu‐ or 4PBA‐induced cell death in TXNIP‐knockdown cells, we used the mitochondrial superoxide inhibitor antimycin, which inhibits complex III activity. The results show that antimycin treatment restored by almost 40% the cell viability in both shTXNIP and shNC cells under 4PBA treatment (Fig. 5C); however, the OD values of cells expressing shNC and shTXNIP were 0.25 and 0.45, respectively, indicating that TXNIP can sensitize the A549 cells to NaBu treatment. Antimycin treatment also restored the OD values to 0.44 and 0.67 for shNC and shTXNIP, respectively, indicating that mitochondrial superoxide plays a role in the induction of cell death caused by NaBu. While mitochondrial superoxide is partially involved in cell death induced by NaBu or 4PBA, other pathways might also contribute to this process. Therefore, we further observed the relationship between TXNIP knockdown and P21 expression or DNA damage under NaBu or 4PBA treatment. We found that under TXNIP knockdown, NaBu treatment increased P21 expression, but decreased the expression of γH2AX, a marker of DNA damage. Thus, TXNIP might exert its role in NaBu‐induced death by changing mitochondrial superoxide production, regulating P21 expression and the DNA damage response.

## Discussion

### Cell cycle‐associated genes are significantly downregulated in NaBu‐treated A549 cells

The anti‐tumor activity of NaBu and 4PBA has been previously reported in colorectal cancer cells and human prostate cancer cells 11. Here, we show that NaBu treatment (5 mmol/L) greatly induced A549 cell death after 72 h of treatment, which was significantly different from 4PBA treatment (5 mmol/L), although it also induced A549 cell death (Fig. 1A–C). These results suggest that NaBu or 4PBA might also be used for the treatment of non‐small‐cell lung cancer, the most common type of lung cancer and the leading cause of cancer‐related mortality worldwide 23. Gene expression changes are one of key characteristics for histone inhibitors 7, 11, 24. We therefore systematically investigated via a gene expression microarray the gene transcription changes in A549 cells undergoing NaBu or 4PBA treatment. We found that more than 100 cell cycle‐associated genes were significantly downregulated in NaBu‐treated cells,however, few cell cycle genes were slightly downregulated in 4PBA (Fig. 1 and Fig. S2). Although previous papers have also shown that NaBu downregulates cell cycle‐associated gene expression in other cell lines or species 25, 26, 27, the ranges of expression downregulation and the number of downregulated genes are relatively lower than ours. A possible explanation for that might contribute to the cell type‐dependent response to NaBu treatment. 4PBA‐treated cells only downregulated a small number of cell cycle genes, which is obviously different from the treatment with NaBu (Figs. 1, 2, S1 and S2). This difference possibly indicates that NaBu and 4PBA participate in cellular events through different molecular pathways. NaBu and 4PBA induce insulin‐like growth factor 2 (somatomedin A) (IGF2) 31‐fold and 48‐fold (Fig. S1 and S2), respectively. IGF2 is a mitogenic polypeptide that is abundantly expressed in the brain, especially in the cortex and hippocampus, and is required for memory consolidation and enhancement 28.

### 4PBA might have potential side effects during tumor treatment by inducing high level expression of oncogenes which are not or slightly induced under NaBu treatment

S100 calcium‐binding protein A9 (S100A9) was significantly upregulated by 4PBA (Fig. S2) and can promote breast cancer development by increasing migratory and invasive phenotypes and by forming a complex with S100A8, which plays a crucial role in psoriasis‐like skin disease and inflammation 29, 30. The proinflammatory chemokine interleukin 8 (IL‐8), which is involved in the chemoattraction and activation of neutrophils during the immune response and also has growth angiogenic and mitogenic effects in a variety of cancers, including prostate, ovarian, melanoma and carcinoma 31, 32, 33, 34, was induced more than sixfold by 4PBA, but less than twofold by NaBu (Fig. S1 and S2). Thymosin beta 15A (TMSB15A), a predictor of chemotherapy response in triple‐negative breast cancer, was significantly induced by sodium phenylbutyrate (Fig. S2) 35. Taken together, the induction of genes by 4PBA treatment might produce potential side effects during the treatment of the non‐small‐cell lung cancer.

### Enhancement of histone acetylation and trimethylation modification induced by NaBu or 4PBA is a marker of gene expression activation

As inhibitors of histone deacetylase, NaBu and 4PBA have been shown to enhance histone acetylation in many previously published papers. H4K5 acetylation is a marker of histone H4 hyperacetylation and a reliable predictor of actively transcribed genes 36, 37 and is also associated with embryo development 36, fear memory formation, and learning 37. Although previous research has reported that mice chronically treated with NaBu can restore the H4K5 acetylation which was inhibited by isoflurane administration 38; a direct observation has not been reported. In our study, both NaBu and 4PBA enhanced H4K5 acetylation; this is, to the best of our knowledge, the first such report (Fig. 4). H3K9 acetylation, which is also associated with gene transcriptional activation 39, 40, could be enhanced by NaBu or 4PBA treatment in A549 cells. In addition to gene transcriptional activation, H3K9 acetylation is also involved in the pluripotency of embryonic stem cells 39, 40 and UV‐induced DNA damage 41 and ameliorates the memory impairment that is induced by brain iron overload 42. The above information suggests that NaBu or 4PBA might participate in those events by controlling the H3K9 acetylation level of target genes. Histone trimethylation is associated with both gene activation and gene repression. Trimethylations on K4 of histone H3 (H3K4me3) are associated with gene activation, which is enriched around transcriptional start sites (TSSs). The trimethylations on K9 and K27 of histone H3 (H3K9me3 and H3K27me3, respectively), which are associated with gene silencing and heterochromatin structures 43, 44, were not affected by either NaBu or 4PBA treatment; however, the trimethylation modification of H3K4 was greatly upregulated (Fig. 4). Taken together, those results indicate that both 4PBA and NaBu play a role in gene transcriptional activation.

### TXNIP is an important regulator of the NaBu‐mediated signaling response

TXNIP, forming a complex with HDAC1 and HDAC3, binds and regulates target gene expression 18. Because HDAC1 and HDAC3 are associated with histone modification, we thus knocked down TXNIP. We found a significant increase in H4K5 acetylation and H3K4 trimethylation in cells undergoing NaBu, but not 4PBA treatment compared to those in the wild type (Fig. 4C and D). This result implies that NaBu‐induced H4K5 acetylation and H3K4 trimethylation are TXNIP dependent and TXNIP might exert its role by regulating HDAC activity. WDR5 is involved in H3K4 trimethylation modification; we further found that TXNIP knockdown promotes WDR5 expression after NaBu treatment, but not after 4PBA treatment. This result might suggest a novel pathway controlling NaBu‐enhanced cell H3K4 trimethylation. We also observed that under TXNIP overexpression, NaBu‐ and 4PBA‐treated A549 cells could significantly upregulate mitochondrial superoxide generation (Fig. 5A). These results are consistent with those of previous studies 45 but contradict the finding that 4PBA inhibits the production of ROS from activated microglia 46. This difference suggests that 4PBA‐induced ROS generation might be a cell type or concentration dependent. Because TXNIP is a regulator of ROS generation, inflammasome activation and oxidative stress 47, 48, the enhancement of mitochondrial superoxide generation in A549 cells under TXNIP overexpression is expected (Fig. 5B); however, there was a significant enhancement of mitochondrial superoxide in cells with TXNIP knockdown under NaBu treatment, but not under 4PBA treatment (Fig. 5C). A similar result has been reported in previous studies, in which TXNIP deficiency induced H_2_O_2_ and superoxide during LPS‐induced inflammation in macrophages 49. Based on the above observation, we argue that TXNIP might negatively regulate mitochondrial superoxide generation. It will be interesting to further investigate the association between the basal level of TXNIP and mitochondrial superoxide generation. 4PBA‐mediated cell death and mitochondrial superoxide generation can be restored by antimycin treatment, indicating that mitochondrial superoxide is the main contributor to A549 cell death. We found that caspase 3/7 activation could be regulated by modulating TXNIP expression and mediating NaBu‐induced A549 cell death (Fig. 3). These results indicate that NaBu induced A549 cell death in a TXNIP‐dependent manner; in contrast, the 4PBA could not significantly induce caspase 3/7 activation after TXNIP knockdown, suggesting that it acts in a TXNIP‐independent manner. TXNIP‐mediated NaBu‐induced cell death may also occur by increased P21 expression and increased DNA damage response (Fig. 5D)

Together, we identified TXNIP as an important regulator of NaBu‐mediated cell death and other investigated responses in comparison with 4PBA.

## Conflict of Interest

The authors declare no conflict of interest.

## Supporting information


**Figure S1**. NaBu induces gene changes of more than 4‐fold.
**Figure S2**. 4PBA induces gene changes of more than 4‐fold.

## References

[1] Lachner, M. , R. J. Sullivan , and T. Jenuwein . 2003 An epigenetic road map for histone lysine methylation. J. Cell Sci. 116:2117–2124. 12730288 10.1242/jcs.00493

[2] Lachner, M. , and T. Jenuwein . 2002 The many faces of histone lysine methylation. Curr. Opin. Cell Biol. 14:286–298. 12067650 10.1016/s0955-0674(02)00335-6

[3] Greer, E. L. , and Y. Shi . 2012 Histone methylation: a dynamic mark in health, disease and inheritance. Nat. Rev. Genet. 13:343–357. 22473383 10.1038/nrg3173 PMC4073795

[4] Vallianatos, C. N. , and S. Iwase . 2015 Disrupted intricacy of histone H3K4 methylation in neurodevelopmental disorders. Epigenomics 7:501–517. 10.2217/epi.15.1 PMC4501478 26077434

[5] Giri, S. , V. Aggarwal , J. Pontis , A. Chakraborty , A. Khan , C. Mizzen et al. 2015 The preRC protein ORCA organizes heterochromatin by assembling histone H3 lysine 9 methyltransferases on chromatin. Elife 4:1–30. 10.7554/eLife.06496 PMC4442312 25922909

[6] Chen, X. , W. B. Xie , P. Gu , Q. Cai , B. Wang , Y. Xie , et al. Upregulated WDR5 promotes proliferation, self‐renewal and chemoresistance in bladder cancer via mediating H3K4 trimethylation. Sci. Rep 8293:1–12. 10.1038/srep08293 PMC4319178 25656485

[7] Donohoe, D. R. , L. B. Collins , A. Wali , R. Bigler , W. Sun , and S. J. Bultman . 2012 The Warburg Effect Dictates the Mechanism of Butyrate Mediated Histone Acetylation and Cell Proliferation. Mol Cell. 48:612–626. 23063526 10.1016/j.molcel.2012.08.033 PMC3513569

[8] Jeng, J. H. , M. Y. Kuo , P. H. Lee , Y. J. Wang , M. Y. Lee , J. J. Lee , et al. 2006 Toxic and metabolic effect of sodium butyrate on SAS tongue cancer cells: role of cell cycle deregulation and redox changes. Toxicology. 223:235–247. 16737765 10.1016/j.tox.2006.04.033

[9] Villar‐Garea, A. , and M. Esteller . 2004 Histone deacetylase inhibitors: Understanding a new wave of anticancer agents. Int. J. Cancer. 2004:171–178. 10.1002/ijc.20372 15352027

[10] Rada‐Iglesias, A. , S. Enroth , A. Ameur , C. M. Koch , G. K. Clelland , P. Respuela‐Alonso , et al. 2007 Butyrate mediates decrease of histone acetylation centered on transcription start sites and down‐regulation of associated genes. Genome Res. 17:708–719. 17567991 10.1101/gr.5540007 PMC1891332

[11] Iannitti, T. , and B. Palmieri . 2011 Clinical and Experimental Applications of Sodium Phenylbutyrate. Drugs 11:227–249. 10.2165/11591280-000000000-00000 PMC3586072 21902286

[12] Dovzhanskiy, D. I. , W. Hartwig , N. G. Lázár , A. Schmidt , K. Felix , B. K. Straub , et al. 2012 Growth inhibition of pancreatic cancer by experimental treatment with 4‐phenylbutyrate is associated with increased expression of Connexin 43. Oncol. Res. 20:103–111. 23193916 10.3727/096504012x13477145152959

[13] Hayashi, H. , T. Mizuno , R. Horikawa , H. Nagasak , T. Yabuki , H. Takikawa , et al. 2012 4‐Phenylbutyrate modulates ubiquitination of hepatocanalicular MRP2 and reduces serum total bilirubin concentration. J. Hepatol. 56:1136–1144. 22245901 10.1016/j.jhep.2011.11.021

[14] Cha‐Molstad, H. , G. Saxena , J. Chen , and A. Shalev . 2009 Glucose‐stimulated expression of Txnip is mediated by carbohydrate response element‐binding protein, p300, and histone H4 acetylation in pancreatic beta cells. J. Biol. Chem. 284:16898–16905. 19411249 10.1074/jbc.M109.010504 PMC2719326

[15] Zhang, P. Z. , C. J. Wang , K. Gao , D. Wang , J. Mao , J. An , et al. 2010 The ubiquitin ligase itch regulates apoptosis by targeting thioredoxin‐interacting protein for ubiquitin‐dependent degradation. J. Biol. Chem. 285:8869–8879. 20068034 10.1074/jbc.M109.063321 PMC2838308

[16] Baker, A. F. , M. Y. Koh , R. R. Williams , B. James , H. Wang , W. R. Tate , et al. 2008 Identification of thioredoxin‐interacting protein 1 as a hypoxia‐inducible factor 1 alpha‐induced gene in pancreatic cancer. Pancreas 36:178–186. 18376310 10.1097/MPA.0b013e31815929fe

[17] Wu, N. , B. Zheng , A. Shaywitz , Y. Dagon , C. Tower , G. Bellinger , et al. 2013 AMPK‐dependent degradation of TXNIP upon energy stress leads to enhanced glucose uptake via GLUT1. Mol Cell. 49:1167–1175. 23453806 10.1016/j.molcel.2013.01.035 PMC3615143

[18] Lee, S. , S. M. Kim , and R. T. Lee . 2013 Thioredoxin and thioredoxin target proteins: from molecular mechanisms to functional significance. Antiox. Redox Signal. 18:1165–1207. 10.1089/ars.2011.4322 PMC3579385 22607099

[19] Archer, S. Y. , S. F. Meng , A. Shei , and R. A. Hodin . 1998 p21WAF1 is required for butyrate‐mediated growth inhibition of human colon cancer cells. Proc. Natl Acad. Sci. 95:6791–6796. 9618491 10.1073/pnas.95.12.6791 PMC22637

[20] Chopin, V. , R. A. Toillon , N. Jouy , and X. Le Bourhis . 2002 Sodium butyrate induces P53‐independent, Fas‐mediated apoptosis in MCF‐7 human breast cancer cells. Br. J. Pharmacol. 135:79–86. 11786482 10.1038/sj.bjp.0704456 PMC1573118

[21] Patel, M. 2016 Targeting Oxidative Stress in Central Nervous System Disorders. Trends Pharmacol Sci. 37:768–78. 27491897 10.1016/j.tips.2016.06.007 PMC5333771

[22] Waris, G. , and H. Ahsan . 2006 Reactive oxygen species: role in the development of cancer and various chronic conditions. J. Carcinogen. 5:14–21. 10.1186/1477-3163-5-14 PMC1479806 16689993

[23] Gius, D. , and D. R. Spitz . 2006 Redox signaling in cancer biology. Antioxid. Redox Signal. 8:1249–1252. 16910772 10.1089/ars.2006.8.1249

[24] Holley, A. K. , S. K. Dhar , Y. Xu , and D. K. St Clair . 2012 Manganese superoxide dismutase: beyond life and death. Amino Acids 42:139–158. 20454814 10.1007/s00726-010-0600-9 PMC2975048

[25] Mi, Y. J. , G. J. Geng , Z. Z. Zou , J. Gao , X. Y. Luo , Y. Liu , et al. 2015 Dihydroartemisinin inhibits glucose uptake and cooperates with glycolysis inhibitor to induce apoptosis in non‐small cell lung carcinoma cells. PLoS ONE 10:1–21. 10.1371/journal.pone.0120426 PMC4370589 25799586

[26] Zhou, Q. , C. L. Dalgard , C. Wynder , and M. l. Doughty . 2011 Histone deacetylase inhibitors SAHA and sodium butyrate block G1‐to‐S cell cycle progression in neurosphere formation by adult subventricular cells. BMC Neurosci.. doi:10.1186/1471‐2202‐12‐50. 10.1186/1471-2202-12-50 PMC3123242 21615950

[27] Chen, H. M. , Y. W. Lin , J. L. Wang , X. Kong , J. Hong , J. Y. Fang . 2013 Identification of potential target genes of butyrate in dimethylhydrazine‐induced colorectal cancer in mice. Nutr. Cancer 65:1171–1183. 24099273 10.1080/01635581.2013.828087

[28] Li, R. W. , and C. J. Li . 2006 Butyrate induces profound changes in gene expression related to multiple signal pathways in bovine kidney epithelial cells. BMC Genom. 7:234. 10.1186/1471-2164-7-234 PMC1592091 16972989

[29] Putaala, H. , H. Mäkivuokko , K. Tiihonen , and N. Rautonen . 2011 Simulated colon fiber metabolome regulates genes involved in cell cycle, apoptosis, and energy metabolism in human colon cancer cells. Mol. Cell. Biochem. 357(1–2):235–245. 21667160 10.1007/s11010-011-0894-2

[30] Wagner, G. F. , M. Hampong , C. M. Park , D. H. Copp . 1986 Purification, characterization, and bioassay of teleocalcin, a glycoprotein from salmon corpuscles of Stannius. Gen. Comp. Endocrinol. 63:481–491. 3557071 10.1016/0016-6480(86)90149-8

[31] Yeung, B. H. , A. Y. Law , and C. K. Wong . 2012 Evolution and roles of stanniocalcin. Mol. Cell. Endocrinol. 349:272–280. 22115958 10.1016/j.mce.2011.11.007

[32] Xie, K. 2001 Interleukin‐8 and human cancer biology. Cytokine Growth Factor Rev. 12:375–391. 11544106 10.1016/s1359-6101(01)00016-8

[33] Araki, S. , Y. Omori , D. Lyn , R. K. Singh , D. M. Meinbach , Y. Sandman , et al. 2007 Interleukin‐8 is a molecular determinant of androgen independence and progression in prostate cancer. Cancer Res. 67:6854–6862. 17638896 10.1158/0008-5472.CAN-07-1162

[34] Gabellini, C. , D. Trisciuoglio , M. Desideri , A. Candiloro , Y. Ragazzoni , A. Orlandi , et al. 2009 Functional activity of CXCL8 receptors, CXCR1 and CXCR2, on human malignant melanoma progression. Eur. J. Cancer 45:2618–2627. 19683430 10.1016/j.ejca.2009.07.007

[35] Wilson, C. , T. Wilson , P. G. Johnston , D. B. Longley , and D. J. Waugh . 2008 Interleukin‐8 signaling attenuates TRAIL‐ and chemotherapy‐induced apoptosis through transcriptional regulation of c‐FLIP in prostate cancer cells. Mol. Cancer Ther. 7:2649–2661. 18790747 10.1158/1535-7163.MCT-08-0148

[36] Alberini, C. M. , D. Y. Chen . 2012 Memory enhancement: consolidation, reconsolidation and insulin‐like growth factor 2. Trends Neurosci. 35:274–283. 22341662 10.1016/j.tins.2011.12.007 PMC3348400

[37] Chen, C. H. , J. Xu , W. F. Chang , C. C. Liu , H. Y. Su , Y. E. Chen , et al. 2012 Dynamic profiles of Oct‐4, Cdx‐2 and acetylated H4K5 in in‐vivo‐derived rabbit embryos. Reprod. Biomed. Online 25:358–370. 22877942 10.1016/j.rbmo.2012.07.001 PMC3465499

[38] Park, C. S. , H. Rehrauer , and I. M. Mansuy . 2013 Genome‐wide analysis of H4K5 acetylation associated with fear memory in mice. BMC Genom. 14:539. 10.1186/1471-2164-14-539 PMC3751108 23927422

[39] Zhong, T. , Q. J. Qing , Y. Yang , W. Y Zou , Z. Ye , J. Q. Yan , et al. 2014 Repression of contextual fear memory induced by isoflurane is accompanied by reduction in histone acetylation and rescued by sodium butyrate. Br. J. Anaesth. 113:634–643. 24838805 10.1093/bja/aeu184

[40] Hezroni, H. , B. S. Sailaja , and E. Meshorer . 2011a Pluripotency‐related, Valproic Acid (VPA)‐induced Genome‐wide Histone H3 Lysine 9 (H3K9) Acetylation Patterns in Embryonic Stem Cells. Biol. Chem. 286:35977–35988. 10.1074/jbc.M111.266254 PMC3195619 21849501

[41] Hezroni, H. , I. Tzchori , A. Davidi , A. Mattout , A. Biran , M. Nissim‐Rafinia , et al. 2011b H3K9 histone acetylation predicts pluripotency and reprogramming capacity of ES cells. Nucleus 2:300–309. 21941115 10.4161/nucl.2.4.16767 PMC3260568

[42] Guo, R. F. , J. Chen , D. L. Mitchell , and D. G. Johnson . 2011 GCN5 and E2F1 stimulate nucleotide excision repair by promoting H3K9 acetylation at sites of damage. Nucleic Acids Res. 39:1390–1397. 20972224 10.1093/nar/gkq983 PMC3045616

[43] Silva, P. F. , V. A. Garcia , S. Dornelles Ada , V. K. Silva , N. Maurmann , B. C. Portal , et al. 2012 Memory impairment induced by brain iron overload is accompanied by reduced H3K9 acetylation and ameliorated by sodium butyrate. Neuroscience 200:42–49. 22067609 10.1016/j.neuroscience.2011.10.038

[44] Kouzarides, T. 2007 Chromatin modifications and their function. Cell 128:693–705. 17320507 10.1016/j.cell.2007.02.005

[45] Riclet, R. , M. Chendeb , J. L. Vonesch , D. Koczan , H. J. Thiesen , R. Losson , et al. 2009 Disruption of the Interaction between transcriptional Intermediary factor 1β and heterochromatin protein 1 leads to a switch from DNA Hyper‐ to hypomethylation and H3K9 to H3K27 trimethylation on the MEST promoter correlating with gene reactivation. Mol. Biol. Cell 20:296–305. 18923144 10.1091/mbc.E08-05-0510 PMC2613122

[46] Butler, L. M. , X. Zhou , W. S. Xu , H. I. Scher , R. A. Rifkind , P. A. Marks , et al. 2002 The histone deacetylase inhibitor SAHA arrests cancer cell growth, up‐regulates thioredoxin‐binding protein‐2, and down‐regulates thioredoxin. Proc Natl Acad Sci. 99:11700–11705. 12189205 10.1073/pnas.182372299 PMC129332

[47] Cueno, M. E. , K. Imai , N. Matsukawa , T. Tsukahara , T. Kurita‐Ochiai , K. Ochiai , et al. 2013 Butyric acid retention in gingival tissue induces oxidative stress in jugular blood mitochondria. Cell Stress Chaperon. 18:661–665. 10.1007/s12192-013-0409-z PMC3745256 23397230

[48] Roy, A. , A. Ghosh , A. Jana , S. Brahmachari , H. E. Gendelman , K. Pahan , et al. 2012 Sodium phenylbutyrate controls neuroinflammatory and antioxidant activities and protects dopaminergic neurons in mouse models of parkinson's disease. PLoS ONE 7:1–18. 10.1371/journal.pone.0038113 PMC3377667 22723850

[49] Jung, H. Y. , M. J. Kim , D. O. Kim , W. S. Kim , S. J. Yoon , Y. J. Park , et al. 2013 TXNIP maintains the hematopoietic cell pool by switching the function of p53 under oxidative stress. Cell Metab. 18:75–85. 23823478 10.1016/j.cmet.2013.06.002

[50] Zhang, X. , J. H. Zhang , X. Y. Chen , Q. H. Hu , M. X. Wang , R. Jin , et al. 2015 Reactive oxygen species‐induced TXNIP drives fructose‐mediated hepatic inflammation and lipid accumulation through NLRP3 inflammasome activation. Antioxid. Redox Signal. 22:848–870. 25602171 10.1089/ars.2014.5868 PMC4367240

[51] Park, Y. J. , S. J. Yoon , H. W. Suh , D. O. Kim , J. R. Park , H. Jung , et al. 2013 TXNIP deficieny exacerbates endotoxic shock via the induction of excessive nitric oxide synthesis. PLoS Pathog. 9:e1003646. 24098117 10.1371/journal.ppat.1003646 PMC3789754

