# Supplementary material for: TXNIP mediates the differential responses of A549 cells to sodium butyrate and sodium 4‐phenylbutyrate treatment
Source: Cancer Med. 2016 Dec 29;6(2):424–38. doi: 10.1002/cam4.977 (PMC5313639; doi:10.1002/cam4.977)
Supplement: Supplementary file 1 — Figure S1. NaBu induces gene changes of more than 4‐fold. Figure S2. 4PBA induces gene changes of more than 4‐fold. [file CAM4-6-424-s001.docx]

**Supporting information**

Figure S1 NaBu induces gene changes of more than 4-fold.

| Column ID | Entrez Gene | Gene Symbol | Gene Title | RefSeq Transcript ID | Fold-Chang  e(NaBu/Ct) | Fold-Chang  e(NaBu/Ct) | Fold-Chang  e(4PBA/Ct) | Fold-Change  (4PBA/Ct) | SS(Type) |
| --- | --- | --- | --- | --- | --- | --- | --- | --- | --- |
| 11715440_a_at | 1490 | CTGF | connective tissue growth factor | NM_001901 | 23.5434 | NaBu/Ct | -1.16912 | 4PBA/Ct | 23.0434 |
| 11715441_a_at | 1490 | CTGF | connective tissue growth factor | NM_001901 | 24.7395 | NaBu/Ct | -1.61074 | 4PBA/Ct | 26.288 |
| 11715442_s_at | 1490 | CTGF | connective tissue growth factor | NM_001901 | 14.6591 | NaBu/Ct | -1.71491 | 4PBA/Ct | 19.1558 |
| 11715493_a_at | 3491 | CYR61 | cysteine-rich, angiogenic inducer, 61 | NM_001554 | 6.81204 | NaBu/Ct | -3.02755 | 4PBA/Ct | 13.8089 |
| 11715847_x_at | 5327 | PLAT | plasminogen activator, tissue | NM_000930 | 8.25113 | NaBu/Ct | 2.32287 | 4PBA/Ct | 6.27238 |
| 11716034_a_at | 684 | BST2 | bone marrow stromal cell antigen 2 | NM_004335 | 15.9386 | NaBu/Ct | 2.70591 | 4PBA/Ct | 9.98475 |
| 11716035_at | 684 | BST2 | bone marrow stromal cell antigen 2 | NM_004335 | 9.67903 | NaBu/Ct | 2.40251 | 4PBA/Ct | 6.37685 |
| 11716036_x_at | 684 | BST2 | bone marrow stromal cell antigen 2 | NM_004335 | 11.4219 | NaBu/Ct | 2.19663 | 4PBA/Ct | 7.87585 |
| 11716092_x_at | 1163 | CKS1B | CDC28 protein kinase regulatory subunit 1B | NM_001826 | -8.56004 | NaBu/Ct | -2.18089 | 4PBA/Ct | 6.59134 |
| 11716210_at | 10370 | CITED2 | Cbp/p300-interacting transactivator, with Glu/Asp-rich carboxy-terminal domain, 2 | NM_001168388 | 8.67352 | NaBu/Ct | 1.11652 | 4PBA/Ct | 8.76338 |
| 11716459_at | 7277 | TUBA4A | tubulin, alpha 4a | NM_006000 | -5.79318 | NaBu/Ct | -2.10401 | 4PBA/Ct | 3.87733 |
| 11716460_x_at | 7277 | TUBA4A | tubulin, alpha 4a | NM_006000 | -5.95136 | NaBu/Ct | -2.31036 | 4PBA/Ct | 3.92293 |
| 11716641_x_at | 5699 | PSMB10 | proteasome (prosome, macropain) subunit, beta type, 10 | NM_002801 | -5.10022 | NaBu/Ct | -1.78977 | 4PBA/Ct | 3.54908 |
| 11716642_s_at | 1841 | DTYMK | deoxythymidylate kinase (thymidylate kinase) | NM_001165031 | -6.66193 | NaBu/Ct | -2.59033 | 4PBA/Ct | 4.53446 |
| 11716697_at | 2167 | FABP4 | fatty acid binding protein 4, adipocyte | NM_001442 | 9.69065 | NaBu/Ct | -1.31382 | 4PBA/Ct | 13.746 |
| 11716791_a_at | 5954 | RCN1 | reticulocalbin 1, EF-hand calcium binding domain | NM_002901 | -6.60948 | NaBu/Ct | 1.05613 | 4PBA/Ct | 7.55927 |
| 11716792_s_at | 5954 | RCN1 | reticulocalbin 1, EF-hand calcium binding domain | NM_002901 | -7.05483 | NaBu/Ct | 1.17617 | 4PBA/Ct | 8.34424 |
| 11716793_a_at | 9133 | CCNB2 | cyclin B2 | NM_004701 | -5.55977 | NaBu/Ct | -1.91841 | 4PBA/Ct | 3.72457 |
| 11716895_s_at | 9636 | ISG15 | ISG15 ubiquitin-like modifier | NM_005101 | 8.26257 | NaBu/Ct | 1.27068 | 4PBA/Ct | 9.03206 |
| 11717025_s_at | 6374 | CXCL5 | chemokine (C-X-C motif) ligand 5 | NM_002994 | -4.29327 | NaBu/Ct | 1.02348 | 4PBA/Ct | 5.29639 |
| 11717062_at | 10053 | AP1M2 | adaptor-related protein complex 1, mu 2 subunit | NM_005498 | -8.85384 | NaBu/Ct | -3.19111 | 4PBA/Ct | 7.16012 |
| 11717090_a_at | 8349 | HIST2H2BE | histone cluster 2, H2be | NM_003528 | 5.65376 | NaBu/Ct | -1.51617 | 4PBA/Ct | 7.9834 |
| 11717163_s_at | 991 | CDC20 | cell division cycle 20 homolog (S. cerevisiae) | NM_001255 | -4.61793 | NaBu/Ct | -3.04521 | 4PBA/Ct | 2.9943 |
| 11717190_s_at | 10628 | TXNIP | thioredoxin interacting protein | NM_006472 | 58.836 | NaBu/Ct | 3.17339 | 4PBA/Ct | 26.1998 |
| 11717213_a_at | 8439 | NSMAF | neutral sphingomyelinase (N-SMase) activation associated factor | NM_001144772 | -6.46506 | NaBu/Ct | -1.59866 | 4PBA/Ct | 7.36133 |
| 11717214_s_at | 8439 | NSMAF | neutral sphingomyelinase (N-SMase) activation associated factor | NM_001144772 | -6.86152 | NaBu/Ct | -1.41254 | 4PBA/Ct | 8.05671 |
| 11717284_a_at | 8976 | WASL | Wiskott-Aldrich syndrome-like | NM_003941 | 5.1786 | NaBu/Ct | 2.53924 | 4PBA/Ct | 3.03375 |
| 11717355_a_at | 9638 | FEZ1 | fasciculation and elongation protein zeta 1 (zygin I) | NM_005103 | 8.69579 | NaBu/Ct | 6.80514 | 4PBA/Ct | 6.8809 |
| 11717473_s_at | 3484 | IGFBP1 | insulin-like growth factor binding protein 1 | NM_000596 | -53.2546 | NaBu/Ct | -2.01533 | 4PBA/Ct | 27.0414 |
| 11717589_a_at | 11340 | EXOSC8 | exosome component 8 | NM_181503 | -4.84516 | NaBu/Ct | -2.02808 | 4PBA/Ct | 3.43419 |
| 11717672_s_at | 5168 | ENPP2 | ectonucleotide pyrophosphatase/phosphodiesterase 2 | NM_001040092 | 4.10883 | NaBu/Ct | 1.30628 | 4PBA/Ct | 3.00884 |
| 11718058_a_at | 7298 | TYMS | thymidylate synthetase | NM_001071 | -20.0262 | NaBu/Ct | -3.10061 | 4PBA/Ct | 13.3887 |
| 11718183_at | 113130 | CDCA5 | cell division cycle associated 5 | NM_080668 | -17.0999 | NaBu/Ct | -3.54077 | 4PBA/Ct | 9.79511 |
| 11718230_a_at | 3134 | HLA-F | major histocompatibility complex, class I, F | NM_001098478 | 8.00024 | NaBu/Ct | 6.40029 | 4PBA/Ct | 6.44431 |
| 11718231_x_at | 3134 | HLA-F | major histocompatibility complex, class I, F | NM_001098478 | 5.41171 | NaBu/Ct | 5.35555 | 4PBA/Ct | 4.85213 |
| 11718403_at | 5983 | RFC3 | replication factor C (activator 1) 3, 38kDa | NM_002915 | -7.89308 | NaBu/Ct | -2.73658 | 4PBA/Ct | 5.68443 |
| 11718404_at | 5983 | RFC3 | replication factor C (activator 1) 3, 38kDa | NM_002915 | -8.01603 | NaBu/Ct | -3.21552 | 4PBA/Ct | 5.66828 |
| 11718588_a_at | 56271 | BEX4 | brain expressed, X-linked 4 | NM_001080425 | 13.3923 | NaBu/Ct | 9.38971 | 4PBA/Ct | 9.47742 |
| 11718926_a_at | 84159 | ARID5B | AT rich interactive domain 5B (MRF1-like) | NM_032199 | 4.06928 | NaBu/Ct | 2.58382 | 4PBA/Ct | 2.49316 |
| 11718927_a_at | 84159 | ARID5B | AT rich interactive domain 5B (MRF1-like) | NM_032199 | 10.6513 | NaBu/Ct | 6.86224 | 4PBA/Ct | 7.8079 |
| 11718932_a_at | 27111 | SDCBP2 | syndecan binding protein (syntenin) 2 | NM_015685 | -3.72429 | NaBu/Ct | 1.15406 | 4PBA/Ct | 3.71933 |
| 11718933_s_at | 27111 | SDCBP2 | syndecan binding protein (syntenin) 2 | NM_015685 | -19.8452 | NaBu/Ct | 1.12315 | 4PBA/Ct | 18.2382 |
| 11718943_a_at | 6790 | AURKA | aurora kinase A | NM_003600 | -13.1309 | NaBu/Ct | -2.80148 | 4PBA/Ct | 8.69876 |
| 11718986_a_at | 2537 | IFI6 | interferon, alpha-inducible protein 6 | NM_002038 | 5.93855 | NaBu/Ct | 1.38434 | 4PBA/Ct | 4.98624 |
| 11718998_x_at | 1719 | DHFR | dihydrofolate reductase | NM_000791 | -7.38581 | NaBu/Ct | -3.25362 | 4PBA/Ct | 5.92558 |
| 11718999_x_at | 1719 | DHFR | dihydrofolate reductase | NM_000791 | -6.39585 | NaBu/Ct | -3.91867 | 4PBA/Ct | 4.99489 |
| 11719000_x_at | 1719 | DHFR | dihydrofolate reductase | NM_000791 | -6.63271 | NaBu/Ct | -3.23239 | 4PBA/Ct | 6.0037 |
| 11719265_a_at | 79083 | MLPH | melanophilin | NM_001042467 | -4.90409 | NaBu/Ct | 1.01636 | 4PBA/Ct | 6.53733 |
| 11719323_at | 5728 | PTEN | phosphatase and tensin homolog | NM_000314 | 5.46223 | NaBu/Ct | 1.37812 | 4PBA/Ct | 5.64767 |
| 11719324_x_at | 5728 | PTEN | phosphatase and tensin homolog | NM_000314 | 5.06705 | NaBu/Ct | 1.13581 | 4PBA/Ct | 5.40405 |
| 11719382_a_at | 10465 | PPIH | peptidylprolyl isomerase H (cyclophilin H) | NM_006347 | -5.28117 | NaBu/Ct | -2.22823 | 4PBA/Ct | 3.4162 |
| 11719383_x_at | 10465 | PPIH | peptidylprolyl isomerase H (cyclophilin H) | NM_006347 | -5.12169 | NaBu/Ct | -2.40198 | 4PBA/Ct | 3.30113 |
| 11719598_s_at | 83461 | CDCA3 | cell division cycle associated 3 | NM_031299 | -9.53065 | NaBu/Ct | -3.51392 | 4PBA/Ct | 7.2881 |
| 11719699_a_at | 8318 | CDC45 | cell division cycle 45 homolog (S. cerevisiae) | NM_001178010 | -12.3371 | NaBu/Ct | -5.16226 | 4PBA/Ct | 8.26672 |
| 11719760_at | 91947 | ARRDC4 | arrestin domain containing 4 | NM_183376 | 7.01586 | NaBu/Ct | 1.61514 | 4PBA/Ct | 6.45005 |
| 11719761_at | 91947 | ARRDC4 | arrestin domain containing 4 | NM_183376 | 22.9828 | NaBu/Ct | 1.81561 | 4PBA/Ct | 17.0793 |
| 11719797_a_at | 8623 | ASMTL | acetylserotonin O-methyltransferase-like | NM_001173473 | 7.27997 | NaBu/Ct | 2.37045 | 4PBA/Ct | 5.6088 |
| 11720141_a_at | 3161 | HMMR | hyaluronan-mediated motility receptor (RHAMM) | NM_001142556 | -5.67365 | NaBu/Ct | -1.77813 | 4PBA/Ct | 5.68178 |
| 11720185_a_at | 4085 | MAD2L1 | MAD2 mitotic arrest deficient-like 1 (yeast) | NM_002358 | -9.73919 | NaBu/Ct | -3.11509 | 4PBA/Ct | 7.75654 |
| 11720186_s_at | 4085 | MAD2L1 | MAD2 mitotic arrest deficient-like 1 (yeast) | NM_002358 | -9.88458 | NaBu/Ct | -4.89493 | 4PBA/Ct | 7.42819 |
| 11720203_a_at | 9212 | AURKB | aurora kinase B | NM_004217 | -7.17759 | NaBu/Ct | -2.21483 | 4PBA/Ct | 4.88906 |
| 11720240_at | 11013 | TMSB15A | thymosin beta 15a | NM_021992 | 8.52466 | NaBu/Ct | 9.86131 | 4PBA/Ct | 7.19831 |
| 11720241_at | 24137 | KIF4A | kinesin family member 4A | NM_012310 | -5.76658 | NaBu/Ct | -2.30862 | 4PBA/Ct | 4.15037 |
| 11720255_a_at | 5414 | 4-Sep | septin 4 | NM_001198713 | 8.34263 | NaBu/Ct | 4.8155 | 4PBA/Ct | 7.02551 |
| 11720256_x_at | 5414 | 4-Sep | septin 4 | NM_001198713 | 6.38145 | NaBu/Ct | 4.08759 | 4PBA/Ct | 5.64752 |
| 11720319_at | 10615 | SPAG5 | sperm associated antigen 5 | NM_006461 | -6.43652 | NaBu/Ct | -2.68425 | 4PBA/Ct | 4.8619 |
| 11720349_at | 83853 | ROPN1L | ropporin 1-like | NM_031916 | 10.5994 | NaBu/Ct | 1.00325 | 4PBA/Ct | 11.6839 |
| 11720601_x_at | 23208 | SYT11 | synaptotagmin XI | NM_152280 | 7.3635 | NaBu/Ct | 1.21869 | 4PBA/Ct | 7.56103 |
| 11720602_at | 23208 | SYT11 | synaptotagmin XI | NM_152280 | 18.5779 | NaBu/Ct | -1.01519 | 4PBA/Ct | 19.0215 |
| 11720550_a_at | 23310 | NCAPD3 | non-SMC condensin II complex, subunit D3 | NM_015261 | -4.63707 | NaBu/Ct | -2.74953 | 4PBA/Ct | 3.27257 |
| 11720611_a_at | 266812 | NAP1L5 | nucleosome assembly protein 1-like 5 | NM_153757 | 4.83653 | NaBu/Ct | 1.99292 | 4PBA/Ct | 3.27116 |
| 11720612_x_at | 266812 | NAP1L5 | nucleosome assembly protein 1-like 5 | NM_153757 | 5.14874 | NaBu/Ct | 2.56968 | 4PBA/Ct | 3.69349 |
| 11720647_a_at | 701 | BUB1B | budding uninhibited by benzimidazoles 1 homolog beta (yeast) | NM_001211 | -8.87314 | NaBu/Ct | -2.39827 | 4PBA/Ct | 6.38832 |
| 11720835_a_at | 995 | CDC25C | cell division cycle 25 homolog C (S. pombe) | NM_001790 | -5.90378 | NaBu/Ct | -1.71487 | 4PBA/Ct | 5.10638 |
| 11720836_a_at | 54453 | RIN2 | Ras and Rab interactor 2 | NM_018993 | -4.60384 | NaBu/Ct | 1.17855 | 4PBA/Ct | 6.71562 |
| 11720970_at | 7153 | TOP2A | topoisomerase (DNA) II alpha 170kDa | NM_001067 | -3.04148 | NaBu/Ct | -1.28001 | 4PBA/Ct | 2.6769 |
| 11720971_at | 7153 | TOP2A | topoisomerase (DNA) II alpha 170kDa | NM_001067 | -6.25818 | NaBu/Ct | -2.21765 | 4PBA/Ct | 5.58403 |
| 11720972_at | 7153 | TOP2A | topoisomerase (DNA) II alpha 170kDa | NM_001067 | -5.73627 | NaBu/Ct | -2.10763 | 4PBA/Ct | 5.3097 |
| 11721142_a_at | 4288 | MKI67 | antigen identified by monoclonal antibody Ki-67 | NM_001145966 | -5.12733 | NaBu/Ct | -1.86056 | 4PBA/Ct | 4.71419 |
| 11721143_a_at | 4288 | MKI67 | antigen identified by monoclonal antibody Ki-67 | NM_001145966 | -6.67322 | NaBu/Ct | -1.85428 | 4PBA/Ct | 5.0867 |
| 11721144_a_at | 4288 | MKI67 | antigen identified by monoclonal antibody Ki-67 | NM_001145966 | -12.2987 | NaBu/Ct | -3.64565 | 4PBA/Ct | 9.67112 |
| 11721145_s_at | 4288 | MKI67 | antigen identified by monoclonal antibody Ki-67 | NM_001145966 | -15.2624 | NaBu/Ct | -3.55576 | 4PBA/Ct | 10.1663 |
| 11721146_a_at | 4288 | MKI67 | antigen identified by monoclonal antibody Ki-67 | NM_001145966 | -4.7997 | NaBu/Ct | -2.19634 | 4PBA/Ct | 4.59163 |
| 11721539_at | 376267 | RAB15 | RAB15, member RAS onocogene family | NM_198686 | -5.14469 | NaBu/Ct | -1.14988 | 4PBA/Ct | 5.81086 |
| 11721563_a_at | 9833 | MELK | maternal embryonic leucine zipper kinase | NM_014791 | -5.56999 | NaBu/Ct | -2.95031 | 4PBA/Ct | 3.70091 |
| 11721854_a_at | 23397 | NCAPH | non-SMC condensin I complex, subunit H | NM_015341 | -7.47556 | NaBu/Ct | -2.68385 | 4PBA/Ct | 5.19757 |
| 11721923_a_at | 5579 | PRKCB | protein kinase C, beta | NM_002738 | 5.37424 | NaBu/Ct | 3.28659 | 4PBA/Ct | 3.83626 |
| 11721924_s_at | 5579 | PRKCB | protein kinase C, beta | NM_002738 | 8.64315 | NaBu/Ct | 3.9207 | 4PBA/Ct | 6.33971 |
| 11721932_a_at | 9493 | KIF23 | kinesin family member 23 | NM_004856 | -6.18783 | NaBu/Ct | -2.50715 | 4PBA/Ct | 4.32294 |
| 11722069_a_at | 220134 | SKA1 | spindle and kinetochore associated complex subunit 1 | NM_001039535 | -8.33916 | NaBu/Ct | -2.5193 | 4PBA/Ct | 5.41392 |
| 11722072_at | 59274 | MESDC1 | mesoderm development candidate 1 | NM_022566 | -4.40037 | NaBu/Ct | -1.856 | 4PBA/Ct | 3.02375 |
| 11722367_a_at | 9787 | DLGAP5 | discs, large (Drosophila) homolog-associated protein 5 | NM_001146015 | -7.19923 | NaBu/Ct | -3.51673 | 4PBA/Ct | 5.34545 |
| 11722404_s_at | 10588 | MTHFS | 5,10-methenyltetrahydrofolate synthetase (5-formyltetrahydrofolate cyclo-ligase) | NM_006441 | -4.85366 | NaBu/Ct | -1.55388 | 4PBA/Ct | 4.70119 |
| 11722442_a_at | 83540 | NUF2 | NUF2, NDC80 kinetochore complex component, homolog (S. cerevisiae) | NM_031423 | -12.7102 | NaBu/Ct | -3.97369 | 4PBA/Ct | 8.54077 |
| 11722571_at | 890 | CCNA2 | cyclin A2 | NM_001237 | -18.6901 | NaBu/Ct | -3.2868 | 4PBA/Ct | 11.3636 |
| 11722572_at | 890 | CCNA2 | cyclin A2 | NM_001237 | -11.6357 | NaBu/Ct | -4.58173 | 4PBA/Ct | 8.2139 |
| 11722626_at | 23325 | KIAA1033 | KIAA1033 | NM_015275 | 2.50437 | NaBu/Ct | -1.46341 | 4PBA/Ct | 2.45548 |
| 11722627_at | 23325 | KIAA1033 | KIAA1033 | NM_015275 | 3.74502 | NaBu/Ct | 1.07556 | 4PBA/Ct | 2.91666 |
| 11722628_at | 23325 | KIAA1033 | KIAA1033 | NM_015275 | 6.12712 | NaBu/Ct | 2.08731 | 4PBA/Ct | 4.56854 |
| 11722648_a_at | 7137 | TNNI3 | troponin I type 3 (cardiac) | NM_000363 | 7.53797 | NaBu/Ct | 3.60466 | 4PBA/Ct | 5.5741 |
| 11722660_a_at | 9603 | NFE2L3 | nuclear factor (erythroid-derived 2)-like 3 | NM_004289 | 3.92969 | NaBu/Ct | 2.52883 | 4PBA/Ct | 2.63055 |
| 11722661_at | 9603 | NFE2L3 | nuclear factor (erythroid-derived 2)-like 3 | NM_004289 | 4.2719 | NaBu/Ct | 2.09079 | 4PBA/Ct | 2.63506 |
| 11722662_a_at | 10855 | HPSE | heparanase | NM_001098540 | 9.48703 | NaBu/Ct | 2.54473 | 4PBA/Ct | 7.32217 |
| 11722691_at | 57099 | AVEN | apoptosis, caspase activation inhibitor | NM_020371 | -9.07341 | NaBu/Ct | -2.10739 | 4PBA/Ct | 9.20637 |
| 11722825_at | 64151 | NCAPG | non-SMC condensin I complex, subunit G | NM_022346 | -2.26377 | NaBu/Ct | -1.78992 | 4PBA/Ct | 0.923463 |
| 11722826_a_at | 64151 | NCAPG | non-SMC condensin I complex, subunit G | NM_022346 | -8.86856 | NaBu/Ct | -2.86163 | 4PBA/Ct | 6.81705 |
| 11723010_a_at | 10112 | KIF20A | kinesin family member 20A | NM_005733 | -7.84813 | NaBu/Ct | -1.94344 | 4PBA/Ct | 5.61974 |
| 11723030_at | 83716 | CRISPLD2 | cysteine-rich secretory protein LCCL domain containing 2 | NM_031476 | 16.2314 | NaBu/Ct | 2.31506 | 4PBA/Ct | 14.6811 |
| 11723173_at | 54756 | IL17RD | interleukin 17 receptor D | NM_017563 | 4.31606 | NaBu/Ct | 3.43588 | 4PBA/Ct | 3.0211 |
| 11723181_a_at | 5596 | MAPK4 | mitogen-activated protein kinase 4 | NM_002747 | -4.25253 | NaBu/Ct | -1.02342 | 4PBA/Ct | 3.65523 |
| 11723192_a_at | 29128 | UHRF1 | ubiquitin-like with PHD and ring finger domains 1 | NM_001048201 | -5.52863 | NaBu/Ct | -2.53479 | 4PBA/Ct | 4.2678 |
| 11723222_s_at | 9499 | MYOT | myotilin | NM_001135940 | 16.2133 | NaBu/Ct | 1.19705 | 4PBA/Ct | 11.3359 |
| 11723245_a_at | 6422 | SFRP1 | secreted frizzled-related protein 1 | NM_003012 | 5.8624 | NaBu/Ct | 5.72492 | 4PBA/Ct | 4.83979 |
| 11723284_at | 92840 | REEP6 | receptor accessory protein 6 | NM_138393 | 9.41925 | NaBu/Ct | 3.14311 | 4PBA/Ct | 7.64175 |
| 11723435_s_at | 7272 | TTK | TTK protein kinase | NM_001166691 | -9.80718 | NaBu/Ct | -2.53868 | 4PBA/Ct | 7.94666 |
| 11723447_at | 7851 | MALL | mal, T-cell differentiation protein-like | NM_005434 | -6.75068 | NaBu/Ct | 1.01237 | 4PBA/Ct | 8.33693 |
| 11723448_x_at | 7851 | MALL | mal, T-cell differentiation protein-like | NM_005434 | -7.96115 | NaBu/Ct | -1.00467 | 4PBA/Ct | 9.07739 |
| 11723524_at | 155465 | AGR3 | anterior gradient homolog 3 (Xenopus laevis) | NM_176813 | -8.06469 | NaBu/Ct | -1.10339 | 4PBA/Ct | 10.7687 |
| 11723565_a_at | 1058 | CENPA | centromere protein A | NM_001042426 | -13.4798 | NaBu/Ct | -2.28398 | 4PBA/Ct | 8.96861 |
| 11723691_at | 9936 | CD302 | CD302 molecule | NM_001198763 | 6.28793 | NaBu/Ct | 4.21227 | 4PBA/Ct | 4.61202 |
| 11723692_a_at | 9936 | CD302 | CD302 molecule | NM_001198763 | 5.52668 | NaBu/Ct | 2.62797 | 4PBA/Ct | 3.83014 |
| 11723885_at | 256302 | C17orf103 | chromosome 17 open reading frame 103 | NM_152914 | 15.7631 | NaBu/Ct | 2.71926 | 4PBA/Ct | 12.408 |
| 11723939_a_at | 891 | CCNB1 | cyclin B1 | NM_031966 | -6.59421 | NaBu/Ct | -2.18287 | 4PBA/Ct | 4.64051 |
| 11723950_a_at | 55872 | PBK | PDZ binding kinase | NM_018492 | -11.5269 | NaBu/Ct | -2.46477 | 4PBA/Ct | 7.89949 |
| 11723998_at | 57405 | SPC25 | SPC25, NDC80 kinetochore complex component, homolog (S. cerevisiae) | NM_020675 | -27.9581 | NaBu/Ct | -3.16386 | 4PBA/Ct | 16.477 |
| 11724021_at | 3017 | HIST1H2BD | histone cluster 1, H2bd | NM_021063 | 7.16943 | NaBu/Ct | 2.58757 | 4PBA/Ct | 5.98855 |
| 11724328_a_at | 11065 | UBE2C | ubiquitin-conjugating enzyme E2C | NM_007019 | -11.5836 | NaBu/Ct | -2.84246 | 4PBA/Ct | 8.96937 |
| 11724488_at | 57016 | AKR1B10 | aldo-keto reductase family 1, member B10 (aldose reductase) | NM_020299 | -6.86756 | NaBu/Ct | -1.20012 | 4PBA/Ct | 8.01519 |
| 11724489_s_at | 441282 | AKR1B10 | aldo-keto reductase family 1, member B10 (aldose reductase) | NM_001080538 | -12.8934 | NaBu/Ct | -1.72046 | 4PBA/Ct | 12.3449 |
| 11724490_x_at | 57016 | AKR1B10 | aldo-keto reductase family 1, member B10 (aldose reductase) | NM_020299 | -7.52469 | NaBu/Ct | -1.17957 | 4PBA/Ct | 8.75355 |
| 11724585_a_at | 6857 | SYT1 | synaptotagmin I | NM_001135805 | 8.78663 | NaBu/Ct | 4.43797 | 4PBA/Ct | 6.46019 |
| 11724586_a_at | 6857 | SYT1 | synaptotagmin I | NM_001135805 | 5.68106 | NaBu/Ct | 2.60214 | 4PBA/Ct | 3.69464 |
| 11724587_a_at | 6857 | SYT1 | synaptotagmin I | NM_001135805 | 6.11608 | NaBu/Ct | 3.19986 | 4PBA/Ct | 4.27568 |
| 11724828_at | 6364 | CCL20 | chemokine (C-C motif) ligand 20 | NM_001130046 | 14.0494 | NaBu/Ct | 2.72414 | 4PBA/Ct | 13.2792 |
| 11724963_s_at | 26271 | FBXO5 | F-box protein 5 | NM_001142522 | -9.93826 | NaBu/Ct | -3.05188 | 4PBA/Ct | 8.23455 |
| 11725198_at | 3552 | IL1A | interleukin 1, alpha | NM_000575 | 4.65444 | NaBu/Ct | 3.83786 | 4PBA/Ct | 3.90992 |
| 11725429_at | 27074 | LAMP3 | lysosomal-associated membrane protein 3 | NM_014398 | 12.4393 | NaBu/Ct | 2.60491 | 4PBA/Ct | 8.46522 |
| 11725441_a_at | 2305 | FOXM1 | forkhead box M1 | NM_021953 | -10.6402 | NaBu/Ct | -2.98716 | 4PBA/Ct | 8.00118 |
| 11725504_a_at | 10220 | GDF11 | growth differentiation factor 11 | NM_005811 | 3.89964 | NaBu/Ct | 2.1364 | 4PBA/Ct | 3.72523 |
| 11725506_s_at | 10220 | GDF11 | growth differentiation factor 11 | NM_005811 | 3.28584 | NaBu/Ct | 1.688 | 4PBA/Ct | 3.02958 |
| 11725533_at | 114991 | ZNF618 | zinc finger protein 618 | NM_133374 | -4.19839 | NaBu/Ct | -1.33338 | 4PBA/Ct | 3.25993 |
| 11725722_at | 29028 | ATAD2 | ATPase family, AAA domain containing 2 | NM_014109 | -6.23346 | NaBu/Ct | -3.94124 | 4PBA/Ct | 5.62786 |
| 11725742_a_at | 10580 | SORBS1 | sorbin and SH3 domain containing 1 | NM_001034954 | 2.31772 | NaBu/Ct | 1.35632 | 4PBA/Ct | 0.949043 |
| 11725743_s_at | 10580 | SORBS1 | sorbin and SH3 domain containing 1 | NM_001034954 | 7.63219 | NaBu/Ct | 1.68574 | 4PBA/Ct | 5.74563 |
| 11725744_x_at | 10580 | SORBS1 | sorbin and SH3 domain containing 1 | NM_001034954 | 4.69603 | NaBu/Ct | 1.53931 | 4PBA/Ct | 4.73686 |
| 11725842_a_at | 80119 | PIF1 | PIF1 5'-to-3' DNA helicase homolog (S. cerevisiae) | NM_025049 | -14.3119 | NaBu/Ct | -3.17513 | 4PBA/Ct | 9.51932 |
| 11725979_a_at | 793 | CALB1 | calbindin 1, 28kDa | NM_004929 | 14.9013 | NaBu/Ct | -1.01693 | 4PBA/Ct | 14.4408 |
| 11725982_at | 170261 | ZCCHC12 | zinc finger, CCHC domain containing 12 | NM_173798 | 5.07442 | NaBu/Ct | 1.41241 | 4PBA/Ct | 4.79195 |
| 11725983_at | 8553 | BHLHE40 | basic helix-loop-helix family, member e40 | NM_003670 | -3.96641 | NaBu/Ct | 1.4633 | 4PBA/Ct | 4.99746 |
| 11726302_a_at | 51514 | DTL | denticleless homolog (Drosophila) | NM_016448 | -12.237 | NaBu/Ct | -5.22413 | 4PBA/Ct | 9.0241 |
| 11726330_a_at | 9963 | SLC23A1 | solute carrier family 23 (nucleobase transporters), member 1 | NM_005847 | -10.0532 | NaBu/Ct | -1.47381 | 4PBA/Ct | 8.79418 |
| 11726360_a_at | 218 | ALDH3A1 | aldehyde dehydrogenase 3 family, member A1 | NM_000691 | -4.31929 | NaBu/Ct | 1.28662 | 4PBA/Ct | 4.82881 |
| 11726378_a_at | 7468 | WHSC1 | Wolf-Hirschhorn syndrome candidate 1 | NM_001042424 | -3.4798 | NaBu/Ct | -1.81718 | 4PBA/Ct | 2.77608 |
| 11726441_a_at | 54821 | ERCC6L | excision repair cross-complementing rodent repair deficiency, complementation group 6-l | NM_017669 | -5.71693 | NaBu/Ct | -2.70102 | 4PBA/Ct | 4.28383 |
| 11726551_s_at | 154064 | RAET1L | retinoic acid early transcript 1L | NM_025217 | 22.0519 | NaBu/Ct | 6.89279 | 4PBA/Ct | 14.8644 |
| 11726552_x_at | 80328 | ULBP2 | UL16 binding protein 2 | NM_025217 | 19.3083 | NaBu/Ct | 5.49848 | 4PBA/Ct | 13.8363 |
| 11726617_s_at | 983 | CDK1 | cyclin-dependent kinase 1 | NM_001170406 | -10.5921 | NaBu/Ct | -2.69514 | 4PBA/Ct | 8.75393 |
| 11726756_a_at | 993 | CDC25A | cell division cycle 25 homolog A (S. pombe) | NM_001789 | -10.5035 | NaBu/Ct | -7.84245 | 4PBA/Ct | 7.93976 |
| 11726757_s_at | 993 | CDC25A | cell division cycle 25 homolog A (S. pombe) | NM_001789 | -7.47047 | NaBu/Ct | -4.54556 | 4PBA/Ct | 5.49359 |
| 11727014_at | 5069 | PAPPA | pregnancy-associated plasma protein A, pappalysin 1 | NM_002581 | -6.23116 | NaBu/Ct | 1.01126 | 4PBA/Ct | 7.44859 |
| 11727015_s_at | 5069 | PAPPA | pregnancy-associated plasma protein A, pappalysin 1 | NM_002581 | -6.88936 | NaBu/Ct | -1.22886 | 4PBA/Ct | 9.09806 |
| 11727016_s_at | 5069 | PAPPA | pregnancy-associated plasma protein A, pappalysin 1 | NM_002581 | -11.3524 | NaBu/Ct | 1.12578 | 4PBA/Ct | 13.5007 |
| 11727017_s_at | 5069 | PAPPA | pregnancy-associated plasma protein A, pappalysin 1 | NM_002581 | -2.40884 | NaBu/Ct | -1.36815 | 4PBA/Ct | 1.0261 |
| 11727018_s_at | 5069 | PAPPA | pregnancy-associated plasma protein A, pappalysin 1 | NM_002581 | -4.71998 | NaBu/Ct | 1.09725 | 4PBA/Ct | 7.69933 |
| 11727102_a_at | 10642 | IGF2BP1 | insulin-like growth factor 2 mRNA binding protein 1 | NM_001160423 | -6.04395 | NaBu/Ct | -2.23558 | 4PBA/Ct | 3.94207 |
| 11727103_at | 10642 | IGF2BP1 | insulin-like growth factor 2 mRNA binding protein 1 | NM_001160423 | -5.23653 | NaBu/Ct | -1.16313 | 4PBA/Ct | 6.97095 |
| 11727489_a_at | 3832 | KIF11 | kinesin family member 11 | NM_004523 | -6.08494 | NaBu/Ct | -2.86486 | 4PBA/Ct | 4.396 |
| 11727490_x_at | 3832 | KIF11 | kinesin family member 11 | NM_004523 | -4.33396 | NaBu/Ct | -2.64306 | 4PBA/Ct | 2.7257 |
| 11727535_a_at | 899 | CCNF | cyclin F | NM_001761 | -2.57608 | NaBu/Ct | -2.1278 | 4PBA/Ct | 1.08982 |
| 11727536_x_at | 899 | CCNF | cyclin F | NM_001761 | -4.10589 | NaBu/Ct | -2.9738 | 4PBA/Ct | 2.47123 |
| 11727556_a_at | 29841 | GRHL1 | grainyhead-like 1 (Drosophila) | NM_198182 | 4.39695 | NaBu/Ct | 3.54094 | 4PBA/Ct | 3.51619 |
| 11727623_at | 493861 | EID3 | EP300 interacting inhibitor of differentiation 3 | NM_001008394 | 12.6053 | NaBu/Ct | -2.85008 | 4PBA/Ct | 19.3123 |
| 11727679_at | 7536 | SF1 | splicing factor 1 | NM_001178030 | 2.03143 | NaBu/Ct | 1.64388 | 4PBA/Ct | 0.551549 |
| 11727684_at | 4916 | NTRK3 | neurotrophic tyrosine kinase, receptor, type 3 | NM_001007156 | -4.62751 | NaBu/Ct | -1.35467 | 4PBA/Ct | 5.07194 |
| 11727685_at | 4916 | NTRK3 | neurotrophic tyrosine kinase, receptor, type 3 | NM_001007156 | -4.81196 | NaBu/Ct | -1.3416 | 4PBA/Ct | 4.20702 |
| 11727837_at | 23594 | ORC6 | origin recognition complex, subunit 6 | NM_014321 | -10.3838 | NaBu/Ct | -3.84402 | 4PBA/Ct | 6.93408 |
| 11727916_a_at | 1058 | CENPA | centromere protein A | NM_001042426 | -12.5975 | NaBu/Ct | -2.22559 | 4PBA/Ct | 8.23995 |
| 11727968_at | 157570 | ESCO2 | establishment of cohesion 1 homolog 2 (S. cerevisiae) | NM_001017420 | -10.7714 | NaBu/Ct | -3.38884 | 4PBA/Ct | 6.49833 |
| 11727972_a_at | 55839 | CENPN | centromere protein N | NM_001100624 | -7.18708 | NaBu/Ct | -2.63869 | 4PBA/Ct | 5.1214 |
| 11727973_a_at | 55839 | CENPN | centromere protein N | NM_001100624 | -5.16565 | NaBu/Ct | -2.42289 | 4PBA/Ct | 3.19646 |
| 11728054_a_at | 81624 | DIAPH3 | diaphanous homolog 3 (Drosophila) | NM_001042517 | -5.69107 | NaBu/Ct | -2.17266 | 4PBA/Ct | 4.94756 |
| 11728101_at | 195828 | ZNF367 | zinc finger protein 367 | NM_153695 | -4.50553 | NaBu/Ct | -6.34146 | 4PBA/Ct | 4.5803 |
| 11728152_a_at | 3017 | HIST1H2BD | histone cluster 1, H2bd | NM_021063 | 4.99664 | NaBu/Ct | 2.01285 | 4PBA/Ct | 4.68897 |
| 11728153_x_at | 3017 | HIST1H2BD | histone cluster 1, H2bd | NM_021063 | 5.98679 | NaBu/Ct | 1.76773 | 4PBA/Ct | 5.60752 |
| 11728176_a_at | 353500 | BMP8A | bone morphogenetic protein 8a | NM_181809 | 10.1812 | NaBu/Ct | 1.94709 | 4PBA/Ct | 10.1786 |
| 11728178_at | 23409 | SIRT4 | sirtuin 4 | NM_012240 | 5.46412 | NaBu/Ct | 2.22751 | 4PBA/Ct | 4.79279 |
| 11728220_at | 56675 | NRIP3 | nuclear receptor interacting protein 3 | NM_020645 | 6.41529 | NaBu/Ct | 3.58483 | 4PBA/Ct | 5.45753 |
| 11728221_at | 56675 | NRIP3 | nuclear receptor interacting protein 3 | NM_020645 | 3.11648 | NaBu/Ct | 2.10703 | 4PBA/Ct | 2.27074 |
| 11728379_at | 84288 | EFCAB2 | EF-hand calcium binding domain 2 | NM_001143943 | -5.58586 | NaBu/Ct | -1.97517 | 4PBA/Ct | 4.79643 |
| 11728403_at | 79801 | SHCBP1 | SHC SH2-domain binding protein 1 | NM_024745 | -3.47471 | NaBu/Ct | -1.4938 | 4PBA/Ct | 2.13125 |
| 11728404_at | 79801 | SHCBP1 | SHC SH2-domain binding protein 1 | NM_024745 | -7.91194 | NaBu/Ct | -2.34386 | 4PBA/Ct | 5.74699 |
| 11728669_a_at | 7328 | UBE2H | ubiquitin-conjugating enzyme E2H (UBC8 homolog, yeast) | NM_003344 | 3.76672 | NaBu/Ct | 2.37146 | 4PBA/Ct | 2.19885 |
| 11728670_a_at | 7328 | UBE2H | ubiquitin-conjugating enzyme E2H (UBC8 homolog, yeast) | NM_003344 | 4.53541 | NaBu/Ct | 1.90012 | 4PBA/Ct | 3.00321 |
| 11728671_s_at | 7328 | UBE2H | ubiquitin-conjugating enzyme E2H (UBC8 homolog, yeast) | NM_003344 | 2.91782 | NaBu/Ct | 1.62538 | 4PBA/Ct | 1.35242 |
| 11728672_x_at | 7328 | UBE2H | ubiquitin-conjugating enzyme E2H (UBC8 homolog, yeast) | NM_003344 | 3.00549 | NaBu/Ct | 1.5352 | 4PBA/Ct | 1.54854 |
| 11728673_s_at | 3835 | KIF22 | kinesin family member 22 | NM_007317 | -6.9505 | NaBu/Ct | -2.73128 | 4PBA/Ct | 4.83789 |
| 11728715_at | 6374 | CXCL5 | chemokine (C-X-C motif) ligand 5 | NM_002994 | -11.7192 | NaBu/Ct | 1.27079 | 4PBA/Ct | 21.1513 |
| 11728716_x_at | 6374 | CXCL5 | chemokine (C-X-C motif) ligand 5 | NM_002994 | -11.7696 | NaBu/Ct | 1.24798 | 4PBA/Ct | 21.3938 |
| 11728717_at | 6374 | CXCL5 | chemokine (C-X-C motif) ligand 5 | NM_002994 | -8.90729 | NaBu/Ct | 1.54771 | 4PBA/Ct | 20.9568 |
| 11728729_at | 79075 | DSCC1 | defective in sister chromatid cohesion 1 homolog (S. cerevisiae) | NM_024094 | -10.7506 | NaBu/Ct | -3.75315 | 4PBA/Ct | 7.24513 |
| 11728731_s_at | 79075 | DSCC1 | defective in sister chromatid cohesion 1 homolog (S. cerevisiae) | NM_024094 | -8.63745 | NaBu/Ct | -2.72412 | 4PBA/Ct | 7.31058 |
| 11728754_at | 400629 | TEX19 | testis expressed 19 | NM_207459 | 30.1295 | NaBu/Ct | 1.91255 | 4PBA/Ct | 19.3895 |
| 11728848_at | 116442 | RAB39B | RAB39B, member RAS oncogene family | NM_171998 | 3.18359 | NaBu/Ct | 1.58191 | 4PBA/Ct | 2.77443 |
| 11728849_at | 116442 | RAB39B | RAB39B, member RAS oncogene family | NM_171998 | 2.97204 | NaBu/Ct | 1.21898 | 4PBA/Ct | 1.87859 |
| 11728850_at | 116442 | RAB39B | RAB39B, member RAS oncogene family | NM_171998 | 3.09178 | NaBu/Ct | 1.57702 | 4PBA/Ct | 3.60775 |
| 11728851_s_at | 116442 | RAB39B | RAB39B, member RAS oncogene family | NM_171998 | 16.4698 | NaBu/Ct | 4.04589 | 4PBA/Ct | 12.4619 |
| 11729575_a_at | 7494 | XBP1 | X-box binding protein 1 | NM_001079539 | -5.68621 | NaBu/Ct | -1.38175 | 4PBA/Ct | 5.14666 |
| 11729646_at | 7104 | TM4SF4 | transmembrane 4 L six family member 4 | NM_004617 | -8.27966 | NaBu/Ct | -1.46691 | 4PBA/Ct | 7.18337 |
| 11729663_a_at | 1111 | CHEK1 | CHK1 checkpoint homolog (S. pombe) | NM_001114121 | -7.27049 | NaBu/Ct | -3.52649 | 4PBA/Ct | 6.24441 |
| 11730029_a_at | 144455 | E2F7 | E2F transcription factor 7 | NM_203394 | -13.7327 | NaBu/Ct | -3.09469 | 4PBA/Ct | 9.5509 |
| 11728957_a_at | 79733 | E2F8 | E2F transcription factor 8 | NM_024680 | -4.78155 | NaBu/Ct | -2.91947 | 4PBA/Ct | 2.85429 |
| 11728958_x_at | 79733 | E2F8 | E2F transcription factor 8 | NM_024680 | -7.68098 | NaBu/Ct | -3.58986 | 4PBA/Ct | 6.06476 |
| 11730111_a_at | 55635 | DEPDC1 | DEP domain containing 1 | NM_001114120 | -12.9542 | NaBu/Ct | -2.58088 | 4PBA/Ct | 9.27494 |
| 11730112_a_at | 55635 | DEPDC1 | DEP domain containing 1 | NM_001114120 | -12.431 | NaBu/Ct | -1.94544 | 4PBA/Ct | 10.3337 |
| 11730757_a_at | 3572 | IL6ST | interleukin 6 signal transducer (gp130, oncostatin M receptor) | NM_001190981 | 4.93052 | NaBu/Ct | 1.89926 | 4PBA/Ct | 2.7969 |
| 11730820_at | 56126 | PCDHB10 | protocadherin beta 10 | NM_018930 | 4.7344 | NaBu/Ct | 1.20826 | 4PBA/Ct | 3.01802 |
| 11731460_at | 1368 | CPM | carboxypeptidase M | NM_001005502 | 6.85141 | NaBu/Ct | 4.2989 | 4PBA/Ct | 5.55399 |
| 11731461_x_at | 1368 | CPM | carboxypeptidase M | NM_001005502 | 11.7415 | NaBu/Ct | 5.85564 | 4PBA/Ct | 9.47681 |
| 11731463_x_at | 1368 | CPM | carboxypeptidase M | NM_001005502 | 2.24324 | NaBu/Ct | 2.17108 | 4PBA/Ct | 1.11486 |
| 11731464_at | 1368 | CPM | carboxypeptidase M | NM_001005502 | 6.04869 | NaBu/Ct | 3.67508 | 4PBA/Ct | 5.14262 |
| 11731525_at | 9319 | TRIP13 | thyroid hormone receptor interactor 13 | NM_001166260 | -5.20472 | NaBu/Ct | -3.44264 | 4PBA/Ct | 3.48489 |
| 11731526_s_at | 9319 | TRIP13 | thyroid hormone receptor interactor 13 | NM_001166260 | -4.3533 | NaBu/Ct | -2.8472 | 4PBA/Ct | 2.78704 |
| 11731782_s_at | 441519 /// 441520 /// 441521 /// 541465 /// 541466 /// 728911 | CT45A1 /// CT45A2 /// CT45A3 /// CT45A4 /// CT45A5 /// CT45A6 | cancer/testis antigen family 45, member A1 /// cancer/testis antigen family 45, member | NM_001007551 | 14.9207 | NaBu/Ct | 6.51109 | 4PBA/Ct | 9.6472 |
| 11731848_s_at | 3821 /// 3822 | KLRC1 /// KLRC2 | killer cell lectin-like receptor subfamily C, member 1 /// killer cell lectin-like rece | NM_002259 | 14.9748 | NaBu/Ct | 1.8119 | 4PBA/Ct | 12.7244 |
| 11731887_at | 9928 | KIF14 | kinesin family member 14 | NM_014875 | -8.54342 | NaBu/Ct | -2.29176 | 4PBA/Ct | 7.16756 |
| 11732191_s_at | 10635 | RAD51AP1 | RAD51 associated protein 1 | NM_001130862 | -14.1588 | NaBu/Ct | -4.13816 | 4PBA/Ct | 9.5085 |
| 11732192_a_at | 10635 | RAD51AP1 | RAD51 associated protein 1 | NM_001130862 | -5.47507 | NaBu/Ct | -2.65979 | 4PBA/Ct | 5.3852 |
| 11732193_a_at | 4291 | MLF1 | myeloid leukemia factor 1 | NM_001130156 | 12.6522 | NaBu/Ct | 4.72557 | 4PBA/Ct | 9.92438 |
| 11732321_a_at | 5327 | PLAT | plasminogen activator, tissue | NM_000930 | 13.1082 | NaBu/Ct | 4.57114 | 4PBA/Ct | 9.01504 |
| 11732339_at | 53335 | BCL11A | B-cell CLL/lymphoma 11A (zinc finger protein) | NM_018014 | 4.86711 | NaBu/Ct | 5.72726 | 4PBA/Ct | 3.86904 |
| 11732341_at | 53335 | BCL11A | B-cell CLL/lymphoma 11A (zinc finger protein) | NM_018014 | 3.47488 | NaBu/Ct | 4.10049 | 4PBA/Ct | 2.49202 |
| 11732415_s_at | 7074 | TIAM1 | T-cell lymphoma invasion and metastasis 1 | NM_003253 | 5.22108 | NaBu/Ct | 4.33038 | 4PBA/Ct | 4.2636 |
| 11732453_s_at | 3081 | HGD | homogentisate 1,2-dioxygenase | NM_000187 | -31.571 | NaBu/Ct | 1.32248 | 4PBA/Ct | 25.7152 |
| 11732465_a_at | 84101 | USP44 | ubiquitin specific peptidase 44 | NM_001042403 | 5.96059 | NaBu/Ct | 3.38853 | 4PBA/Ct | 4.45981 |
| 11732637_a_at | 312 | ANXA13 | annexin A13 | NM_001003954 | -18.2684 | NaBu/Ct | 1.27408 | 4PBA/Ct | 19.5568 |
| 11732858_a_at | 25960 | GPR124 | G protein-coupled receptor 124 | NM_032777 | 5.99474 | NaBu/Ct | 3.49127 | 4PBA/Ct | 3.9771 |
| 11732922_at | 2700 | GJA3 | gap junction protein, alpha 3, 46kDa | NM_021954 | 7.14669 | NaBu/Ct | 2.47894 | 4PBA/Ct | 5.90431 |
| 11732927_x_at | 3821 | KLRC1 | killer cell lectin-like receptor subfamily C, member 1 | NM_002259 | 7.27738 | NaBu/Ct | 1.16355 | 4PBA/Ct | 7.3675 |
| 11732997_a_at | 4175 | MCM6 | minichromosome maintenance complex component 6 | NM_005915 | -4.88876 | NaBu/Ct | -2.56339 | 4PBA/Ct | 3.4537 |
| 11732998_at | 4175 | MCM6 | minichromosome maintenance complex component 6 | NM_005915 | -4.22985 | NaBu/Ct | -2.07753 | 4PBA/Ct | 2.9943 |
| 11733091_a_at | 7103 | TSPAN8 | tetraspanin 8 | NM_004616 | -10.0416 | NaBu/Ct | 1.25334 | 4PBA/Ct | 11.8004 |
| 11733522_a_at | 154661 | RUNDC3B | RUN domain containing 3B | NM_001134405 | 10.463 | NaBu/Ct | 2.25886 | 4PBA/Ct | 7.65661 |
| 11733695_a_at | 11065 | UBE2C | ubiquitin-conjugating enzyme E2C | NM_007019 | -19.0077 | NaBu/Ct | -2.83599 | 4PBA/Ct | 14.058 |
| 11733696_x_at | 11065 | UBE2C | ubiquitin-conjugating enzyme E2C | NM_007019 | -14.1663 | NaBu/Ct | -2.98532 | 4PBA/Ct | 10.3074 |
| 11733702_x_at | 11065 | UBE2C | ubiquitin-conjugating enzyme E2C | NM_007019 | -14.0738 | NaBu/Ct | -2.46074 | 4PBA/Ct | 12.797 |
| 11733864_a_at | 332 | BIRC5 | baculoviral IAP repeat-containing 5 | NM_001012270 | -6.13574 | NaBu/Ct | -2.11895 | 4PBA/Ct | 4.95066 |
| 11734006_a_at | 3654 | IRAK1 | interleukin-1 receptor-associated kinase 1 | NM_001025242 | -5.57242 | NaBu/Ct | -1.47751 | 4PBA/Ct | 3.86416 |
| 11735139_at | 8744 | TNFSF9 | tumor necrosis factor (ligand) superfamily, member 9 | NM_003811 | 5.93311 | NaBu/Ct | 1.46212 | 4PBA/Ct | 4.84236 |
| 11735140_at | 8744 | TNFSF9 | tumor necrosis factor (ligand) superfamily, member 9 | NM_003811 | 9.27674 | NaBu/Ct | 1.40429 | 4PBA/Ct | 8.77382 |
| 11735171_at | 79853 | TM4SF20 | transmembrane 4 L six family member 20 | NM_024795 | -15.8777 | NaBu/Ct | 1.04843 | 4PBA/Ct | 14.5553 |
| 11735172_at | 79853 | TM4SF20 | transmembrane 4 L six family member 20 | NM_024795 | -21.3029 | NaBu/Ct | 1.26647 | 4PBA/Ct | 20.9062 |
| 11735331_a_at | 154661 | RUNDC3B | RUN domain containing 3B | NM_001134405 | 13.3831 | NaBu/Ct | 2.15149 | 4PBA/Ct | 10.4278 |
| 11735584_x_at | 672 | BRCA1 | breast cancer 1, early onset | NM_007294 | -6.87484 | NaBu/Ct | -4.1077 | 4PBA/Ct | 5.42235 |
| 11736068_x_at | 65009 | NDRG4 | NDRG family member 4 | NM_001130487 | 5.49185 | NaBu/Ct | 3.10421 | 4PBA/Ct | 4.80996 |
| 11736082_a_at | 51512 | GTSE1 | G-2 and S-phase expressed 1 | NM_016426 | -2.22122 | NaBu/Ct | -1.43182 | 4PBA/Ct | 0.811318 |
| 11736083_x_at | 51512 | GTSE1 | G-2 and S-phase expressed 1 | NM_016426 | -8.99893 | NaBu/Ct | -3.59884 | 4PBA/Ct | 6.13614 |
| 11736101_at | 4121 | MAN1A1 | mannosidase, alpha, class 1A, member 1 | NM_005907 | 5.39804 | NaBu/Ct | 1.46872 | 4PBA/Ct | 3.96748 |
| 11736124_a_at | 10814 | CPLX2 | complexin 2 | NM_001008220 | -13.2483 | NaBu/Ct | -1.78616 | 4PBA/Ct | 10.2007 |
| 11736125_a_at | 10814 | CPLX2 | complexin 2 | NM_001008220 | -4.87573 | NaBu/Ct | -1.52978 | 4PBA/Ct | 3.67319 |
| 11736126_x_at | 10814 | CPLX2 | complexin 2 | NM_001008220 | -2.84909 | NaBu/Ct | -1.48472 | 4PBA/Ct | 2.01155 |
| 11736280_at | 6581 | SLC22A3 | solute carrier family 22 (extraneuronal monoamine transporter), member 3 | NM_021977 | -11.6164 | NaBu/Ct | -1.39037 | 4PBA/Ct | 10.5441 |
| 11736281_a_at | 6581 | SLC22A3 | solute carrier family 22 (extraneuronal monoamine transporter), member 3 | NM_021977 | -13.8503 | NaBu/Ct | -1.42135 | 4PBA/Ct | 16.0229 |
| 11736348_a_at | 3077 | HFE | hemochromatosis | NM_000410 | -5.31832 | NaBu/Ct | -1.62045 | 4PBA/Ct | 4.30618 |
| 11736366_x_at | 55388 | MCM10 | minichromosome maintenance complex component 10 | NM_018518 | -10.4498 | NaBu/Ct | -5.07912 | 4PBA/Ct | 7.56431 |
| 11736367_a_at | 55388 | MCM10 | minichromosome maintenance complex component 10 | NM_018518 | -9.86389 | NaBu/Ct | -3.83421 | 4PBA/Ct | 6.54351 |
| 11736368_a_at | 55388 | MCM10 | minichromosome maintenance complex component 10 | NM_018518 | -6.89539 | NaBu/Ct | -4.67396 | 4PBA/Ct | 5.3102 |
| 11736670_a_at | 4173 | MCM4 | minichromosome maintenance complex component 4 | NM_005914 | -6.18467 | NaBu/Ct | -3.28102 | 4PBA/Ct | 4.80208 |
| 11736800_a_at | 10846 | PDE10A | phosphodiesterase 10A | NM_001130690 | -7.16851 | NaBu/Ct | -1.24026 | 4PBA/Ct | 7.4546 |
| 11737210_a_at | 3068 | HDGF | hepatoma-derived growth factor | NM_001126050 | -6.24743 | NaBu/Ct | -2.34444 | 4PBA/Ct | 5.26712 |
| 11737211_x_at | 3068 | HDGF | hepatoma-derived growth factor | NM_001126050 | -5.31103 | NaBu/Ct | -2.14109 | 4PBA/Ct | 4.69944 |
| 11737257_at | 257629 | ANKS4B | ankyrin repeat and sterile alpha motif domain containing 4B | NM_145865 | -4.94449 | NaBu/Ct | -1.63434 | 4PBA/Ct | 3.61069 |
| 11737258_x_at | 257629 | ANKS4B | ankyrin repeat and sterile alpha motif domain containing 4B | NM_145865 | -4.59899 | NaBu/Ct | -1.41521 | 4PBA/Ct | 3.56667 |
| 11737593_at | 1E+08 | KILLIN | killin protein | NM_001126049 | 29.0985 | NaBu/Ct | 1.73631 | 4PBA/Ct | 17.8963 |
| 11737795_a_at | 57650 | KIAA1524 | KIAA1524 | NM_020890 | -5.07963 | NaBu/Ct | -1.82616 | 4PBA/Ct | 3.97285 |
| 11737796_a_at | 57650 | KIAA1524 | KIAA1524 | NM_020890 | -9.18045 | NaBu/Ct | -4.19134 | 4PBA/Ct | 6.1393 |
| 11738250_at | 1111 | CHEK1 | CHK1 checkpoint homolog (S. pombe) | NM_001114121 | -5.74579 | NaBu/Ct | -1.90093 | 4PBA/Ct | 4.00161 |
| 11738251_x_at | 1111 | CHEK1 | CHK1 checkpoint homolog (S. pombe) | NM_001114121 | -5.18034 | NaBu/Ct | -1.90635 | 4PBA/Ct | 3.65532 |
| 11738935_a_at | 3823 | KLRC3 | killer cell lectin-like receptor subfamily C, member 3 | NM_002261 | 13.3802 | NaBu/Ct | 1.56677 | 4PBA/Ct | 9.58308 |
| 11738936_x_at | 3823 | KLRC3 | killer cell lectin-like receptor subfamily C, member 3 | NM_002261 | 5.76084 | NaBu/Ct | 1.2207 | 4PBA/Ct | 4.37376 |
| 11739002_a_at | 3068 | HDGF | hepatoma-derived growth factor | NM_001126050 | -5.64037 | NaBu/Ct | -2.20768 | 4PBA/Ct | 4.84742 |
| 11739004_x_at | 3068 | HDGF | hepatoma-derived growth factor | NM_001126050 | -4.05323 | NaBu/Ct | -1.97349 | 4PBA/Ct | 2.64319 |
| 11739144_a_at | 6502 | SKP2 | S-phase kinase-associated protein 2 (p45) | NM_005983 | -5.21957 | NaBu/Ct | -4.41954 | 4PBA/Ct | 4.10159 |
| 11739653_a_at | 332 | BIRC5 | baculoviral IAP repeat-containing 5 | NM_001012270 | -6.8695 | NaBu/Ct | -2.44869 | 4PBA/Ct | 4.66843 |
| 11739787_a_at | 3481 | IGF2 | insulin-like growth factor 2 (somatomedin A) | NM_000612 | 31.0013 | NaBu/Ct | 48.8447 | 4PBA/Ct | 20.2622 |
| 11739916_a_at | 89778 | SERPINB11 | serpin peptidase inhibitor, clade B (ovalbumin), member 11 (gene/pseudogene) | NM_080475 | -24.2056 | NaBu/Ct | -1.05894 | 4PBA/Ct | 18.7035 |
| 11739932_a_at | 374393 | FAM111B | family with sequence similarity 111, member B | NM_001142703 | -9.61145 | NaBu/Ct | -4.88005 | 4PBA/Ct | 6.16002 |
| 11740253_a_at | 11130 | ZWINT | ZW10 interactor | NM_001005413 | -9.29116 | NaBu/Ct | -3.00232 | 4PBA/Ct | 6.45897 |
| 11740393_at | 3604 | TNFRSF9 | tumor necrosis factor receptor superfamily, member 9 | NM_001561 | 5.37266 | NaBu/Ct | 1.1615 | 4PBA/Ct | 6.81409 |
| 11740656_a_at | 8091 | HMGA2 | high mobility group AT-hook 2 | NM_003483 | -4.86157 | NaBu/Ct | 1.07777 | 4PBA/Ct | 7.8885 |
| 11741015_a_at | 55143 | CDCA8 | cell division cycle associated 8 | NM_018101 | -11.0486 | NaBu/Ct | -2.94952 | 4PBA/Ct | 7.23274 |
| 11741032_a_at | 3161 | HMMR | hyaluronan-mediated motility receptor (RHAMM) | NM_001142556 | -7.18208 | NaBu/Ct | -2.80746 | 4PBA/Ct | 5.89729 |
| 11741559_a_at | 312 | ANXA13 | annexin A13 | NM_001003954 | -16.4516 | NaBu/Ct | 1.32444 | 4PBA/Ct | 19.2303 |
| 11742378_a_at | 441282 /57016 | AKR1B10 /AKR1B15 | aldo-keto reductase family 1, member B10 (aldose reductase) /// aldo-keto reductase fam | NM_001080538 | -12.3996 | NaBu/Ct | -1.41959 | 4PBA/Ct | 12.8679 |
| 11742379_x_at | 441282 | AKR1B15 | aldo-keto reductase family 1, member B15 | NM_001080538 | -10.708 | NaBu/Ct | -1.35445 | 4PBA/Ct | 10.7255 |
| 11742657_x_at | 723790 /// 8337 | HIST2H2AA3 /// HIST2H2AA4 | histone cluster 2, H2aa3 / histone cluster 2, H2aa4 | NM_001040874 | 7.98653 | NaBu/Ct | 1.11198 | 4PBA/Ct | 7.9869 |
| 11742669_s_at | 554313 /// 8370 | HIST2H4A /// HIST2H4B | histone cluster 2, H4a /// histone cluster 2, H4b | NM_001034077 | 7.30285 | NaBu/Ct | 1.49062 | 4PBA/Ct | 6.70038 |
| 11742683_x_at | 3006 | HIST1H1C | histone cluster 1, H1c | NM_005319 | 15.0629 | NaBu/Ct | 2.07611 | 4PBA/Ct | 11.8759 |
| 11742688_s_at | 723790 /8337 | HIST2H2AA3 /HIST2H2AA4 | histone cluster 2, H2aa3 / histone cluster 2, H2aa4 | NM_001040874 | 7.98307 | NaBu/Ct | 1.03252 | 4PBA/Ct | 8.67799 |
| 11742890_at | 84057 | MND1 | meiotic nuclear divisions 1 homolog (S. cerevisiae) | NM_032117 | -11.8928 | NaBu/Ct | -2.60823 | 4PBA/Ct | 8.23339 |
| 11743064_at | 990 | CDC6 | cell division cycle 6 homolog (S. cerevisiae) | NM_001254 | -6.47312 | NaBu/Ct | -3.60927 | 4PBA/Ct | 4.41181 |
| 11743065_at | 990 | CDC6 | cell division cycle 6 homolog (S. cerevisiae) | NM_001254 | -6.49432 | NaBu/Ct | -4.10103 | 4PBA/Ct | 4.43471 |
| 11743160_a_at | 55355 | HJURP | Holliday junction recognition protein | NM_018410 | -19.0009 | NaBu/Ct | -2.61259 | 4PBA/Ct | 11.6622 |
| 11743190_s_at | 10635 | RAD51AP1 | RAD51 associated protein 1 | NM_001130862 | -11.7848 | NaBu/Ct | -4.9241 | 4PBA/Ct | 8.6836 |
| 11743296_a_at | 1063 | CENPF | centromere protein F, 350/400kDa (mitosin) | NM_016343 | -4.81668 | NaBu/Ct | -2.38343 | 4PBA/Ct | 4.30853 |
| 11743367_a_at | 5332 | PLCB4 | phospholipase C, beta 4 | NM_000933 | 4.65283 | NaBu/Ct | 2.2405 | 4PBA/Ct | 2.51361 |
| 11743415_s_at | 65009 | NDRG4 | NDRG family member 4 | NM_001130487 | 6.81913 | NaBu/Ct | 3.55935 | 4PBA/Ct | 5.55021 |
| 11743530_a_at | 64105 | CENPK | centromere protein K | NM_022145 | -8.35439 | NaBu/Ct | -3.41039 | 4PBA/Ct | 7.26506 |
| 11743536_at | 7443 | VRK1 | vaccinia related kinase 1 | NM_003384 | -9.50574 | NaBu/Ct | -3.6136 | 4PBA/Ct | 7.10967 |
| 11743681_at | 140576 | S100A16 | S100 calcium binding protein A16 | NM_080388 | -4.41455 | NaBu/Ct | -1.11982 | 4PBA/Ct | 4.32139 |
| 11743687_s_at | 3148 | HMGB2 | high-mobility group box 2 | NM_001130688 | -7.87808 | NaBu/Ct | -2.63805 | 4PBA/Ct | 5.46032 |
| 11744219_at | 50486 | G0S2 | G0/G1switch 2 | NM_015714 | 3.49593 | NaBu/Ct | 3.02433 | 4PBA/Ct | 2.43023 |
| 11744274_at | 10403 | NDC80 | NDC80 homolog, kinetochore complex component (S. cerevisiae) | NM_006101 | -11.3898 | NaBu/Ct | -3.30456 | 4PBA/Ct | 7.96043 |
| 11744424_a_at | 25984 | KRT23 | keratin 23 (histone deacetylase inducible) | NM_015515 | 7.96304 | NaBu/Ct | 4.93205 | 4PBA/Ct | 5.03712 |
| 11744436_a_at | 11113 | CIT | citron (rho-interacting, serine/threonine kinase 21) | NM_007174 | -5.95255 | NaBu/Ct | -2.45444 | 4PBA/Ct | 4.31511 |
| 11744493_x_at | 25960 | GPR124 | G protein-coupled receptor 124 | NM_032777 | 4.21679 | NaBu/Ct | 2.57766 | 4PBA/Ct | 2.83357 |
| 11744511_x_at | 23421 | ITGB3BP | integrin beta 3 binding protein (beta3-endonexin) | NM_014288 | -4.38254 | NaBu/Ct | -2.09325 | 4PBA/Ct | 2.77505 |
| 11744654_s_at | 960 | CD44 | CD44 molecule (Indian blood group) | NM_000610 | -7.07376 | NaBu/Ct | -1.01926 | 4PBA/Ct | 7.35029 |
| 11744699_a_at | 55775 | TDP1 | tyrosyl-DNA phosphodiesterase 1 | NM_001008744 | -4.92085 | NaBu/Ct | -2.12049 | 4PBA/Ct | 2.93763 |
| 11744700_x_at | 55775 | TDP1 | tyrosyl-DNA phosphodiesterase 1 | NM_001008744 | -3.88335 | NaBu/Ct | -1.82279 | 4PBA/Ct | 2.22936 |
| 11744776_a_at | 4837 | NNMT | nicotinamide N-methyltransferase | NM_006169 | -10.6467 | NaBu/Ct | 1.12457 | 4PBA/Ct | 12.6075 |
| 11744843_s_at | 5069 | PAPPA | pregnancy-associated plasma protein A, pappalysin 1 | NM_002581 | -9.17812 | NaBu/Ct | 1.12572 | 4PBA/Ct | 11.6232 |
| 11745049_a_at | 8318 | CDC45 | cell division cycle 45 homolog (S. cerevisiae) | NM_001178010 | -4.57959 | NaBu/Ct | -2.99523 | 4PBA/Ct | 2.9446 |
| 11745130_x_at | 3149 | HMGB3 | high-mobility group box 3 | NM_005342 | -5.21224 | NaBu/Ct | -1.96986 | 4PBA/Ct | 4.30183 |
| 11745191_a_at | 7205 | TRIP6 | thyroid hormone receptor interactor 6 | NM_003302 | -4.83635 | NaBu/Ct | -1.58775 | 4PBA/Ct | 4.89181 |
| 11745196_a_at | 55256 | ADI1 | acireductone dioxygenase 1 | NM_018269 | -4.09508 | NaBu/Ct | -1.79703 | 4PBA/Ct | 3.00594 |
| 11745226_a_at | 5414 | 4-Sep | septin 4 | NM_001198713 | 10.398 | NaBu/Ct | 4.98286 | 4PBA/Ct | 8.04274 |
| 11745227_x_at | 5414 | 4-Sep | septin 4 | NM_001198713 | 6.68082 | NaBu/Ct | 3.75 | 4PBA/Ct | 5.54381 |
| 11745535_a_at | 23418 | CRB1 | crumbs homolog 1 (Drosophila) | NM_001193640 | -8.75627 | NaBu/Ct | -1.83072 | 4PBA/Ct | 7.12134 |
| 11745535_a_at | 23418 | CRB1 | crumbs homolog 1 (Drosophila) | NM_001193640 | -8.75627 | NaBu/Ct | -1.83072 | 4PBA/Ct | 7.12134 |
| 11745571_a_at | 1058 | CENPA | centromere protein A | NM_001042426 | -7.76602 | NaBu/Ct | -1.81894 | 4PBA/Ct | 6.5243 |
| 11745723_a_at | 378938 | MALAT1 | metastasis associated lung adenocarcinoma transcript 1 (non-protein coding) | NR_002819 | 6.94114 | NaBu/Ct | 1.69676 | 4PBA/Ct | 5.99907 |
| 11745724_at | 378938 | MALAT1 | metastasis associated lung adenocarcinoma transcript 1 (non-protein coding) | NR_002819 | 3.91487 | NaBu/Ct | 1.34446 | 4PBA/Ct | 2.80107 |
| 11745818_x_at | 3005 | H1F0 | H1 histone family, member 0 | NM_005318 | 2.92245 | NaBu/Ct | 1.4886 | 4PBA/Ct | 1.83163 |
| 11745819_a_at | 3005 | H1F0 | H1 histone family, member 0 | NM_005318 | 4.40292 | NaBu/Ct | 1.44169 | 4PBA/Ct | 4.05103 |
| 11745843_a_at | 983 | CDK1 | cyclin-dependent kinase 1 | NM_001170406 | -11.3727 | NaBu/Ct | -2.92065 | 4PBA/Ct | 9.77793 |
| 11745868_a_at | 11004 | KIF2C | kinesin family member 2C | NM_006845 | -8.96936 | NaBu/Ct | -2.44609 | 4PBA/Ct | 6.70808 |
| 11745877_x_at | 3068 | HDGF | hepatoma-derived growth factor | NM_001126050 | -4.44054 | NaBu/Ct | -2.31524 | 4PBA/Ct | 3.03677 |
| 11745948_a_at | 1111 | CHEK1 | CHK1 checkpoint homolog (S. pombe) | NM_001114121 | -8.34504 | NaBu/Ct | -3.01078 | 4PBA/Ct | 6.32338 |
| 11745949_x_at | 1111 | CHEK1 | CHK1 checkpoint homolog (S. pombe) | NM_001114121 | -3.79736 | NaBu/Ct | -2.07435 | 4PBA/Ct | 2.25582 |
| 11746599_a_at | 56675 | NRIP3 | nuclear receptor interacting protein 3 | NM_020645 | 7.54233 | NaBu/Ct | 3.04935 | 4PBA/Ct | 6.17149 |
| 11746616_a_at | 26118 | WSB1 | WD repeat and SOCS box-containing 1 | NM_015626 | 4.60393 | NaBu/Ct | 1.86134 | 4PBA/Ct | 3.29581 |
| 11746775_s_at | 10669 | CGREF1 | cell growth regulator with EF-hand domain 1 | NM_001166239 | 5.67303 | NaBu/Ct | 1.74217 | 4PBA/Ct | 4.79166 |
| 11746917_s_at | 244 | ANXA8 | annexin A8 | NM_001040084 | -5.13447 | NaBu/Ct | -1.95834 | 4PBA/Ct | 3.36086 |
| 11747079_x_at | 230 | ALDOC | aldolase C, fructose-bisphosphate | NM_005165 | 11.6908 | NaBu/Ct | 1.71618 | 4PBA/Ct | 10.7049 |
| 11747298_a_at | 140576 | S100A16 | S100 calcium binding protein A16 | NM_080388 | -5.20295 | NaBu/Ct | -1.19624 | 4PBA/Ct | 4.97892 |
| 11747314_a_at | 54704 | PDP1 | pyruvate dehyrogenase phosphatase catalytic subunit 1 | NM_001161778 | 8.53755 | NaBu/Ct | 3.09499 | 4PBA/Ct | 7.06316 |
| 11747720_a_at | 332 | BIRC5 | baculoviral IAP repeat-containing 5 | NM_001012270 | -7.14468 | NaBu/Ct | -2.29416 | 4PBA/Ct | 5.59965 |
| 11747731_a_at | 10926 | DBF4 | DBF4 homolog (S. cerevisiae) | NM_006716 | -5.48383 | NaBu/Ct | -2.16255 | 4PBA/Ct | 4.63973 |
| 11747943_a_at | 79733 | E2F8 | E2F transcription factor 8 | NM_024680 | -3.03233 | NaBu/Ct | -2.12246 | 4PBA/Ct | 1.5854 |
| 11748104_a_at | 995 | CDC25C | cell division cycle 25 homolog C (S. pombe) | NM_001790 | -4.81724 | NaBu/Ct | -1.83584 | 4PBA/Ct | 3.50705 |
| 11748136_a_at | 4085 | MAD2L1 | MAD2 mitotic arrest deficient-like 1 (yeast) | NM_002358 | -10.694 | NaBu/Ct | -3.52575 | 4PBA/Ct | 7.63575 |
| 11748164_a_at | 1462 | VCAN | versican | NM_001126336 | -5.80189 | NaBu/Ct | -1.52539 | 4PBA/Ct | 6.66249 |
| 11748220_a_at | 22933 | SIRT2 | sirtuin 2 | NM_001193286 | 4.22061 | NaBu/Ct | 1.34558 | 4PBA/Ct | 3.97548 |
| 11748314_a_at | 991 | CDC20 | cell division cycle 20 homolog (S. cerevisiae) | NM_001255 | -3.90771 | NaBu/Ct | -2.97888 | 4PBA/Ct | 2.25108 |
| 11748362_s_at | 8714 | ABCC3 | ATP-binding cassette, sub-family C (CFTR/MRP), member 3 | NM_001144070 | -13.9394 | NaBu/Ct | -1.15191 | 4PBA/Ct | 13.9326 |
| 11748543_a_at | 10628 | TXNIP | thioredoxin interacting protein | NM_006472 | 41.521 | NaBu/Ct | 2.03619 | 4PBA/Ct | 23.5259 |
| 11748544_s_at | 10628 | TXNIP | thioredoxin interacting protein | NM_006472 | 33.394 | NaBu/Ct | 1.7663 | 4PBA/Ct | 20.8751 |
| 11748659_a_at | 2202 | EFEMP1 | EGF-containing fibulin-like extracellular matrix protein 1 | NM_001039348 | 11.5126 | NaBu/Ct | 2.29212 | 4PBA/Ct | 7.94734 |
| 11748728_a_at | 8555 | CDC14B | CDC14 cell division cycle 14 homolog B (S. cerevisiae) | NM_001077181 | 4.50446 | NaBu/Ct | 1.97488 | 4PBA/Ct | 3.10672 |
| 11749190_a_at | 1047 | CLGN | calmegin | NM_001130675 | 7.91684 | NaBu/Ct | 4.20975 | 4PBA/Ct | 5.56774 |
| 11749275_a_at | 8935 | SKAP2 | src kinase associated phosphoprotein 2 | NM_003930 | 4.14395 | NaBu/Ct | 1.67719 | 4PBA/Ct | 2.85229 |
| 11749488_a_at | 56130 | PCDHB6 | protocadherin beta 6 | NM_018939 | 3.86544 | NaBu/Ct | 3.41279 | 4PBA/Ct | 2.45433 |
| 11749510_a_at | 25939 | SAMHD1 | SAM domain and HD domain 1 | NM_015474 | 5.67008 | NaBu/Ct | 2.35177 | 4PBA/Ct | 4.16183 |
| 11749656_a_at | 9824 | ARHGAP11A | Rho GTPase activating protein 11A | NM_014783 | -9.29214 | NaBu/Ct | -3.71916 | 4PBA/Ct | 6.49101 |
| 11749659_s_at | 10808 | HSPH1 | heat shock 105kDa/110kDa protein 1 | NM_006644 | -3.44493 | NaBu/Ct | -1.37488 | 4PBA/Ct | 2.39317 |
| 11749666_a_at | 83737 | ITCH | itchy E3 ubiquitin protein ligase homolog (mouse) | NM_031483 | 3.51264 | NaBu/Ct | 1.44929 | 4PBA/Ct | 2.13941 |
| 11749813_a_at | 4751 | NEK2 | NIMA (never in mitosis gene a)-related kinase 2 | NM_002497 | -4.14234 | NaBu/Ct | -2.32682 | 4PBA/Ct | 2.30357 |
| 11749814_s_at | 4751 | NEK2 | NIMA (never in mitosis gene a)-related kinase 2 | NM_002497 | -3.64833 | NaBu/Ct | -2.25519 | 4PBA/Ct | 2.06005 |
| 11749905_a_at | 3491 | CYR61 | cysteine-rich, angiogenic inducer, 61 | NM_001554 | 5.36767 | NaBu/Ct | -1.99586 | 4PBA/Ct | 10.107 |
| 11749969_a_at | 10098 | TSPAN5 | tetraspanin 5 | NM_005723 | 4.13572 | NaBu/Ct | 3.22577 | 4PBA/Ct | 2.77703 |
| 11750824_a_at | 9055 | PRC1 | protein regulator of cytokinesis 1 | NM_003981 | -6.28294 | NaBu/Ct | -2.3374 | 4PBA/Ct | 4.74225 |
| 11751123_a_at | 8318 | CDC45 | cell division cycle 45 homolog (S. cerevisiae) | NM_001178010 | -4.03607 | NaBu/Ct | -2.30161 | 4PBA/Ct | 2.476 |
| 11751206_s_at | 6790 / 6791 | AURKA /AURKAPS1 | aurora kinase A / aurora kinase A pseudogene 1 | NM_003600 | -12.6273 | NaBu/Ct | -3.54751 | 4PBA/Ct | 8.30674 |
| 11751225_s_at | 1438 | CSF2RA | colony stimulating factor 2 receptor, alpha, low-affinity (granulocyte-macrophage) | NM_001161529 | 5.18462 | NaBu/Ct | 4.7485 | 4PBA/Ct | 4.5021 |
| 11751388_a_at | 2237 | FEN1 | flap structure-specific endonuclease 1 | NM_004111 | -7.59604 | NaBu/Ct | -3.17567 | 4PBA/Ct | 5.81587 |
| 11751389_s_at | 2237 | FEN1 | flap structure-specific endonuclease 1 | NM_004111 | -8.85605 | NaBu/Ct | -3.07946 | 4PBA/Ct | 6.04733 |
| 11751509_a_at | 5954 | RCN1 | reticulocalbin 1, EF-hand calcium binding domain | NM_002901 | -5.74183 | NaBu/Ct | -1.01259 | 4PBA/Ct | 6.14918 |
| 11751805_a_at | 7298 | TYMS | thymidylate synthetase | NM_001071 | -28.1514 | NaBu/Ct | -4.07018 | 4PBA/Ct | 15.6317 |
| 11752175_x_at | 10926 | DBF4 | DBF4 homolog (S. cerevisiae) | NM_006716 | -6.38891 | NaBu/Ct | -2.27814 | 4PBA/Ct | 4.58462 |
| 11752674_a_at | 25803 | SPDEF | SAM pointed domain containing ets transcription factor | NM_012391 | -6.17976 | NaBu/Ct | -1.18837 | 4PBA/Ct | 5.37478 |
| 11752765_s_at | 10628 | TXNIP | thioredoxin interacting protein | NM_006472 | 55.9172 | NaBu/Ct | 3.49096 | 4PBA/Ct | 24.8766 |
| 11753139_s_at | 244 | ANXA8 | annexin A8 | NM_001040084 | -4.34178 | NaBu/Ct | -2.24659 | 4PBA/Ct | 2.69602 |
| 11753308_s_at | 154064 | RAET1G | retinoic acid early transcript 1G | NM_001001788 | 6.08778 | NaBu/Ct | 2.70205 | 4PBA/Ct | 4.93564 |
| 11753421_a_at | 10370 | CITED2 | Cbp/p300-interacting transactivator, with Glu/Asp-rich carboxy-terminal domain, 2 | NM_001168388 | 5.57486 | NaBu/Ct | 1.18948 | 4PBA/Ct | 4.97815 |
| 11753579_a_at | 3572 | IL6ST | interleukin 6 signal transducer (gp130, oncostatin M receptor) | NM_001190981 | 4.66914 | NaBu/Ct | 1.88637 | 4PBA/Ct | 2.53886 |
| 11753600_x_at | 3005 | H1F0 | H1 histone family, member 0 | NM_005318 | 4.945 | NaBu/Ct | 1.64408 | 4PBA/Ct | 3.87324 |
| 11753871_a_at | 2788 | GNG7 | guanine nucleotide binding protein (G protein), gamma 7 | NM_052847 | 6.55695 | NaBu/Ct | 3.01672 | 4PBA/Ct | 5.79198 |
| 11753873_a_at | 440603 | BCL2L15 | BCL2-like 15 | NM_001010922 | -4.66536 | NaBu/Ct | 1.05665 | 4PBA/Ct | 4.17309 |
| 11753874_x_at | 440603 | BCL2L15 | BCL2-like 15 | NM_001010922 | -3.06488 | NaBu/Ct | 1.13855 | 4PBA/Ct | 3.08747 |
| 11753878_s_at | 3572 | IL6ST | interleukin 6 signal transducer (gp130, oncostatin M receptor) | NM_001190981 | 3.91026 | NaBu/Ct | 1.73805 | 4PBA/Ct | 2.09464 |
| 11753879_x_at | 3572 | IL6ST | interleukin 6 signal transducer (gp130, oncostatin M receptor) | NM_001190981 | 4.02538 | NaBu/Ct | 1.79212 | 4PBA/Ct | 2.22446 |
| 11753964_x_at | 10808 | HSPH1 | heat shock 105kDa/110kDa protein 1 | NM_006644 | -4.39715 | NaBu/Ct | -1.56808 | 4PBA/Ct | 3.11955 |
| 11754031_s_at | 1163 | CKS1B | CDC28 protein kinase regulatory subunit 1B | NM_001826 | -10.3636 | NaBu/Ct | -2.26637 | 4PBA/Ct | 7.38493 |
| 11754224_x_at | 10156 | RASA4 | RAS p21 protein activator 4 | NM_001079877 | 10.7139 | NaBu/Ct | 5.37762 | 4PBA/Ct | 7.61297 |
| 11754233_a_at | 22995 | CEP152 | centrosomal protein 152kDa | NM_001194998 | -4.17331 | NaBu/Ct | -1.81598 | 4PBA/Ct | 2.85586 |
| 11754243_a_at | 3014 | H2AFX | H2A histone family, member X | NM_002105 | -5.41791 | NaBu/Ct | -2.85647 | 4PBA/Ct | 3.40815 |
| 11754545_x_at | 5327 | PLAT | plasminogen activator, tissue | NM_000930 | 11.0278 | NaBu/Ct | 3.5959 | 4PBA/Ct | 7.78867 |
| 11754676_a_at | 1062 | CENPE | centromere protein E, 312kDa | NM_001813 | -4.67423 | NaBu/Ct | -1.94404 | 4PBA/Ct | 3.1559 |
| 11754696_x_at | 55839 | CENPN | centromere protein N | NM_001100624 | -6.78865 | NaBu/Ct | -1.89488 | 4PBA/Ct | 4.90874 |
| 11754886_s_at | 23208 | SYT11 | synaptotagmin XI | NM_152280 | 17.8131 | NaBu/Ct | 1.40879 | 4PBA/Ct | 15.9594 |
| 11755205_a_at | 10808 | HSPH1 | heat shock 105kDa/110kDa protein 1 | NM_006644 | -4.10286 | NaBu/Ct | -1.31209 | 4PBA/Ct | 3.04122 |
| 11755206_x_at | 10808 | HSPH1 | heat shock 105kDa/110kDa protein 1 | NM_006644 | -4.00011 | NaBu/Ct | -1.51307 | 4PBA/Ct | 2.75967 |
| 11755469_x_at | 4176 | MCM7 | minichromosome maintenance complex component 7 | NM_005916 | -7.63098 | NaBu/Ct | -3.40062 | 4PBA/Ct | 6.3251 |
| 11755589_a_at | 10202 | DHRS2 | dehydrogenase/reductase (SDR family) member 2 | NM_005794 | 33.4136 | NaBu/Ct | 6.2152 | 4PBA/Ct | 16.9147 |
| 11755605_s_at | 378938 | MALAT1 | metastasis associated lung adenocarcinoma transcript 1 (non-protein coding) | NR_002819 | 6.22572 | NaBu/Ct | 2.19386 | 4PBA/Ct | 4.79013 |
| 11755606_x_at | 10437 | IFI30 | interferon, gamma-inducible protein 30 | NM_006332 | 7.44658 | NaBu/Ct | 1.99529 | 4PBA/Ct | 6.19136 |
| 11755613_a_at | 55612 | FERMT1 | fermitin family member 1 | NM_017671 | -7.19604 | NaBu/Ct | 1.17163 | 4PBA/Ct | 9.97517 |
| 11755614_s_at | 55612 | FERMT1 | fermitin family member 1 | NM_017671 | -4.52023 | NaBu/Ct | 1.3633 | 4PBA/Ct | 6.89273 |
| 11755624_a_at | 113115 | FAM54A | family with sequence similarity 54, member A | NM_001099286 | -8.02427 | NaBu/Ct | -3.31887 | 4PBA/Ct | 7.10264 |
| 11755724_a_at | 4291 | MLF1 | myeloid leukemia factor 1 | NM_001130156 | 9.8604 | NaBu/Ct | 2.73485 | 4PBA/Ct | 8.74965 |
| 11755772_a_at | 2308 | FOXO1 | forkhead box O1 | NM_002015 | 3.47527 | NaBu/Ct | 1.57549 | 4PBA/Ct | 2.26577 |
| 11755791_a_at | 163786 | SASS6 | spindle assembly 6 homolog (C. elegans) | NM_194292 | -5.8637 | NaBu/Ct | -3.06619 | 4PBA/Ct | 3.9347 |
| 11756003_x_at | 3481 | IGF2 | insulin-like growth factor 2 (somatomedin A) | NM_000612 | 24.1375 | NaBu/Ct | 34.7503 | 4PBA/Ct | 16.9743 |
| 11756612_a_at | 580 | BARD1 | BRCA1 associated RING domain 1 | NM_000465 | -4.18509 | NaBu/Ct | -2.03479 | 4PBA/Ct | 3.18406 |
| 11756626_s_at | 11130 | ZWINT | ZW10 interactor | NM_001005413 | -20.9069 | NaBu/Ct | -2.36392 | 4PBA/Ct | 13.662 |
| 11756820_a_at | 3434 | IFIT1 | interferon-induced protein with tetratricopeptide repeats 1 | NM_001548 | 8.08689 | NaBu/Ct | 2.5998 | 4PBA/Ct | 6.47278 |
| 11756823_x_at | 91687 | CENPL | centromere protein L | NM_001127181 | -5.68571 | NaBu/Ct | -2.43764 | 4PBA/Ct | 3.73629 |
| 11756918_a_at | 9493 | KIF23 | kinesin family member 23 | NM_004856 | -7.94847 | NaBu/Ct | -2.76327 | 4PBA/Ct | 6.25767 |
| 11757107_s_at | 112495 | GTF3C6 | general transcription factor IIIC, polypeptide 6, alpha 35kDa | NM_138408 | -8.25139 | NaBu/Ct | -1.72497 | 4PBA/Ct | 8.5776 |
| 11757396_s_at | 244 | ANXA8 | annexin A8 | NM_001040084 | -5.83182 | NaBu/Ct | -2.31326 | 4PBA/Ct | 6.37954 |
| 11757581_x_at | 4501 | MT1X | metallothionein 1X | NM_005952 | 12.0216 | NaBu/Ct | 1.37288 | 4PBA/Ct | 10.7958 |
| 11757751_s_at | 8829 | NRP1 | neuropilin 1 | NM_001024628 | -6.74214 | NaBu/Ct | -1.02345 | 4PBA/Ct | 9.32808 |
| 11758089_s_at | 3161 | HMMR | hyaluronan-mediated motility receptor (RHAMM) | NM_001142556 | -8.96577 | NaBu/Ct | -2.47592 | 4PBA/Ct | 6.84113 |
| 11758108_s_at | 2202 | EFEMP1 | EGF-containing fibulin-like extracellular matrix protein 1 | NM_001039348 | 6.93726 | NaBu/Ct | 1.76904 | 4PBA/Ct | 4.95889 |
| 11758196_s_at | 3488 | IGFBP5 | insulin-like growth factor binding protein 5 | NM_000599 | 3.67201 | NaBu/Ct | 3.11633 | 4PBA/Ct | 2.13946 |
| 11758200_x_at | 1163 | CKS1B | CDC28 protein kinase regulatory subunit 1B | NM_001826 | -9.15075 | NaBu/Ct | -2.31563 | 4PBA/Ct | 6.86281 |
| 11758452_s_at | 55166 | CENPQ | centromere protein Q | NM_018132 | -5.87135 | NaBu/Ct | -2.42815 | 4PBA/Ct | 4.24003 |
| 11758478_s_at | 83879 | CDCA7 | cell division cycle associated 7 | NM_031942 | -5.35114 | NaBu/Ct | -2.39831 | 4PBA/Ct | 4.81079 |
| 11758483_s_at | 91947 | ARRDC4 | arrestin domain containing 4 | NM_183376 | 25.6928 | NaBu/Ct | 3.04497 | 4PBA/Ct | 16.277 |
| 11758499_s_at | 51514 | DTL | denticleless homolog (Drosophila) | NM_016448 | -10.2034 | NaBu/Ct | -3.77549 | 4PBA/Ct | 7.29452 |
| 11758528_s_at | 56130 | PCDHB6 | protocadherin beta 6 | NM_018939 | 5.81856 | NaBu/Ct | 5.69455 | 4PBA/Ct | 4.52281 |
| 11758529_s_at | 1058 | CENPA | centromere protein A | NM_001042426 | -8.80347 | NaBu/Ct | -1.66358 | 4PBA/Ct | 6.76241 |
| 11758536_s_at | 79047 | KCTD15 | potassium channel tetramerisation domain containing 15 | NM_001129994 | -19.3396 | NaBu/Ct | -1.88132 | 4PBA/Ct | 19.0469 |
| 11758933_at | 116441 | TM4SF18 | transmembrane 4 L six family member 18 | NM_001184723 | -17.974 | NaBu/Ct | -2.44753 | 4PBA/Ct | 13.6185 |
| 11758967_s_at | 7980 | TFPI2 | tissue factor pathway inhibitor 2 | NM_006528 | 10.2433 | NaBu/Ct | 1.07983 | 4PBA/Ct | 11.0939 |
| 11759013_at | 1111 | CHEK1 | CHK1 checkpoint homolog (S. pombe) | NM_001114121 | -5.31387 | NaBu/Ct | -2.13763 | 4PBA/Ct | 4.08341 |
| 11759038_at | 3572 | IL6ST | interleukin 6 signal transducer (gp130, oncostatin M receptor) | NM_001190981 | 2.78745 | NaBu/Ct | 1.47319 | 4PBA/Ct | 1.30277 |
| 11759130_at | 137970 | UNC5D | unc-5 homolog D (C. elegans) | NM_080872 | 3.56407 | NaBu/Ct | 3.91321 | 4PBA/Ct | 2.81203 |
| 11759131_at | 8969 | HIST1H2AG | histone cluster 1, H2ag | NM_021064 | 13.3746 | NaBu/Ct | 2.19144 | 4PBA/Ct | 10.8128 |
| 11759151_at | 147841 | SPC24 | SPC24, NDC80 kinetochore complex component, homolog (S. cerevisiae) | NM_182513 | -9.69655 | NaBu/Ct | -3.20573 | 4PBA/Ct | 8.18859 |
| 11759176_at | 51512 | GTSE1 | G-2 and S-phase expressed 1 | NM_016426 | -4.74987 | NaBu/Ct | -2.06458 | 4PBA/Ct | 2.95437 |
| 11759177_at | 4121 | MAN1A1 | mannosidase, alpha, class 1A, member 1 | NM_005907 | 9.80702 | NaBu/Ct | 2.36948 | 4PBA/Ct | 8.63639 |
| 11759295_at | 26298 | EHF | ets homologous factor | NM_012153 | -7.43037 | NaBu/Ct | -1.26053 | 4PBA/Ct | 8.37984 |
| 11759316_at | 4782 | NFIC | Nuclear factor I/C (CCAAT-binding transcription factor) | NM_005597 | -4.06224 | NaBu/Ct | -1.38431 | 4PBA/Ct | 4.33774 |
| 11759760_s_at | 100506979 /// 8347 | HIST1H2BC /// LOC100506979 | histone cluster 1, H2bc /// hypothetical LOC100506979 | NM_003526 | 4.07902 | NaBu/Ct | -1.14569 | 4PBA/Ct | 4.15218 |

Figure S2: 4PBA induces gene changes of more than 4-fold.

| Column ID | Entrez Gene | Gene Symbol | Gene Title | RefSeq Transcript ID | Fold-Change(NaBu/Ct) | Fold-Change(NaBu/Ct) | Fold-Change(4PBA/Ct) | Fold-Change(4PBA/Ct) | SS(Type) |  |
| --- | --- | --- | --- | --- | --- | --- | --- | --- | --- | --- |
| 11716086_a_at | 7837 | PXDN | peroxidasin homolog (Drosophila) | NM_012293 | 2.43671 | NaBu /Ct | 4.283 | 4PBA/Ct | 2.23952 |  |
| 11716503_a_at | 1400 | CRMP1 | collapsin response mediator protein 1 | NM_001014809 | 1.15994 | NaBu /Ct | 2.459 | 4PBA/Ct | 1.0274 |  |
| 11716504_x_at | 1400 | CRMP1 | collapsin response mediator protein 1 | NM_001014809 | 2.08298 | NaBu /Ct | 4.249 | 4PBA/Ct | 2.2424 |  |
| 11716523_at | 6280 | S100A9 | S100 calcium binding protein A9 | NM_002965 | 1.59142 | NaBu //Ct | 11.78 | 4PBA/Ct | 7.15417 |  |
| 11717355_a_at | 9638 | FEZ1 | fasciculation and elongation protein zeta 1 (zygin I) | NM_005103 | 8.69579 | NaBu /Ct | 6.805 | 4PBA/Ct | 6.8809 |  |
| 11717516_s_at | 80303 | EFHD1 | EF-hand domain family, member D1 | NM_025202 | 4.59819 | NaBu/Ct | 4.682 | 4PBA/Ct | 3.5992 |  |
| 11718578_a_at | 2947 | GSTM3 | glutathione S-transferase mu 3 (brain) | NM_000849 | 6.92268 | NaBu/Ct | 5.88268 | 4PBA/Ct | 6.12533 |  |
| 11718588_a_at | 56271 | BEX4 | brain expressed, X-linked 4 | NM_001080425 | 13.3923 | NaBu/Ct | 9.39 | 4PBA/Ct | 9.47742 |  |
| 11718841_s_at | 3576 | IL8 | interleukin 8 | NM_000584 | 2.10182 | NaBu/Ct | 6.825 | 4PBA/Ct | 4.01395 |  |
| 11718848_a_at | 84707 | BEX2 | brain expressed X-linked 2 | NM_001168399 | 9.5419 | NaBu/Ct | 6.763 | 4PBA/Ct | 7.05861 |  |
| 11718927_a_at | 84159 | ARID5B | AT rich interactive domain 5B (MRF1-like) | NM_032199 | 10.6513 | NaBu/Ct | 6.862 | 4PBA/Ct | 7.8079 |  |
| 11719029_at | 118788 | PIK3AP1 | phosphoinositide-3-kinase adaptor protein 1 | NM_152309 | 3.28289 | NaBu/Ct | 6.427 | 4PBA/Ct | 3.69553 |  |
| 11719198_s_at | 894 | CCND2 | cyclin D2 | NM_001759 | 4.66243 | NaBu/Ct | 8.814 | 4PBA/Ct | 5.30851 |  |
| 11719230_at | 5097 | PCDH1 | protocadherin 1 | NM_002587 | 3.18589 | NaBu/Ct | 4.536 | 4PBA/Ct | 2.87412 |  |
| 11719247_at | 202 | AIM1 | absent in melanoma 1 | NM_001624 | 6.8258 | NaBu/Ct | 8.346 | 4PBA/Ct | 5.97734 |  |
| 11719527_a_at | 146330 | FBXL16 | F-box and leucine-rich repeat protein 16 | NM_153350 | 4.09025 | NaBu/Ct | 5.262 | 4PBA/Ct | 3.64907 |  |
| 11719699_a_at | 8318 | CDC45 | cell division cycle 45 homolog (S. cerevisiae) | NM_001178010 | -12.3371 | NaBu/Ct | -5.16 | 4PBA/Ct | 8.26672 |  |
| 11720185_a_at | 4085 | MAD2L1 | MAD2 mitotic arrest deficient-like 1 (yeast) | NM_002358 | -9.73919 | NaBu/Ct | -3.12 | 4PBA/Ct | 7.75654 |  |
| 11720186_s_at | 4085 | MAD2L1 | MAD2 mitotic arrest deficient-like 1 (yeast) | NM_002358 | -9.88458 | NaBu/Ct | -4.89 | 4PBA/Ct | 7.42819 |  |
| 11720240_at | 11013 | TMSB15A | thymosin beta 15a | NM_021992 | 8.52466 | NaBu/Ct | 9.861 | 4PBA/Ct | 7.19831 |  |
| 11720255_a_at | 5414 | 4-Sep | septin 4 | NM_001198713 | 8.34263 | NaBu/Ct | 4.816 | 4PBA/Ct | 7.02551 |  |
| 11720256_x_at | 5414 | 4-Sep | septin 4 | NM_001198713 | 6.38145 | NaBu/Ct | 4.088 | 4PBA/Ct | 5.64752 |  |
| 11721345_at | 26499 | PLEK2 | pleckstrin 2 | NM_016445 | 4.34972 | NaBu/Ct | 5.885 | 4PBA/Ct | 4.17755 |  |
| 11721625_s_at | 2752 | GLUL | glutamate-ammonia ligase | NM_001033044 | 3.32464 | NaBu/Ct | 6.397 | 4PBA/Ct | 3.70089 |  |
| 11721638_s_at | 1066 | CES1 | carboxylesterase 1 | NM_001025194 | 1.64423 | NaBu/Ct | 4.664 | 4PBA/Ct | 2.63253 |  |
| 11721838_a_at | 11010 | GLIPR1 | GLI pathogenesis-related 1 | NM_006851 | 1.38959 | NaBu/Ct | 2.931 | 4PBA/Ct | 1.42776 |  |
| 11721839_at | 11010 | GLIPR1 | GLI pathogenesis-related 1 | NM_006851 | 1.17075 | NaBu/Ct | 2.728 | 4PBA/Ct | 1.36722 |  |
| 11721840_at | 11010 | GLIPR1 | GLI pathogenesis-related 1 | NM_006851 | 1.05381 | NaBu/Ct | 2.179 | 4PBA/Ct | 0.880645 |  |
| 11721842_a_at | 11010 | GLIPR1 | GLI pathogenesis-related 1 | NM_006851 | -1.15907 | NaBu/Ct | 4.477 | 4PBA/Ct | 3.65482 |  |
| 11721887_a_at | 1428 | CRYM | crystallin, mu | NM_001014444 | 4.25286 | NaBu/Ct | 4.226 | 4PBA/Ct | 3.58988 |  |
| 11721911_at | 30812 | SOX8 | SRY (sex determining region Y)-box 8 | NM_014587 | 3.1344 | NaBu/Ct | 6.924 | 4PBA/Ct | 4.25082 |  |
| 11722049_a_at | 1848 | DUSP6 | dual specificity phosphatase 6 | NM_001946 | 2.90747 | NaBu/Ct | 4.036 | 4PBA/Ct | 2.62679 |  |
| 11722571_at | 890 | CCNA2 | cyclin A2 | NM_001237 | -18.6901 | NaBu/Ct | -3.29 | 4PBA/Ct | 11.3636 |  |
| 11722572_at | 890 | CCNA2 | cyclin A2 | NM_001237 | -11.6357 | NaBu/Ct | -4.58 | 4PBA/Ct | 8.2139 |  |
| 11722908_a_at | 90293 | KLHL13 | kelch-like 13 (Drosophila) | NM_001168299 | 3.60336 | NaBu/Ct | 5.55 | 4PBA/Ct | 3.31116 |  |
| 11723033_at | 4856 | NOV | nephroblastoma overexpressed gene | NM_002514 | -1.24797 | NaBu/Ct | 6.834 | 4PBA/Ct | 6.99584 |  |
| 11723047_at | 1381 | CRABP1 | cellular retinoic acid binding protein 1 | NM_004378 | 2.06022 | NaBu/Ct | 5.204 | 4PBA/Ct | 2.85495 |  |
| 11723239_a_at | 2788 | GNG7 | guanine nucleotide binding protein (G protein), gamma 7 | NM_052847 | 17.3326 | NaBu/Ct | 5.465 | 4PBA/Ct | 11.6793 |  |
| 11723245_a_at | 6422 | SFRP1 | secreted frizzled-related protein 1 | NM_003012 | 5.8624 | NaBu/Ct | 5.725 | 4PBA/Ct | 4.83979 |  |
| 11723246_s_at | 6422 | SFRP1 | secreted frizzled-related protein 1 | NM_003012 | 3.42059 | NaBu/Ct | 3.652 | 4PBA/Ct | 2.75502 |  |
| 11723247_a_at | 6422 | SFRP1 | secreted frizzled-related protein 1 | NM_003012 | 2.62186 | NaBu/Ct | 2.306 | 4PBA/Ct | 1.27551 |  |
| 11723377_a_at | 145781 | GCOM1 | GRINL1A complex locus | NM_001018090 | 7.13755 | NaBu/Ct | 9.159 | 4PBA/Ct | 7.67244 |  |
| 11723778_at | 113612 | CYP2U1 | cytochrome P450, family 2, subfamily U, polypeptide 1 | NM_183075 | 4.72632 | NaBu/Ct | 4.057 | 4PBA/Ct | 3.99537 |  |
| 11723829_s_at | 55107 | ANO1 | anoctamin 1, calcium activated chloride channel | NM_018043 | 2.94554 | NaBu/Ct | 6.35 | 4PBA/Ct | 3.60346 |  |
| 11723830_x_at | 55107 | ANO1 | anoctamin 1, calcium activated chloride channel | NM_018043 | 2.36631 | NaBu/Ct | 4.743 | 4PBA/Ct | 2.53151 |  |
| 11724196_s_at | 6781 | STC1 | stanniocalcin 1 | NM_003155 | 3.65583 | NaBu/Ct | 7.545 | 4PBA/Ct | 5.11511 |  |
| 11724197_at | 6781 | STC1 | stanniocalcin 1 | NM_003155 | 4.25542 | NaBu/Ct | 10.02 | 4PBA/Ct | 6.94857 |  |
| 11724198_a_at | 6781 | STC1 | stanniocalcin 1 | NM_003155 | 3.87603 | NaBu/Ct | 5.273 | 4PBA/Ct | 4.20736 |  |
| 11724585_a_at | 6857 | SYT1 | synaptotagmin I | NM_001135805 | 8.78663 | NaBu/Ct | 4.438 | 4PBA/Ct | 6.46019 |  |
| 11724586_a_at | 6857 | SYT1 | synaptotagmin I | NM_001135805 | 5.68106 | NaBu/Ct | 2.602 | 4PBA/Ct | 3.69464 |  |
| 11724605_a_at | 90161 | HS6ST2 | heparan sulfate 6-O-sulfotransferase 2 | NM_001077188 | 2.08208 | NaBu/Ct | 4.404 | 4PBA/Ct | 2.39777 |  |
| 11725176_s_at | 185 | AGTR1 | angiotensin II receptor, type 1 | NM_000685 | 4.58229 | NaBu/Ct | 6.49 | 4PBA/Ct | 4.66933 |  |
| 11725521_x_at | 2752 | GLUL | glutamate-ammonia ligase | NM_001033044 | 3.54348 | NaBu/Ct | 6.581 | 4PBA/Ct | 3.8456 |  |
| 11725599_at | 146227 | BEAN1 | brain expressed, associated with NEDD4, 1 | NM_001136106 | 1.01956 | NaBu/Ct | 4.1 | 4PBA/Ct | 2.84684 |  |
| 11725928_s_at | 4747 | NEFL | neurofilament, light polypeptide | NM_006158 | 1.43521 | NaBu/Ct | 2.583 | 4PBA/Ct | 0.958891 |  |
| 11725929_a_at | 4747 | NEFL | neurofilament, light polypeptide | NM_006158 | 1.87369 | NaBu/Ct | 4.149 | 4PBA/Ct | 2.12089 |  |
| 11726302_a_at | 51514 | DTL | denticleless homolog (Drosophila) | NM_016448 | -12.237 | NaBu/Ct | -5.22 | 4PBA/Ct | 9.0241 |  |
| 11726434_at | 10752 | CHL1 | cell adhesion molecule with homology to L1CAM (close homolog of L1) | NM_006614 | -3.26042 | NaBu/Ct | 4.11 | 4PBA/Ct | 10.9423 |  |
| 11726551_s_at | 154064 | RAET1L / | retinoic acid early transcript 1L /// UL16 binding protein 2 | NM_025217 | 22.0519 | NaBu/Ct | 6.893 | 4PBA/Ct | 14.8644 |  |
| 11726552_x_at | 80328 | ULBP2 | UL16 binding protein 2 | NM_025217 | 19.3083 | NaBu/Ct | 5.498 | 4PBA/Ct | 13.8363 |  |
| 11726590_at | 6785 | ELOVL4 | elongation of very long chain fatty acids (FEN1/Elo2, SUR4/Elo3, yeast)-like 4 | NM_022726 | 6.21653 | NaBu/Ct | 4.94 | 4PBA/Ct | 5.39767 |  |
| 11726591_s_at | 6785 | ELOVL4 | elongation of very long chain fatty acids (FEN1/Elo2, SUR4/Elo3, yeast)-like 4 | NM_022726 | 7.64167 | NaBu/Ct | 5.818 | 4PBA/Ct | 6.23086 |  |
| 11726916_at | 353189 | SLCO4C1 | solute carrier organic anion transporter family, member 4C1 | NM_180991 | 2.3157 | NaBu/Ct | 4.066 | 4PBA/Ct | 2.11196 |  |
| 11727280_a_at | 3757 | KCNH2 | potassium voltage-gated channel, subfamily H (eag-related), member 2 | NM_000238 | 6.20412 | NaBu/Ct | 5.596 | 4PBA/Ct | 5.184 |  |
| 11727445_a_at | 55026 | FAM70A | family with sequence similarity 70, member A | NM_001104544 | 2.55484 | NaBu/Ct | 7.256 | 4PBA/Ct | 4.15251 |  |
| 11728101_at | 195828 | ZNF367 | zinc finger protein 367 | NM_153695 | -4.50553 | NaBu/Ct | -6.34 | 4PBA/Ct | 4.5803 |  |
| 11728308_at | 54474 | KRT20 | keratin 20 | NM_019010 | 12.757 | NaBu/Ct | 5.948 | 4PBA/Ct | 8.72696 |  |
| 11728342_at | 9201 | DCLK1 | doublecortin-like kinase 1 | NM_001195415 | -1.00432 | NaBu/Ct | 7.562 | 4PBA/Ct | 5.818 |  |
| 11728851_s_at | 116442 | RAB39B | RAB39B, member RAS oncogene family | NM_171998 | 16.4698 | NaBu/Ct | 4.046 | 4PBA/Ct | 12.4619 |  |
| 11728991_a_at | 57722 | IGDCC4 | immunoglobulin superfamily, DCC subclass, member 4 | NM_020962 | 2.11729 | NaBu/Ct | 4.176 | 4PBA/Ct | 2.62683 |  |
| 11728992_s_at | 57722 | IGDCC4 | immunoglobulin superfamily, DCC subclass, member 4 | NM_020962 | 1.5418 | NaBu/Ct | 4.122 | 4PBA/Ct | 2.37683 |  |
| 11730121_a_at | 79895 | ATP8B4 | ATPase, class I, type 8B, member 4 | NM_024837 | 1.90324 | NaBu/Ct | 4.093 | 4PBA/Ct | 2.07706 |  |
| 11731433_a_at | 6414 | SEPP1 | selenoprotein P, plasma, 1 | NM_001085486 | 6.31912 | NaBu/Ct | 4.769 | 4PBA/Ct | 5.03488 |  |
| 11731460_at | 1368 | CPM | carboxypeptidase M | NM_001005502 | 6.85141 | NaBu/Ct | 4.299 | 4PBA/Ct | 5.55399 |  |
| 11731461_x_at | 1368 | CPM | carboxypeptidase M | NM_001005502 | 11.7415 | NaBu/Ct | 5.856 | 4PBA/Ct | 9.47681 |  |
| 11731463_x_at | 1368 | CPM | carboxypeptidase M | NM_001005502 | 2.24324 | NaBu/Ct | 2.171 | 4PBA/Ct | 1.11486 |  |
| 11731464_at | 1368 | CPM | carboxypeptidase M | NM_001005502 | 6.04869 | NaBu/Ct | 3.675 | 4PBA/Ct | 5.14262 |  |
| 11731606_at | 85477 | SCIN | scinderin | NM_001112706 | 10.249 | NaBu/Ct | 4.651 | 4PBA/Ct | 6.73611 |  |
| 11731670_a_at | 56130 | PCDHB6 | protocadherin beta 6 | NM_018939 | 4.67413 | NaBu/Ct | 4.398 | 4PBA/Ct | 3.2816 |  |
| 11731782_s_at | 441519 | CT45A1 | cancer/testis antigen family 45, member A1 | NM_001007551 | 14.9207 | NaBu/Ct | 6.511 | 4PBA/Ct | 9.6472 |  |
| 11732191_s_at | 10635 | RAD51AP1 | RAD51 associated protein 1 | NM_001130862 | -14.1588 | NaBu/Ct | -4.14 | 4PBA/Ct | 9.5085 |  |
| 11732193_a_at | 4291 | MLF1 | myeloid leukemia factor 1 | NM_001130156 | 12.6522 | NaBu/Ct | 4.726 | 4PBA/Ct | 9.92438 |  |
| 11732321_a_at | 5327 | PLAT | plasminogen activator, tissue | NM_000930 | 13.1082 | NaBu/Ct | 4.571 | 4PBA/Ct | 9.01504 |  |
| 11732339_at | 53335 | BCL11A | B-cell CLL/lymphoma 11A (zinc finger protein) | NM_018014 | 4.86711 | NaBu/Ct | 5.727 | 4PBA/Ct | 3.86904 |  |
| 11732341_at | 53335 | BCL11A | B-cell CLL/lymphoma 11A (zinc finger protein) | NM_018014 | 3.47488 | NaBu/Ct | 4.1 | 4PBA/Ct | 2.49202 |  |
| 11732425_at | 118932 | ANKRD22 | ankyrin repeat domain 22 | NM_144590 | -1.50286 | NaBu/Ct | 7.615 | 4PBA/Ct | 7.67572 |  |
| 11733042_at | 6542 | SLC7A2 | solute carrier family 7 (cationic amino acid transporter, y+ system), member 2 | NM_001008539 | 1.73909 | NaBu/Ct | 4.257 | 4PBA/Ct | 2.50373 |  |
| 11733044_at | 6542 | SLC7A2 | solute carrier family 7 (cationic amino acid transporter, y+ system), member 2 | NM_001008539 | -1.18927 | NaBu/Ct | 2.482 | 4PBA/Ct | 2.26404 |  |
| 11733045_at | 6542 | SLC7A2 | solute carrier family 7 (cationic amino acid transporter, y+ system), member 2 | NM_001008539 | 1.08286 | NaBu/Ct | 2.95 | 4PBA/Ct | 2.35545 |  |
| 11733071_a_at | 6520 | SLC3A2 | solute carrier family 3 (activators of dibasic and neutral amino acid transport), membe | NM_001012661 | -3.89475 | NaBu/Ct | -2.26 | 4PBA/Ct | 2.29884 |  |
| 11733662_a_at | 3757 | KCNH2 | potassium voltage-gated channel, subfamily H (eag-related), member 2 | NM_000238 | 9.09397 | NaBu/Ct | 7.706 | 4PBA/Ct | 7.37326 |  |
| 11733817_s_at | 2273 | FHL1 | four and a half LIM domains 1 | NM_001159699 | 1.75016 | NaBu/Ct | 3.259 | 4PBA/Ct | 1.46824 |  |
| 11733818_x_at | 2273 | FHL1 | four and a half LIM domains 1 | NM_001159699 | 2.67485 | NaBu/Ct | 5.652 | 4PBA/Ct | 3.15333 |  |
| 11734024_x_at | 284047 | CCDC144A | coiled-coil domain containing 144A /// coiled-coil domain containing 144B | NM_014695 | 7.82506 | NaBu/Ct | 4.289 | 4PBA/Ct | 4.89707 |  |
| 11734295_x_at | 400720 | ZNF772 | zinc finger protein 772 | NM_001024596 | 5.63927 | NaBu/Ct | 5.465 | 4PBA/Ct | 4.22988 |  |
| 11734414_at | 797 | CALCB | calcitonin-related polypeptide beta | NM_000728 | 2.46073 | NaBu/Ct | 4.036 | 4PBA/Ct | 2.31583 |  |
| 11734530_x_at | 3134 | HLA-F | major histocompatibility complex, class I, F | NM_001098478 | 5.4546 | NaBu/Ct | 5.188 | 4PBA/Ct | 4.71337 |  |
| 11734701_a_at | 27122 | DKK3 | dickkopf homolog 3 (Xenopus laevis) | NM_001018057 | 7.08601 | NaBu/Ct | 6.445 | 4PBA/Ct | 6.08479 |  |
| 11735192_a_at | 30817 | EMR2 | egf-like module containing, mucin-like, hormone receptor-like 2 | NM_013447 | 2.05913 | NaBu/Ct | 4.552 | 4PBA/Ct | 2.42149 |  |
| 11735475_at | 3049 | HBQ1 | hemoglobin, theta 1 | NM_005331 | 4.80997 | NaBu/Ct | 5.309 | 4PBA/Ct | 3.95894 |  |
| 11735584_x_at | 672 | BRCA1 | breast cancer 1, early onset | NM_007294 | -6.87484 | NaBu/Ct | -4.11 | 4PBA/Ct | 5.42235 |  |
| 11739489_a_at | 57205 | ATP10D | ATPase, class V, type 10D | NM_020453 | 4.62267 | NaBu/Ct | 4.38 | 4PBA/Ct | 3.52512 |  |
| 11739787_a_at | 3481 / | IGF2 | insulin-like growth factor 2 (somatomedin A) | NM_000612 | 31.0013 | NaBu/Ct | 48.84 | 4PBA/Ct | 20.2622 |  |
| 11739828_s_at | 192668 | CYS1 | cystin 1 | NM_001037160 | 3.70598 | NaBu/Ct | 4.364 | 4PBA/Ct | 2.73174 |  |
| 11739932_a_at | 374393 | FAM111B | family with sequence similarity 111, member B | NM_001142703 | -9.61145 | NaBu/Ct | -4.88 | 4PBA/Ct | 6.16002 |  |
| 11741059_s_at | 6304 | SATB1 | SATB homeobox 1 | NM_001131010 | 3.07442 | NaBu/Ct | 4.604 | 4PBA/Ct | 2.6146 |  |
| 11741492_x_at | 374393 | FAM111B | family with sequence similarity 111, member B | NM_001142703 | -9.30796 | NaBu/Ct | -4.11 | 4PBA/Ct | 6.2452 |  |
| 11741874_x_at | 6414 | SEPP1 | selenoprotein P, plasma, 1 | NM_001085486 | 7.78732 | NaBu/Ct | 6.337 | 4PBA/Ct | 6.14457 |  |
| 11742745_a_at | 123 | PLIN2 | perilipin 2 | NM_001122 | 3.15241 | NaBu/Ct | 5.713 | 4PBA/Ct | 3.93572 |  |
| 11742746_a_at | 123 | PLIN2 | perilipin 2 | NM_001122 | 2.46484 | NaBu/Ct | 5.762 | 4PBA/Ct | 3.81417 |  |
| 11743050_a_at | 29103 | DNAJC15 | DnaJ (Hsp40) homolog, subfamily C, member 15 | NM_013238 | -2.28747 | NaBu/Ct | 4.296 | 4PBA/Ct | 8.92678 |  |
| 11743064_at | 990 | CDC6 | cell division cycle 6 homolog (S. cerevisiae) | NM_001254 | -6.47312 | NaBu/Ct | -3.61 | 4PBA/Ct | 4.41181 |  |
| 11743065_at | 990 | CDC6 | cell division cycle 6 homolog (S. cerevisiae) | NM_001254 | -6.49432 | NaBu/Ct | -4.1 | 4PBA/Ct | 4.43471 |  |
| 11743190_s_at | 10635 | RAD51AP1 | RAD51 associated protein 1 | NM_001130862 | -11.7848 | NaBu/Ct | -4.92 | 4PBA/Ct | 8.6836 |  |
| 11743513_a_at | 51659 | GINS2 | GINS complex subunit 2 (Psf2 homolog) | NM_016095 | -6.32063 | NaBu/Ct | -4.03 | 4PBA/Ct | 4.40622 |  |
| 11743617_at | 3673 | ITGA2 | integrin, alpha 2 (CD49B, alpha 2 subunit of VLA-2 receptor) | NM_002203 | 1.1536 | NaBu/Ct | 4.027 | 4PBA/Ct | 2.45185 |  |
| 11743618_at | 3673 | ITGA2 | integrin, alpha 2 (CD49B, alpha 2 subunit of VLA-2 receptor) | NM_002203 | 1.05824 | NaBu/Ct | 3.093 | 4PBA/Ct | 1.72607 |  |
| 11743692_a_at | 4173 | MCM4 | minichromosome maintenance complex component 4 | NM_005914 | -6.48058 | NaBu/Ct | -4.56 | 4PBA/Ct | 5.11 |  |
| 11743693_a_at | 4173 | MCM4 | minichromosome maintenance complex component 4 | NM_005914 | -5.43261 | NaBu/Ct | -3.32 | 4PBA/Ct | 5.17623 |  |
| 11743815_a_at | 1901 | S1PR1 | sphingosine-1-phosphate receptor 1 | NM_001400 | -1.07798 | NaBu/Ct | 2.588 | 4PBA/Ct | 1.53061 |  |
| 11743816_s_at | 1901 | S1PR1 | sphingosine-1-phosphate receptor 1 | NM_001400 | 1.43822 | NaBu/Ct | 4.879 | 4PBA/Ct | 3.6176 |  |
| 11744351_a_at | 22861 | LOC728392 | hypothetical protein LOC728392 | NM_001033053 | 10.6921 | NaBu/Ct | 4.91 | 4PBA/Ct | 9.31501 |  |
| 11744424_a_at | 25984 | KRT23 | keratin 23 (histone deacetylase inducible) | NM_015515 | 7.96304 | NaBu/Ct | 4.932 | 4PBA/Ct | 5.03712 |  |
| 11744435_a_at | 1848 | DUSP6 | dual specificity phosphatase 6 | NM_001946 | 2.8835 | NaBu/Ct | 4.337 | 4PBA/Ct | 2.73585 |  |
| 11751805_a_at | 7298 | TYMS | thymidylate synthetase | NM_001071 | -28.1514 | NaBu/Ct | -4.07 | 4PBA/Ct | 15.6317 |  |
| 11751905_a_at | 4811 | NID1 | nidogen 1 | NM_002508 | 8.83047 | NaBu/Ct | 8.045 | 4PBA/Ct | 6.59271 |  |
| 11752384_s_at | 1291 | COL6A1 | collagen, type VI, alpha 1 | NM_001848 | 6.62278 | NaBu/Ct | 7.169 | 4PBA/Ct | 5.90385 |  |
| 11754026_a_at | 3576 | IL8 | interleukin 8 | NM_000584 | 2.0679 | NaBu/Ct | 5.759 | 4PBA/Ct | 3.40833 |  |
| 11754224_x_at | 10156 | RASA4 | RAS p21 protein activator 4 | NM_001079877 | 10.7139 | NaBu/Ct | 5.378 | 4PBA/Ct | 7.61297 |  |
| 11754360_a_at | 6241 | RRM2 | ribonucleotide reductase M2 | NM_001034 | -5.3249 | NaBu/Ct | -4.65 | 4PBA/Ct | 4.21467 |  |
| 11754993_s_at | 7837 | PXDN | peroxidasin homolog (Drosophila) | NM_012293 | 3.48362 | NaBu/Ct | 7.765 | 4PBA/Ct | 4.47494 |  |
| 11755367_s_at | 352909 | C19orf51 | chromosome 19 open reading frame 51 | NM_178837 | 6.3388 | NaBu/Ct | 5.778 | 4PBA/Ct | 5.70225 |  |
| 11755933_x_at | 3106 | HLA-B | major histocompatibility complex, class I, B | NM_005514 | 4.33719 | NaBu/Ct | 5.102 | 4PBA/Ct | 3.68692 |  |
| 11755966_x_at | 3106 | HLA-B | major histocompatibility complex, class I, B | NM_005514 | 4.26958 | NaBu/Ct | 4.624 | 4PBA/Ct | 3.49508 |  |
| 11756003_x_at | 3481 | IGF2 | insulin-like growth factor 2 (somatomedin A) | NM_000612 | 24.1375 | NaBu/Ct | 34.75 | 4PBA/Ct | 16.9743 |  |
| 11758031_s_at | 79365 | BHLHE41 | basic helix-loop-helix family, member e41 | NM_030762 | 2.44049 | NaBu/Ct | 5.816 | 4PBA/Ct | 3.4098 |  |
| 11758528_s_at | 56130 | PCDHB6 | protocadherin beta 6 | NM_018939 | 5.81856 | NaBu/Ct | 5.695 | 4PBA/Ct | 4.52281 |  |
| 11758643_s_at | 169834 | ZNF883 | zinc finger protein 883 | NM_001101338 | 4.36434 | NaBu/Ct | 4.801 | 4PBA/Ct | 3.49881 |  |
| 11759134_a_at | 1136 | CHRNA3 | cholinergic receptor, nicotinic, alpha 3 | NM_000743 | 4.52654 | NaBu/Ct | 5.547 | 4PBA/Ct | 3.84325 |  |
| 11759304_at | 7368 | UGT8 | UDP glycosyltransferase 8 | NM_001128174 | 3.90438 | NaBu/Ct | 4.235 | 4PBA/Ct | 3.02614 |  |
| 11763193_at | 284161 | GDPD1 | glycerophosphodiester phosphodiesterase domain containing 1 | NM_001165993 | 6.60456 | NaBu/Ct | 5.526 | 4PBA/Ct | 5.13168 |  |
| 11763226_x_at | 3576 | IL8 | interleukin 8 | NM_000584 | 1.80367 | NaBu/Ct | 5.291 | 4PBA/Ct | 3.15686 |  |
| 11763834_a_at | 151242 | PPP1R1C | protein phosphatase 1, regulatory (inhibitor) subunit 1C | NM_001080545 | 1.41274 | NaBu/Ct | 6.4 | 4PBA/Ct | 5.13986 |  |
| 11763963_at | --- | --- | --- | --- | -1.39892 | NaBu/Ct | 6.389 | 4PBA/Ct | 6.19341 |  |
|  |  |  |  |  |  |  |  |  |  |  |
